# Supplementary material for: Adolescent cardiorespiratory fitness and risk of cancer in late adulthood: A nationwide sibling-controlled cohort study in Sweden
Source: PLoS Med. 2025 May 8;22(5):e1004597. doi: 10.1371/journal.pmed.1004597 (PMC12061154; doi:10.1371/journal.pmed.1004597)
Supplement: S1 Table — (DOCX) [file pmed.1004597.s001.docx]

| **S1 Table. Diagnostic codes used to define the outcomes in the study** | | |
| --- | --- | --- |
| **Cancer outcomes** | **Code Type*** | **Codes** |
| Head and neck | ICD-10 | C00-C14, C30-C32 |
|  | ICD-9 | 140-149 |
|  | ICD-8 | 140-149 |
| Oesophagus | ICD-10 | C15 |
|  | ICD-9 | 150 |
|  | ICD-8 | 150 |
| Lung | ICD-10 | C34 |
|  | ICD-9 | 162 |
|  | ICD-8 | 162 |
| Stomach | ICD-10 | C16 |
|  | ICD-9 | 151 |
|  | ICD-8 | 151 |
| Pancreas | ICD-10 | C25 |
|  | ICD-9 | 157 |
|  | ICD-8 | 157 |
| Liver, bile ducts, and gallbladder | ICD-10 | C22-C24 |
|  | ICD-9 | 155-156 |
|  | ICD-8 | 155-156 |
| Colon | ICD-10 | C18 |
|  | ICD-9 | 153 |
|  | ICD-8 | 153 |
| Rectum | ICD-10 | C19-C20 |
|  | ICD-9 | 154 |
|  | ICD-8 | 154 |
| Kidney | ICD-10 | C64 |
|  | ICD-9 | 189 |
|  | ICD-8 | 189 |
| Prostate | ICD-10 | C61 |
|  | ICD-9 | 185 |
|  | ICD-8 | 185 |
| Bladder | ICD-10 | C67 |
|  | ICD-9 | 188 |
|  | ICD-8 | 188 |
| Myeloma | ICD-10 | C90 |
|  | ICD-9 | 203 |
|  | ICD-8 | 203 |
| Melanoma skin | ICD-10 | C43 |
|  | ICD-9 | 172 |
|  | ICD-8 | 172 |
| Non-melanoma skin | ICD-10 | C44 |
|  | ICD-9 | 173 |
|  | ICD-8 | 173 |
| Overall cancer | ICD-10 | C00-C97 |
|  | ICD-9 | 140-175, 179-208 |
|  | ICD-8 | 140-209 |
| ICD = International Classification of Diseases.  *Codes from all versions (8,9,10) were available for the ascertainment of cancer mortality but codes from versions 8 and 9 were not available for the ascertainment of cancer diagnosis as we did not have access to those codes in the National Patient Register. However, we deem the number of such cases to have been very small given that most of the years of follow-up were covered using ICD-10. Moreover, ICD-8 and 9 are only available in inpatient care and are not validated to the same extent as ICD-10. | | |
