# Supplementary material for: Adolescent cardiorespiratory fitness and risk of cancer in late adulthood: A nationwide sibling-controlled cohort study in Sweden
Source: PLoS Med. 2025 May 8;22(5):e1004597. doi: 10.1371/journal.pmed.1004597 (PMC12061154; doi:10.1371/journal.pmed.1004597)
Supplement: S2 Table — (DOCX) [file pmed.1004597.s002.docx]

| **S2 Table. Baseline characteristics in in the total cohort and the full sibling cohort for those where covariate data was available and for those where covariate data was missing.** | | | | | |
| --- | --- | --- | --- | --- | --- |
|  | **Total cohort** | | | | |
| **Variables** | **All conscripts in the dataset with no exclusions (N=1 354 959)** | **After further excluding those who conscribed before 1972 or after 1995 (N=1 249 131)** | **After further excluding those with missing fitness data (N=1 215 486)** | **After further excluding those with missing covariate data (N=1 143 444)** | **Analytical sample after further excluding those with extreme fitness and BMI values^a^ (N=1 124 049)** |
| **Birth year, median (IQR)** | 1964 (1940, 1978) | 1965 (1940, 1978) | 1965 (1943, 1978) | 1966 (1950, 1978) | 1966 (1960, 1971) |
| **Age at conscription, mean (SD)** | 18.2 (0.9) | 18.2 (0.9) | 18.2 (0.8) | 18.2 (0.7) | 18.3 (0.7) |
| **Cardiorespiratory fitness, Watt max** |  |  |  |  |  |
| Median (range) | 261 (0, 999) | 269 (0, 999) | 269 (0, 999) | 270 (0, 999) | 271 (100, 999) |
| **W_max_^b^ by quartiles, median (range)** |  |  |  |  |  |
| Quartile 1 | 188 (0, 222) | 207 (0, 222) | 207 (0, 222) | 209 (0, 222) | 217 (100, 236) |
| Quartile 2 | 241 (223, 261) | 241 (223, 261) | 241 (223, 261) | 241 (223, 261) | 253 (237, 270) |
| Quartile 3 | 280 (262, 305) | 280 (262, 305) | 280 (262, 305) | 280 (262, 305) | 290 (271, 312) |
| Quartile 4 | 335 (306, 999) | 335 (306, 999) | 335 (306, 999) | 335 (306, 999) | 339 (313, 999) |
| **BMI (kg/m^2^), median (range)** | 21.3 (0, 3609.5) | 21.3 (0, 288.9) | 21.3 (0, 288.9) | 21.3 (0, 288.9) | 21.4 (15.0, 59.2) |
|  | **Full sibling cohort** | | | | |
| **Variables** | **All conscripts in the dataset with no exclusions (N=580 868)** | **After further excluding those who conscribed before 1972 or after 1995 (N=547 913)** | **After further excluding those with missing fitness data (N=536 961)** | **After further excluding those with missing covariate data (N=518 799)** | **Analytical sample after further excluding those with extreme fitness and BMI values^a^ (N=477 453)** |
| **Birth year, median (IQR)** | 1964 (1950, 1978) | 1965 (1950, 1978) | 1965 (1950, 1977) | 1965 (1950, 1977) | 1965 (1961, 1970) |
| **Age at conscription, mean (SD)** | 18.2 (0.7) | 18.2 (0.7) | 18.2 (0.7) | 18.2 (0.7) | 18.3 (0.7) |
| **Cardiorespiratory fitness, Watt max** |  |  |  |  |  |
| Median (range) | 262 (0, 999) | 269 (0, 999) | 269 (0, 999) | 270 (0, 999) | 271 (100, 999) |
| **W_max_^b^ by quartiles, median (range)** |  |  |  |  |  |
| Quartile 1 | 206 (0, 229) | 211 (0, 229) | 211 (0, 229) | 211 (0, 229) | 217 (100, 236) |
| Quartile 2 | 245 (230, 262) | 245 (230, 262) | 245 (230, 262) | 245 (230, 262) | 253 (237, 270) |
| Quartile 3 | 282 (263, 306) | 282 (263, 306) | 282 (263, 306) | 282 (263, 306) | 290 (271, 312) |
| Quartile 4 | 335 (307, 999) | 335 (307, 999) | 335 (307, 999) | 335 (307, 999) | 339 (313, 999) |
| **BMI (kg/m^2^), median (range)** | 21.2 (0, 3609.5) | 21.3 (0, 288.9) | 21.3 (0, 288.9) | 21.3 (0, 288.9) | 21.3 (15.0, 59.2) |
| ^a^Those with <100 W_max_ and BMI <15 or >60 kg/m^2^.  ^b^W_max_ is the maximum number of watts of resistance achieved on a maximal ergometer bicycle test with gradually increasing resistance. BMI = body mass index. IQR = interquartile range. SD = standard deviation. W_max_ = watt maximum. | | | | | |
