# Supplementary material for: Adolescent cardiorespiratory fitness and risk of cancer in late adulthood: A nationwide sibling-controlled cohort study in Sweden
Source: PLoS Med. 2025 May 8;22(5):e1004597. doi: 10.1371/journal.pmed.1004597 (PMC12061154; doi:10.1371/journal.pmed.1004597)
Supplement: S3 Table — (DOCX) [file pmed.1004597.s003.docx]

| **S3 Table. Follow-up time, number of events, and numbers censored in cohort and sibling analysis.** | | |
| --- | --- | --- |
| **Cancer outcome** | **Cohort analysis  (N=1 124 049)** | **Sibling analysis  (N=477 453)** |
| **Overall cancer diagnosis** |  |  |
| Follow-up time, median (range) | 37.7 (0.1, 51.4) | 38.3 (0.1, 51.4) |
| Events | 98 410 (8.8) | 41 293 (8.7) |
| Death from non-cancer causes | 47 320 (4.2) | 18 579 (3.9) |
| Emigration | 75 786 (6.7) | 31 195 (6.5) |
| End of follow-up | 902 533 (80.3) | 386 386 (80.9) |
| **Overall cancer mortality** |  |  |
| Follow-up time, median (range) | 38.3 (0.1, 51.4) | 38.9 (0.1, 51.4) |
| Events | 16 789 (1.5) | 6908 (1.5) |
| Death from non-cancer causes | 48 122 (4.3) | 18 912 (4.0) |
| Emigration | 76 274 (6.8) | 31 377 (6.6) |
| End of follow-up | 982 864 (87.4) | 420 256 (88.0) |
| **Site-specific cancer (diagnosis or death)** |  |  |
| **Head and neck** |  |  |
| Follow-up time, median (range) | 38.3 (0.1, 51.4) | 38.9 (0.1, 51.4) |
| Events | 4026 (0.4) | 1692 (0.4) |
| Death from other causes | 63 867 (5.7) | 25 385 (5.3) |
| Emigration | 76 251 (6.8) | 31 370 (6.6) |
| End of follow-up | 979 905 (87.2) | 419 006 (87.8) |
| **Oesophagus** |  |  |
| Follow-up time, median (range) | 38.3 (0.1, 51.4) | 38.9 (0., 51.4) |
| Events | 1178 (0.1) | 464 (0.1) |
| Death from other causes | 64 069 (5.7) | 25 494 (5.3) |
| Emigration | 76 274 (6.8) | 31 377 (6.6) |
| End of follow-up | 982 528 (87.4) | 420 118 (88.0) |
| **Lung** |  |  |
| Follow-up time, median (range) | 38.3 (0.1, 51.4) | 38.9 (0.1, 51.4) |
| Events | 3131 (0.3) | 1263 (0.3) |
| Death from other causes | 62 755 (5.6) | 24 971 (5.2) |
| Emigration | 76 270 (6.8) | 31 375 (6.6) |
| End of follow-up | 981 893 (87.4) | 419 844 (87.9) |
| **Stomach** |  |  |
| Follow-up time, median (range) | 38.3 (0.1, 51.4) | 38.9 (0.1, 51.4) |
| Events | 1430 (0.1) | 599 (0.1) |
| Death from other causes | 63 986 (5.7) | 25 443 (5.3) |
| Emigration | 76 272 (6.8) | 31 375 (6.6) |
| End of follow-up | 982 361 (87.4) | 420 036 (88.0) |
| **Pancreas** |  |  |
| Follow-up time, median (range) | 38.3 (0.1, 51.4) | 38.9 (0.1, 51.4) |
| Events | 2255 (0.2) | 943 (0.2) |
| Death from other causes | 63 231 (5.6) | 25 134 (5.3) |
| Emigration | 76 273 (6.8) | 31 377 (6.6) |
| End of follow-up | 982 290 (87.4) | 419 999 (88.0) |
| **Liver, bile ducts, and gallbladder** |  |  |
| Follow-up time, median (range) | 38.3 (0.1, 51.4) | 38.9 (0.1, 51.4) |
| Events | 2246 (0.2) | 925 (0.2) |
| Death from other causes | 63 322 (5.6) | 25 160 (5.3) |
| Emigration | 76 270 (6.8) | 31 375 (6.6) |
| End of follow-up | 982 211 (87.4) | 419 993 (88.0) |
| **Colon** |  |  |
| Follow-up time, median (range) | 38.3 (0.1, 51.4) | 38.9 (0.1, 51.4) |
| Events | 5320 (0.5) | 2177 (0.5) |
| Death from other causes | 63 009 (5.6) | 25 046 (5.3) |
| Emigration | 76 259 (6.8) | 31 374 (6.6) |
| End of follow-up | 979 461 (87.1) | 418 856 (87.7) |
| **Rectum** |  |  |
| Follow-up time, median (range) | 38.3 (0.1, 51.4) | 38.9 (0.1, 51.4) |
| Events | 3917 (0.4) | 1625 (0.3) |
| Death from other causes | 63 684 (5.7) | 25 304 (5.3) |
| Emigration | 76 267 (6.8) | 31 372 (6.6) |
| End of follow-up | 980 181 (87.2) | 419 152 (87.8) |
| **Kidney** |  |  |
| Follow-up time, median (range) | 38.3 (0.1, 51.4) | 38.9 (0.1, 51.4) |
| Events | 2741 (0.2) | 1151 (0.2) |
| Death from other causes | 64 174 (5.7) | 25 519 (5.3) |
| Emigration | 76 267 (6.8) | 31 373 (6.6) |
| End of follow-up | 980 867 (87.3) | 419 410 (87.8) |
| **Prostate** |  |  |
| Follow-up time, median (range) | 38.2 (0.1, 51.4) | 38.8 (0.1, 51.4) |
| Events | 24 225 (2.2) | 10 042 (2.1) |
| Death from other causes | 63 387 (5.6) | 25 229 (5.3) |
| Emigration | 76 211 (6.8) | 31 353 (6.6) |
| End of follow-up | 960 225 (85.4) | 410 829 (86.1) |
| **Bladder** |  |  |
| Follow-up time, median (range) | 38.3 (0.1, 51.4) | 38.9 (0.1, 51.4) |
| Events | 3490 (0.3) | 1432 (0.3) |
| Death from other causes | 64 374 (5.7) | 25 594 (5.4) |
| Emigration | 76 256 (6.8) | 31 367 (6.6) |
| End of follow-up | 979 929 (87.2) | 419 060 (87.8) |
| **Myeloma** |  |  |
| Follow-up time, median (range) | 38.3 (0.1, 51.4) | 38.9 (0.1, 51.4) |
| Events | 1460 (0.1) | 580 (0.1) |
| Death from other causes | 64 446 (5.7) | 25 643 (5.4) |
| Emigration | 76 271 (6.8) | 31 374 (6.6) |
| End of follow-up | 981 872 (87.4) | 419 856 (87.9) |
| **Melanoma** |  |  |
| Follow-up time, median (range) | 38.2 (0.1, 51.4) | 38.8 (0.1, 51.4) |
| Events | 10 026 (0.9) | 4216 (0.9) |
| Death from other causes | 63 730 (5.7) | 25 360 (5.3) |
| Emigration | 76 207 (6.8) | 31 359 (6.6) |
| End of follow-up | 974 086 (86.7) | 416 518 (87.2) |
| **Non-melanoma** |  |  |
| Follow-up time, median (range) | 38.1 (0.1, 51.4) | 38.7 (0.1, 51.4) |
| Events | 27 302 (2.4) | 11 509 (2.4) |
| Death from other causes | 63 579 (5.7) | 25 284 (5.3) |
| Emigration | 76 111 (6.8) | 31 313 (6.6) |
| End of follow-up | 957 057 (85.1) | 409 347 (85.7) |
| Number of events and numbers censored are shown as n (%). | | |
