# Supplementary material for: Adolescent cardiorespiratory fitness and risk of cancer in late adulthood: A nationwide sibling-controlled cohort study in Sweden
Source: PLoS Med. 2025 May 8;22(5):e1004597. doi: 10.1371/journal.pmed.1004597 (PMC12061154; doi:10.1371/journal.pmed.1004597)
Supplement: S4 Table — (DOCX) [file pmed.1004597.s004.docx]

| **S4 Table. Follow-up time, cancer-specific deaths, and numbers censored for site-specific mortality in cohort and sibling analysis.** | | |
| --- | --- | --- |
| **Cancer outcome** | **Cohort analysis  (N=1 124 049)** | **Sibling analysis  (N=477 453)** |
| **Head and neck** |  |  |
| Follow-up time, median (range) | 38.3 (0.1, 51.4) | 38.9 (0.1, 51.4) |
| Cancers-specific death | 687 (0.06) | 285 (0.06) |
| Death from other causes | 64 224 (5.7) | 25 535 (5.4) |
| Emigration | 76 274 (6.8) | 31 377 (6.6) |
| End of follow-up | 982 864 (87.4) | 420 256 (88.0) |
| **Oesophagus** |  |  |
| Follow-up time, median (range) | 38.3 (0.1, 51.4) | 38.9 (0.1, 51.4) |
| Cancers-specific death | 654 (0.06) | 247 (0.05) |
| Death from other causes | 64 257 (5.7) | 25 573 (5.4) |
| Emigration | 76 274 (6.8) | 31 377 (6.6) |
| End of follow-up | 982 864 (87.4) | 420 256 (88.0) |
| **Lung** |  |  |
| Follow-up time, median (range) | 38.3 (0.1, 51.4) | 38.9 (0.1, 51.4) |
| Cancers-specific death | 1770 (0.2) | 695 (0.2) |
| Death from other causes | 63 141 (5.6) | 25 705 (5.4) |
| Emigration | 76 274 (6.8) | 31 377 (6.6) |
| End of follow-up | 982 864 (87.4) | 420 256 (88.0) |
| **Stomach** |  |  |
| Follow-up time, median (range) | 38.3 (0.1, 51.4) | 38.9 (0.1, 51.4) |
| Cancers-specific death | 614 (0.05) | 264 (0.06) |
| Death from other causes | 64 297 (5.7) | 25 556 (5.4) |
| Emigration | 76 274 (6.8) | 31 377 (6.6) |
| End of follow-up | 982 864 (87.4) | 420 256 (88.0) |
| **Pancreas** |  |  |
| Follow-up time, median (range) | 38.3 (0.1, 51.4) | 38.9 (0.1, 51.4) |
| Cancers-specific death | 1493 (0.1) | 617 (0.1) |
| Death from other causes | 63 418 (5.6) | 25 203 (5.3) |
| Emigration | 76 274 (6.8) | 31 377 (6.6) |
| End of follow-up | 982 864 (87.4) | 420 256 (88.0) |
| **Liver, bile ducts, and gallbladder** |  |  |
| Follow-up time, median (range) | 38.3 (0.1, 51.4) | 38.9 (0.1, 51.4) |
| Cancers-specific death | 1256 (0.1) | 521 (0.1) |
| Death from other causes | 63 655 (5.7) | 25 299 (5.3) |
| Emigration | 76 274 (6.8) | 31 377 (6.6) |
| End of follow-up | 982 864 (87.4) | 420 256 (88.0) |
| **Colon** |  |  |
| Follow-up time, median (range) | 38.3 (0.1, 51.4) | 38.9 (0.1, 51.4) |
| Cancers-specific death | 1437 (0.1) | 578 (0.1) |
| Death from other causes | 63 474 (5.7) | 25 242 (5.3) |
| Emigration | 76 274 (6.8) | 31 377 (6.6) |
| End of follow-up | 982 864 (87.4) | 420 256 (88.0) |
| **Rectum** |  |  |
| Follow-up time, median (range) | 38.3 (0.1, 51.4) | 38.9 (0.1, 51.4) |
| Cancers-specific death | 848 (0.08) | 367 (0.08) |
| Death from other causes | 64 063 (5.7) | 25 453 (5.3) |
| Emigration | 76 274 (6.8) | 31 377 (6.6) |
| End of follow-up | 982 864 (87.4) | 420 256 (88.0) |
| **Kidney** |  |  |
| Follow-up time, median (range) | 38.3 (0.1, 51.4) | 38.9 (0.1, 51.4) |
| Cancers-specific death | 542 (0.05) | 219 (0.05) |
| Death from other causes | 64 369 (5.7) | 25 601 (5.4) |
| Emigration | 76 264 (6.8) | 31 377 (6.6) |
| End of follow-up | 982 864 (87.4) | 420 256 (88.0) |
| **Prostate** |  |  |
| Follow-up time, median (range) | 38.3 (0.1, 51.4) | 38.9 (0.1, 51.4) |
| Cancers-specific death | 868 (0.08) | 345 (0.07) |
| Death from other causes | 64 043 (5.7) | 25 475 (5.3) |
| Emigration | 76 211 (6.8) | 31 377 (6.6) |
| End of follow-up | 982 864 (87.4) | 420 256 (88.0) |
| **Bladder** |  |  |
| Follow-up time, median (range) | 38.3 (0.1, 51.4) | 38.9 (0.1, 51.4) |
| Cancers-specific death | 280 (0.02) | 115 (0.02) |
| Death from other causes | 64 631 (5.8) | 25 705 (5.4) |
| Emigration | 76 274 (6.8) | 31 377 (6.6) |
| End of follow-up | 982 864 (87.4) | 420 256 (88.0) |
| **Myeloma** |  |  |
| Follow-up time, median (range) | 38.3 (0.1, 51.4) | 38.9 (0.1, 51.4) |
| Cancers-specific death | 360 (0.03) | 134 (0.03) |
| Death from other causes | 64 551 (5.7) | 25 686 (5.4) |
| Emigration | 76 274 (6.8) | 31 377 (6.6) |
| End of follow-up | 982 864 (87.4) | 420 256 (88.0) |
| **Melanoma** |  |  |
| Follow-up time, median (range) | 38.3 (0.1, 51.4) | 38.9 (0.1, 51.4) |
| Cancers-specific death | 875 (0.08) | 344 (0.07) |
| Death from other causes | 64 036 (5.7) | 25 476 (5.3) |
| Emigration | 76 274 (6.8) | 31 377 (6.6) |
| End of follow-up | 982 846 (87.4) | 420 256 (88.0) |
| **Non-melanoma** |  |  |
| Follow-up time, median (range) | 38.3 (0.1, 51.4) | 38.9 (0.1, 51.4) |
| Cancers-specific death | 227 (0.02) | 98 (0.02) |
| Death from other causes | 64 484 (5.8) | 25 722 (5.4) |
| Emigration | 76 274 (6.8) | 31 377 (6.6) |
| End of follow-up | 982 846 (87.4) | 420 256 (88.0) |
| Number of cancer-specific deaths and numbers censored are shown as n (%). | | |
