# Supplementary material for: Adolescent cardiorespiratory fitness and risk of cancer in late adulthood: A nationwide sibling-controlled cohort study in Sweden
Source: PLoS Med. 2025 May 8;22(5):e1004597. doi: 10.1371/journal.pmed.1004597 (PMC12061154; doi:10.1371/journal.pmed.1004597)
Supplement: S5 Table — (DOCX) [file pmed.1004597.s005.docx]

| **S5 Table**. **Hazard ratios for cancer by quartiles of cardiorespiratory fitness in cohort and sibling analysis.** | | | | | | |
| --- | --- | --- | --- | --- | --- | --- |
|  | **Cohort analysis (N=1 124 049)** | |  | **Sibling analysis (N=477 453)** | | |
| **Cancer outcome by quartiles of cardiorespiratory fitness** | **Cases, n (%)** | **HR (95% CI)** | **P-value** | **Cases, n (%)** | **HR (95% CI)** | **P-value** |
| **Overall cancer diagnosis** | | | | | | |
| Q1 | 33 673 (12.0) | Ref. | - | 13 621 (11.5) | Ref. | - |
| Q2 | 28 078 (9.8) | 1.01 (0.99, 1.02) | 0.536 | 11 693 (9.6) | 1.00 (0.96, 1.03) | 0.802 |
| Q3 | 20 523 (7.4) | 1.03 (1.01, 1.05) | 0.002 | 8990 (7.6) | 1.01 (0.97, 1.06) | 0.504 |
| Q4 | 16 136 (5.8) | 1.08 (1.06, 1.11) | <0.001 | 6989 (5.9) | 1.00 (0.95, 1.06) | 0.921 |
| **Overall cancer mortality** | | | | | | |
| Q1 | 7093 (2.5) | Ref. | - | 2790 (2.4) | Ref. | - |
| Q2 | 4810 (1.7) | 0.83 (0.80, 0.86) | <0.001 | 1981 (1.6) | 0.88 (0.81, 0.96) | 0.005 |
| Q3 | 2960 (1.1) | 0.76 (0.73, 0.80) | <0.001 | 1300 (1.1) | 0.85 (0.77, 0.95) | 0.002 |
| Q4 | 1926 (0.7) | 0.71 (0.67, 0.76) | <0.001 | 837 (0.7) | 0.78 (0.68, 0.89) | <0.001 |
| **Site-specific cancers (diagnosis or death)** | | | | | | |
| Head and neck | | | | | | |
| Q1 | 1539 (0.6) | Ref. | - | 629 (0.5) | Ref. | - |
| Q2 | 1148 (0.4) | 0.85 (0.79, 0.92) | <0.001 | 474 (0.4) | 0.93 (0.78, 1.12) | 0.459 |
| Q3 | 782 (0.3) | 0.80 (0.73, 0.88) | <0.001 | 350 (0.3) | 0.84 (0.68, 1.04) | 0.106 |
| Q4 | 557 (0.2) | 0.75 (0.67, 0.84) | <0.001 | 237 (0.2) | 0.80 (0.61, 1.04) | 0.099 |
| Oesophagus | | | | | | |
| Q1 | 536 (0.2) | Ref. | - | 213 (0.2) | Ref. | - |
| Q2 | 346 (0.1) | 0.75 (0.65, 0.86) | <0.001 | 139 (0.1) | 0.95 (0.68, 1.32) | 0.758 |
| Q3 | 185 (0.07) | 0.60 (0.50, 0.72) | <0.001 | 68 (0.06) | 0.68 (0.45, 1.03) | 0.067 |
| Q4 | 111 (0.04) | 0.53 (0.42, 0.66) | <0.001 | 44 (0.04) | 0.61 (0.36, 1.02) | 0.060 |
| Lung | | | | | | |
| Q1 | 1562 (0.6) | Ref. | - | 620 (0.5) | Ref. | - |
| Q2 | 871 (0.3) | 0.74 (0.68, 0.80) | <0.001 | 350 (0.3) | 0.81 (0.67, 0.98) | 0.031 |
| Q3 | 460 (0.2) | 0.62 (0.55, 0.69) | <0.001 | 188 (0.2) | 0.72 (0.56, 0.93) | 0.012 |
| Q4 | 238 (0.09) | 0.49 (0.42, 0.57) | <0.001 | 105 (0.09) | 0.50 (0.36, 0.70) | <0.001 |
| Stomach | | | | | | |
| Q1 | 597 (0.2) | Ref. | - | 234 (0.2) | Ref. | - |
| Q2 | 405 (0.1) | 0.79 (0.70, 0.90) | <0.001 | 164 (0.1) | 0.90 (0.67, 1.21) | 0.487 |
| Q3 | 256 (0.09) | 0.74 (0.63, 0.86) | <0.001 | 101 (0.08) | 0.69 (0.49, 0.97) | 0.035 |
| Q4 | 172 (0.06) | 0.70 (0.58, 0.86) | <0.001 | 79 (0.07) | 0.91 (0.60, 1.37) | 0.649 |
| Pancreas | | | | | | |
| Q1 | 925 (0.3) | Ref. | - | 369 (0.3) | Ref. | - |
| Q2 | 663 (0.2) | 0.86 (0.77, 0.95) | 0.004 | 270 (0.2) | 1.05 (0.83, 1.33) | 0.676 |
| Q3 | 424 (0.2) | 0.84 (0.74, 0.95) | 0.005 | 191 (0.2) | 0.98 (0.74, 1.30) | 0.909 |
| Q4 | 243 (0.09) | 0.73 (0.62, 0.85) | <0.001 | 113 (0.1) | 0.83 (0.59, 1.18) | 0.298 |
| Liver, bile ducts, and gallbladder | | | | | | |
| Q1 | 1038 (0.4) | Ref. | - | 380 (0.3) | Ref. | - |
| Q2 | 657 (0.2) | 0.77 (0.69, 0.85) | <0.001 | 294 (0.2) | 1.10 (0.86, 1.39) | 0.451 |
| Q3 | 333 (0.1) | 0.60 (0.52, 0.68) | <0.001 | 163 (0.1) | 0.97 (0.73, 1.28) | 0.824 |
| Q4 | 218 (0.08) | 0.59 (0.50, 0.69) | <0.001 | 88 (0.07) | 0.78 (0.54, 1.13) | 0.185 |
| Colon | | | | | | |
| Q1 | 2001 (0.7) | Ref. | - | 801 (0.7) | Ref. | - |
| Q2 | 1543 (0.5) | 0.91 (0.85, 0.97) | 0.005 | 618 (0.5) | 0.89 (0.76, 1.04) | 0.134 |
| Q3 | 1050 (0.4) | 0.84 (0.78, 0.91) | <0.001 | 446 (0.4) | 0.92 (0.76, 1.11) | 0.369 |
| Q4 | 726 (0.3) | 0.75 (0.68, 0.83) | <0.001 | 312 (0.3) | 0.78 (0.62, 0.99) | 0.038 |
| Rectum | | | | | | |
| Q1 | 1474 (0.5) | Ref. | - | 615 (0.5) | Ref. | - |
| Q2 | 1154 (0.4) | 0.94 (0.87, 1.02) | 0.120 | 470 (0.4) | 0.90 (0.75, 1.07) | 0.231 |
| Q3 | 764 (0.3) | 0.88 (0.80, 0.96) | 0.006 | 325 (0.3) | 0.84 (0.68, 1.04) | 0.115 |
| Q4 | 525 (0.2) | 0.81 (0.72, 0.91) | <0.001 | 215 (0.2) | 0.69 (0.53, 0.90) | 0.006 |
| Kidney | | | | | | |
| Q1 | 1035 (0.4) | Ref. | - | 416 (0.4) | Ref. | - |
| Q2 | 801 (0.3) | 0.84 (0.76, 0.92) | <0.001 | 352 (0.3) | 1.01 (0.81, 1.26) | 0.904 |
| Q3 | 529 (0.2) | 0.73 (0.65, 0.82) | <0.001 | 226 (0.2) | 0.91 (0.70, 1.18) | 0.470 |
| Q4 | 376 (0.1) | 0.65 (0.57, 0.74) | <0.001 | 157 (0.1) | 0.86 (0.63, 1.18) | 0.350 |
| Prostate | | | | | | |
| Q1 | 9678 (3.4) | Ref. | - | 3887 (3.3) | Ref. | - |
| Q2 | 7507 (2.6) | 1.04 (1.01, 1.07) | 0.015 | 3061 (2.5) | 1.00 (0.93, 1.08) | 0.925 |
| Q3 | 4449 (1.6) | 1.05 (1.01, 1.09) | 0.012 | 1947 (1.6) | 1.03 (0.94, 1.12) | 0.563 |
| Q4 | 2591 (0.9) | 1.10 (1.05, 1.16) | <0.001 | 1147 (1.0) | 1.01 (0.90, 1.13) | 0.867 |
| Bladder | | | | | | |
| Q1 | 1427 (0.5) | Ref. | - | 589 (0.5) | Ref. | - |
| Q2 | 1018 (0.4) | 0.90 (0.82, 0.97) | 0.009 | 405 (0.3) | 0.98 (0.81, 1.19) | 0.832 |
| Q3 | 667 (0.2) | 0.89 (0.81, 0.99) | 0.027 | 270 (0.2) | 0.85 (0.68, 1.05) | 0.135 |
| Q4 | 378 (0.1) | 0.74 (0.65, 0.85) | <0.001 | 168 (0.1) | 0.88 (0.66, 1.19) | 0.418 |
| Myeloma | | | | | | |
| Q1 | 516 (0.2) | Ref. | - | 199 (0.2) | Ref. | - |
| Q2 | 429 (0.2) | 0.99 (0.86, 1.13) | 0.841 | 166 (0.1) | 0.76 (0.55, 1.04) | 0.082 |
| Q3 | 301 (0.1) | 1.00 (0.86, 1.17) | 0.957 | 123 (0.1) | 0.83 (0.57, 1.20) | 0.324 |
| Q4 | 214 (0.08) | 1.00 (0.83, 1.20) | 0.975 | 92 (0.08) | 0.80 (0.50, 1.29) | 0.367 |
| Melanoma skin | | | | | | |
| Q1 | 2622 (0.9) | Ref. | - | 1031 (0.9) | Ref. | - |
| Q2 | 2682 (0.9) | 1.13 (1.07, 1.19) | <0.001 | 1113 (0.9) | 1.08 (0.96, 1.22) | 0.206 |
| Q3 | 2396 (0.9) | 1.28 (1.21, 1.36) | <0.001 | 1067 (0.9) | 1.22 (1.06, 1.40) | 0.004 |
| Q4 | 2326 (0.8) | 1.50 (1.41, 1.61) | <0.001 | 1005 (0.9) | 1.30 (1.10, 1.52) | 0.001 |
| Non-melanoma skin | | | | | | |
| Q1 | 8441 (3.0) | Ref. | - | 3374 (2.9) | Ref. | - |
| Q2 | 7726 (2.7) | 1.13 (1.09, 1.17) | <0.001 | 3192 (2.6) | 1.03 (0.96, 1.11) | 0.377 |
| Q3 | 5957 (2.2) | 1.22 (1.18, 1.27) | <0.001 | 2689 (2.3) | 1.09 (1.01, 1.19) | 0.036 |
| Q4 | 5178 (1.9) | 1.44 (1.38, 1.50) | <0.001 | 2254 (1.9) | 1.09 (0.99, 1.20) | 0.097 |
| CI = confidence interval. HR = hazard ratio. Q = quartile. HRs are adjusted for age at conscription, year of conscription, body mass index, parental education, and parental income. In both cohorts, the median (range) of W_max_ in Q1 was 217 (100-236), in Q2 it was 253 (237-270), in Q3 it was 290 (271-312), in Q4 it was 339 (313-999). | | | | | | |
