# Supplementary material for: Adolescent cardiorespiratory fitness and risk of cancer in late adulthood: A nationwide sibling-controlled cohort study in Sweden
Source: PLoS Med. 2025 May 8;22(5):e1004597. doi: 10.1371/journal.pmed.1004597 (PMC12061154; doi:10.1371/journal.pmed.1004597)
Supplement: S6 Table — (DOCX) [file pmed.1004597.s006.docx]

| **S6 Table.** **Standardized cumulative incidence of cancer at 65 years of age by quartiles of cardiorespiratory fitness in cohort and sibling analysis.** | | | | |
| --- | --- | --- | --- | --- |
|  | **Cohort analysis (N=1 124 049)** | | **Sibling analysis (N=477 453)** | |
| **Cancer outcome by quartiles of cardiorespiratory fitness** | **Standardized incidence at age 65 y, % (95% CI)** | **Difference, pp (95% CI)** | **Standardized incidence at age 65 y, % (95% CI)** | **Difference, pp (95% CI)** |
| **Overall cancer diagnosis** | | | | |
| Q1 | 18.04 (17.78, 18.30) | Ref. | 17.81 (17.29, 18.33) | Ref. |
| Q2 | 18.12 (17.87, 18.38) | 0.08 (-0.18, 0.35) | 17.73 (17.28, 18.19) | -0.08 (-0.66, 0.51) |
| Q3 | 18.52 (18.26, 18.79) | 0.48 (0.17, 0.80) | 18.04 (17.58, 18.52) | 0.24 (-0.46, 0.93) |
| Q4 | 19.36 (19.06, 19.67) | 1.32 (0.94, 1.70) | 17.85 (17.30, 18.41) | 0.04 (-0.80, 0.88) |
| **Overall cancer mortality** | | | | |
| Q1 | 3.01 (2.91, 3.11) | Ref. | 2.80 (2.61, 3.00) | Ref. |
| Q2 | 2.50 (2.41, 2.59) | -0.51 (-0.61, -0.40) | 2.48 (2.32, 2.65) | -0.32 (-0.54, -0.10) |
| Q3 | 2.30 (2.21, 2.39) | -0.71 (-0.83, -0.59) | 2.39 (2.22, 2.57) | -0.41 (-0.67, -0.14) |
| Q4 | 2.15 (2.05, 2.25) | -0.85 (-1.00, -0.71) | 2.19 (1.99, 2.41) | -0.61 (-0.93, -0.28) |
| **Site-specific cancers (diagnosis or death)** | | | | |
| Head and neck | | | | |
| Q1 | 0.77 (0.71, 0.82) | Ref. | 0.72 (0.62, 0.84) | Ref. |
| Q2 | 0.65 (0.61, 0.70) | -0.11 (-0.17, -0.06) | 0.67 (0.59, 0.77) | -0.05 (-0.17, 0.08) |
| Q3 | 0.61 (0.57, 0.66) | -0.15 (-0.22, -0.09) | 0.61 (0.52, 0.70) | -0.12 (-0.26, 0.03) |
| Q4 | 0.58 (0.53, 0.63) | -0.19 (-0.26, -0.11) | 0.58 (0.48, 0.69) | -0.14 (-0.31, 0.03) |
| Oesophagus | | | | |
| Q1 | 0.28 (0.24, 0.32) | Ref. | 0.21 (0.16, 0.28) | Ref. |
| Q2 | 0.21 (0.18, 0.24) | -0.07 (-0.10, -0.03) | 0.20 (0.15, 0.26) | -0.01 (-0.08, 0.06) |
| Q3 | 0.17 (0.14, 0.20) | -0.11 (-0.15, -0.07) | 0.14 (0.11, 0.20) | -0.07 (-0.14, 0.00) |
| Q4 | 0.15 (0.12, 0.18) | -0.13 (-0.18, -0.08) | 0.13 (0.09, 0.19) | -0.08 (-0.17, 0.00) |
| Lung | | | | |
| Q1 | 0.63 (0.58, 0.69) | Ref. | 0.58 (0.49, 0.69) | Ref. |
| Q2 | 0.47 (0.43, 0.51) | -0.17 (-0.21, -0.12) | 0.47 (0.40, 0.56) | -0.11 (-0.21, -0.01) |
| Q3 | 0.39 (0.36, 0.43) | -0.24 (-0.30, -0.19) | 0.42 (0.35, 0.51) | -0.16 (-0.28, -0.04) |
| Q4 | 0.31 (0.27, 0.36) | -0.32 (-0.39, -0.26) | 0.29 (0.23, 0.38) | -0.29 (-0.42, -0.15) |
| Stomach | | | | |
| Q1 | 0.27 (0.24, 0.30) | Ref. | 0.25 (0.20, 0.32) | Ref. |
| Q2 | 0.21 (0.19, 0.24) | -0.06 (-0.09, -0.02) | 0.23 (0.18, 0.29) | -0.03 (-0.10, 0.05) |
| Q3 | 0.20 (0.17, 0.23) | -0.07 (-0.11, -0.03) | 0.17 (0.14, 0.22) | -0.08 (-0.15, 0.00) |
| Q4 | 0.19 (0.16, 0.22) | -0.08 (-0.12, -0.04) | 0.23 (0.17, 0.31) | -0.02 (-0.12, 0.08) |
| Pancreas | | | | |
| Q1 | 0.46 (0.42, 0.51) | Ref. | 0.42 (0.34, 0.52) | Ref. |
| Q2 | 0.40 (0.36, 0.44) | -0.07 (-0.11, -0.02) | 0.44 (0.36, 0.54) | 0.02 (-0.08, 0.12) |
| Q3 | 0.39 (0.35, 0.43) | -0.07 (-0.13, -0.02) | 0.41 (0.34, 0.50) | -0.01 (-0.12, 0.11) |
| Q4 | 0.34 (0.29, 0.39) | -0.13 (-0.19, -0.06) | 0.35 (0.27, 0.45) | -0.07 (-0.20, 0.06) |
| Liver, bile ducts, and gallbladder | | | | |
| Q1 | 0.50 (0.45, 0.56) | Ref. | 0.37 (0.30, 0.45) | Ref. |
| Q2 | 0.39 (0.35, 0.43) | -0.12 (-0.16, -0.07) | 0.41 (0.34, 0.49) | 0.04 (-0.06, 0.13) |
| Q3 | 0.30 (0.27, 0.34) | -0.20 (-0.25, -0.15) | 0.36 (0.29, 0.44) | -0.01 (-0.11, 0.09) |
| Q4 | 0.30 (0.26, 0.34) | -0.21 (-0.27, -0.15) | 0.29 (0.22, 0.38) | -0.08 (-0.20, 0.04) |
| Colon | | | | |
| Q1 | 1.16 (1.08, 1.24) | Ref. | 1.09 (0.95, 1.25) | Ref. |
| Q2 | 1.05 (0.98, 1.12) | -0.11 (-0.18, -0.03) | 0.97 (0.86, 1.10) | -0.12 (-0.28, 0.04) |
| Q3 | 0.98 (0.91, 1.05) | -0.18 (-0.27, -0.09) | 1.00 (0.88, 1.15) | -0.09 (-0.28, 0.11) |
| Q4 | 0.87 (0.81, 0.95) | -0.29 (-0.39, -0.19) | 0.86 (0.73, 1.00) | -0.24 (-0.46, -0.01) |
| Rectum | | | | |
| Q1 | 0.86 (0.79, 0.94) | Ref. | 0.86 (0.73, 1.01) | Ref. |
| Q2 | 0.81 (0.75, 0.88) | -0.05 (-0.12, 0.01) | 0.77 (0.66, 0.89) | -0.09 (-0.23, 0.06) |
| Q3 | 0.75 (0.69, 0.82) | -0.11 (-0.18, -0.03) | 0.72 (0.62, 0.84) | -0.14 (-0.30, 0.03) |
| Q4 | 0.69 (0.63, 0.76) | -0.17 (-0.26, -0.08) | 0.59 (0.49, 0.71) | -0.27 (-0.46, -0.08) |
| Kidney | | | | |
| Q1 | 0.64 (0.58, 0.70) | Ref. | 0.51 (0.43, 0.62) | Ref. |
| Q2 | 0.53 (0.49, 0.59) | -0.10 (-0.16, -0.05) | 0.52 (0.44, 0.62) | 0.01 (-0.11, 0.12) |
| Q3 | 0.47 (0.42, 0.52) | -0.17 (-0.23, -0.11) | 0.47 (0.39, 0.56) | -0.05 (-0.17, 0.08) |
| Q4 | 0.41 (0.37, 0.46) | -0.22 (-0.29, -0.15) | 0.44 (0.35, 0.55) | -0.07 (-0.22, 0.08) |
| Prostate | | | | |
| Q1 | 4.93 (4.73, 5.13) | Ref. | 5.03 (4.68, 5.40) | Ref. |
| Q2 | 5.12 (4.92, 5.32) | 0.19 (0.04, 0.34) | 5.04 (4.72, 5.38) | 0.02 (-0.33, 0.36) |
| Q3 | 5.17 (4.96, 5.38) | 0.24 (0.05, 0.43) | 5.15 (4.81, 5.52) | 0.13 (-0.31, 0.56) |
| Q4 | 5.41 (5.16, 5.66) | 0.48 (0.23, 0.73) | 5.07 (4.67, 5.51) | 0.05 (-0.50, 0.60) |
| Bladder | | | | |
| Q1 | 0.66 (0.61, 0.71) | Ref. | 0.59 (0.51, 0.69) | Ref. |
| Q2 | 0.59 (0.54, 0.64) | -0.07 (-0.12, -0.02) | 0.58 (0.50, 0.67) | -0.01 (-0.12, 0.10) |
| Q3 | 0.59 (0.54, 0.64) | -0.07 (-0.13, -0.01) | 0.50 (0.43, 0.59) | -0.09 (-0.21, 0.03) |
| Q4 | 0.49 (0.44, 0.55) | -0.17 (-0.24, -0.10) | 0.52 (0.42, 0.65) | -0.07 (-0.23, 0.09) |
| Myeloma | | | | |
| Q1 | 0.26 (0.23, 0.30) | Ref. | 0.30 (0.22, 0.40) | Ref. |
| Q2 | 0.26 (0.23, 0.29) | 0.00 (-0.04, 0.03) | 0.23 (0.18, 0.29) | -0.07 (-0.16, 0.01) |
| Q3 | 0.26 (0.23, 0.30) | 0.00 (-0.04, 0.04) | 0.25 (0.19, 0.32) | -0.05 (-0.15, 0.05) |
| Q4 | 0.26 (0.23, 0.31) | 0.00 (-0.05, 0.05) | 0.24 (0.18, 0.33) | -0.06 (-0.18, 0.07) |
| Melanoma skin | | | | |
| Q1 | 1.61 (1.53, 1.70) | Ref. | 1.65 (1.48, 1.84) | Ref. |
| Q2 | 1.82 (1.73, 1.91) | 0.21 (0.11, 0.30) | 1.79 (1.63, 1.95) | 0.13 (-0.07, 0.34) |
| Q3 | 2.07 (1.97, 2.17) | 0.45 (0.34, 0.56) | 2.01 (1.85, 2.18) | 0.36 (0.12, 0.60) |
| Q4 | 2.42 (2.31, 2.53) | 0.80 (0.67, 0.94) | 2.13 (1.94, 2.34) | 0.48 (0.19, 0.78) |
| Non-melanoma skin | | | | |
| Q1 | 4.36 (4.24, 4.50) | Ref. | 4.72 (4.44, 5.03) | Ref. |
| Q2 | 4.91 (4.78, 5.05) | 0.55 (0.40, 0.69) | 4.88 (4.63, 5.14) | 0.15 (-0.18, 0.48) |
| Q3 | 5.31 (5.16, 5.46) | 0.94 (0.77, 1.11) | 5.15 (4.89, 5.42) | 0.42 (0.03, 0.81) |
| Q4 | 6.20 (6.02, 6.39) | 1.84 (1.62, 2.05) | 5.12 (4.83, 5.44) | 0.40 (-0.07, 0.87) |
| CI = confidence interval. pp = percentage point. Q = quartile. All estimates are adjusted for age at conscription, year of conscription, body mass index, parental education, and parental income. In both cohorts, the median (range) of W_max_ in Q1 was 217 (100-236), in Q2 it was 253 (237-270), in Q3 it was 290 (271-312), in Q4 it was 339 (313-999). | | | | |
