# Supplementary material for: Adolescent cardiorespiratory fitness and risk of cancer in late adulthood: A nationwide sibling-controlled cohort study in Sweden
Source: PLoS Med. 2025 May 8;22(5):e1004597. doi: 10.1371/journal.pmed.1004597 (PMC12061154; doi:10.1371/journal.pmed.1004597)
Supplement: S7 Table — (DOCX) [file pmed.1004597.s007.docx]

| **S7 Table. Population-attributable fraction for** **cancer at 65 years of age by cardiorespiratory fitness in cohort and sibling analysis, when considering a moderate (shifting those in quartile 1 to quartile 2) or a major intervention (shifting everyone to quartile 4).** | | | | |
| --- | --- | --- | --- | --- |
|  | **Moderate intervention** | | **Major intervention** | |
|  | **Cohort analysis  (N=1 124 049)** | **Sibling analysis  (N=477 453)** | **Cohort analysis  (N=1 124 049)** | **Sibling analysis  (N=477 453)** |
| **Cancer outcome** | **PAF, % (95% CI)** | **PAF, % (95% CI)** | **PAF, % (95% CI)** | **PAF, % (95% CI)** |
| **Overall cancer diagnosis** | -2.7 (-4.1, -1.4) | -0.2 (-3.3, 3.0) | -7.3 (-9.5, -5.2) | -0.2 (-5.0, 4.5) |
| **Overall cancer mortality** | 21.4 (18.8, 24.1) | 14.8 (8.0, 21.6) | 28.4 (24.3, 32.6) | 21.6 (11.3, 32.0) |
| **Site specific cancers (diagnosis or death)** |  |  |  |  |
| Head and neck | 18.4 (12.6, 24.2) | 12.2 (-2.6, 27.0) | 24.5 (16.0, 33.1) | 19.9 (-1.3, 40.1) |
| Oesophagus | 34.2 (25.8, 42.5) | 20.3 (-4.1, 44.6) | 47.3 (39.1, 59.5) | 39.2 (7.7, 70.6) |
| Lung | 35.3 (30.3, 40.3) | 28.8 (15.8, 41.9) | 50.8 (43.3, 58.3) | 49.5 (32.7, 66.4) |
| Stomach | 24.2 (15.3, 33.1) | 15.0 (-7.8, 37.8) | 29.5 (15.7, 43.3) | 9.2 (-28.4, 46.7) |
| Pancreas | 17.9 (10.3, 25.6) | 2.0 (-19.6, 23.6) | 27.3 (15.6, 38.9) | 16.7 (-11.9, 45.3) |
| Liver, bile ducts, and gallbladder | 32.1 (25.8, 38.3) | 1.5 (-20.2, 23.1) | 41.3 (31.6, 51.0) | 21.9 (-6.7, 50.5) |
| Colon | 14.8 (9.5, 20.1) | 13.0 (0.3, 25.6) | 24.8 (17.3, 32.3) | 21.7 (3.6, 39.9) |
| Rectum | 11.0 (4.6, 17.4) | 16.9 (3.0, 30.7) | 19.4 (10.0, 28.7) | 31.0 (12.9, 49.2) |
| Kidney | 23.4 (16.9, 30.0) | 5.0 (-14.4, 24.4) | 35.0 (26.1, 43.9) | 13.9 (-13.1, 40.8) |
| Prostate | -5.6 (-8.6, -2.6) | -1.0 (-7.8, 5.8) | -9.8 (-15.0, -4.6) | -0.9 (-11.9, 10.0) |
| Bladder | 14.2 (7.8, 20.7) | 7.7 (-8.7, 24.1) | 25.5 (15.8, 35.1) | 11.5 (-14.6, 37.6) |
| Myeloma | 0.6 (-11.3, 12.6) | 21.4 (-3.0, 45.7) | 0.3 (-18.3, 18.9) | 19.5 (-18.4, 57.4) |
| Melanoma skin | -25.8 (-32.1, -19.5) | -16.8 (-30.2, -3.4) | -49.7 (-59.7, -39.7) | -29.2 (-49.5, -8.9) |
| Non-melanoma skin | -22.2 (-25.7, -18.7) | -6.0 (-13.0, 1.1) | -42.1 (-47.8, -36.3) | -8.5 (-18.8, 1.9) |
| CI = confidence interval. PAF = population-attributable fraction. All estimates were adjusted for age at conscription, year of conscription, body mass index, parental education, and parental income. Negative estimates imply a theoretically increased risk of the outcome in the population should the relevant intervention be carried out | | | | |
