# Supplementary material for: Adolescent cardiorespiratory fitness and risk of cancer in late adulthood: A nationwide sibling-controlled cohort study in Sweden
Source: PLoS Med. 2025 May 8;22(5):e1004597. doi: 10.1371/journal.pmed.1004597 (PMC12061154; doi:10.1371/journal.pmed.1004597)
Supplement: S9 Table — (DOCX) [file pmed.1004597.s009.docx]

| **S9 Table. Hazard ratios for cancer by quartiles of cardiorespiratory fitness in cohort and sibling analysis, with and without adjusting for body mass index.** | | | | | |  |
| --- | --- | --- | --- | --- | --- | --- |
|  | **Cohort analysis (N=1 124 049)** | | **Sibling analysis (N=477 453)** | | |  |
| **Cancer outcome by quartiles of cardiorespiratory fitness** | **HR (95% CI) without adjustment for BMI** | **HR (95% CI) with  adjustment for BMI** | **HR (95% CI) without adjustment for BMI** | | **HR (95% CI) with adjustment for BMI** |  |
| **Overall cancer diagnosis** |  |  |  |  |  |  |
| Q1 | Ref. | Ref. | Ref. | | Ref. |  |
| Q2 | 1.01 (1.00, 1.03) | 1.01 (0.99, 1.02) | 1.00 (0.96, 1.04) | | 1.00 (0.96, 1.03) |  |
| Q3 | 1.04 (1.02, 1.06) | 1.03 (1.01, 1.05) | 1.02 (0.98, 1.06) | | 1.02 (0.97, 1.06) |  |
| Q4 | 1.10 (1.08, 1.12) | 1.08 (1.06, 1.11) | 1.01 (0.96, 1.06) | | 1.00 (0.95, 1.06) |  |
| **Overall cancer mortality** |  |  |  | |  |  |
| Q1 | Ref. | Ref. | Ref. | | Ref. |  |
| Q2 | 0.87 (0.84, 0.90) | 0.83 (0.80, 0.86) | 0.90 (0.83, 0.98) | | 0.88 (0.81, 0.96) |  |
| Q3 | 0.81 (0.78, 0.85) | 0.76 (0.73, 0.80) | 0.88 (0.79, 0.97) | | 0.85 (0.77, 0.95) |  |
| Q4 | 0.78 (0.74, 0.82) | 0.71 (0.67, 0.76) | 0.82 (0.72, 0.93) | | 0.78 (0.68, 0.89) |  |
| **Site-specific cancer (diagnosis or death)** |  |  |  | |  |  |
| Head and neck |  |  |  | |  |  |
| Q1 | Ref. | Ref. | Ref. | | Ref. |  |
| Q2 | 0.90 (0.83, 0.97) | 0.85 (0.79, 0.93) | 0.93 (0.78, 1.11) | | 0.94 (0.78, 1.12) |  |
| Q3 | 0.86 (0.79, 0.95) | 0.80 (0.73, 0.88) | 0.83 (0.68, 1.03) | | 0.84 (0.68, 1.04) |  |
| Q4 | 0.84 (0.75, 0.93) | 0.75 (0.67, 0.85) | 0.79 (0.61, 1.03) | | 0.80 (0.62, 1.04) |  |
| Oesophagus |  |  |  | |  |  |
| Q1 | Ref. | Ref. | Ref. | | Ref. |  |
| Q2 | 0.83 (0.73, 0.95) | 0.75 (0.65, 0.86) | 0.99 (0.72, 1.37) | | 0.95 (0.69, 1.32) |  |
| Q3 | 0.69 (0.58, 0.82) | 0.60 (0.50, 0.72) | 0.72 (0.48, 1.08) | | 0.68 (0.45, 1.03) |  |
| Q4 | 0.63 (0.51, 0.79) | 0.53 (0.42, 0.66) | 0.66 (0.39, 1.09) | | 0.61 (0.36, 1.02) |  |
| Lung |  |  |  | |  |  |
| Q1 | Ref. | Ref. | Ref. | | Ref. |  |
| Q2 | 0.74 (0.68, 0.81) | 0.74 (0.68, 0.80) | 0.79 (0.65, 0.95) | | 0.81 (0.67, 0.98) |  |
| Q3 | 0.62 (0.56, 0.70) | 0.62 (0.55, 0.69) | 0.70 (0.55, 0.89) | | 0.73 (0.56, 0.93) |  |
| Q4 | 0.50 (0.43, 0.58) | 0.49 (0.42, 0.57) | 0.48 (0.34, 0.66) | | 0.50 (0.36, 0.70) |  |
| Stomach |  |  |  | |  |  |
| Q1 | Ref. | Ref. | Ref. | | Ref. |  |
| Q2 | 0.87 (0.76, 0.99) | 0.79 (0.70, 0.90) | 0.94 (0.70, 1.25) | | 0.90 (0.67, 1.21) |  |
| Q3 | 0.83 (0.71, 0.97) | 0.74 (0.63, 0.86) | 0.73 (0.52, 1.02) | | 0.69 (0.49, 0.98) |  |
| Q4 | 0.83 (0.69, 1.00) | 0.71 (0.58, 0.86) | 0.98 (0.65, 1.47) | | 0.90 (0.60, 1.37) |  |
| Pancreas |  |  |  | |  |  |
| Q1 | Ref. | Ref. | Ref. | | Ref. |  |
| Q2 | 0.92 (0.83, 1.02) | 0.86 (0.78, 0.95) | 1.11 (0.88, 1.41) | | 1.05 (0.83, 1.33) |  |
| Q3 | 0.92 (0.81, 1.04) | 0.84 (0.74, 0.95) | 1.07 (0.81, 1.41) | | 0.98 (0.74, 1.30) |  |
| Q4 | 0.82 (0.70, 0.96) | 0.73 (0.62, 0.85) | 0.93 (0.66, 1.30) | | 0.83 (0.59, 1.18) |  |
| Liver, bile ducts, and gallbladder |  |  |  | |  |  |
| Q1 | Ref. | Ref. | Ref. | | Ref. |  |
| Q2 | 0.83 (0.76, 0.92) | 0.77 (0.69, 0.85) | 1.16 (0.92, 1.47) | | 1.10 (0.86, 1.39) |  |
| Q3 | 0.67 (0.59, 0.76) | 0.60 (0.52, 0.68) | 1.06 (0.80, 1.39) | | 0.97 (0.73, 1.28) |  |
| Q4 | 0.68 (0.58, 0.80) | 0.59 (0.50, 0.69) | 0.87 (0.61, 1.25) | | 0.78 (0.54, 1.13) |  |
| Colon |  |  |  | |  |  |
| Q1 | Ref. | Ref. | Ref. | | Ref. |  |
| Q2 | 0.93 (0.87, 1.00) | 0.91 (0.85, 0.97) | 0.90 (0.78, 1.05) | | 0.89 (0.76, 1.04) |  |
| Q3 | 0.88 (0.81, 0.95) | 0.84 (0.78, 0.91) | 0.94 (0.78, 1.13) | | 0.92 (0.76, 1.11) |  |
| Q4 | 0.80 (0.72, 0.88) | 0.75 (0.68, 0.83) | 0.81 (0.64, 1.01) | | 0.78 (0.62, 0.99) |  |
| Rectum |  |  |  | |  |  |
| Q1 | Ref. | Ref. | Ref. | | Ref. |  |
| Q2 | 0.96 (0.89, 1.04) | 0.94 (0.87, 1.02) | 0.94 (0.78, 1.12) | | 0.90 (0.75, 1.07) |  |
| Q3 | 0.90 (0.82, 0.99) | 0.88 (0.80, 0.96) | 0.90 (0.73, 1.10) | | 0.84 (0.68, 1.04) |  |
| Q4 | 0.84 (0.75, 0.94) | 0.81 (0.72, 0.91) | 0.75 (0.58, 0.97) | | 0.69 (0.53, 0.90) |  |
| Kidney |  |  |  | |  |  |
| Q1 | Ref. | Ref. | Ref. | | Ref. |  |
| Q2 | 0.93 (0.85, 1.02) | 0.84 (0.76, 0.92) | 1.06 (0.86, 1.32) | | 1.01 (0.81, 1.26) |  |
| Q3 | 0.85 (0.76, 0.95) | 0.73 (0.65, 0.82) | 0.98 (0.76, 1.25) | | 0.91 (0.70, 1.18) |  |
| Q4 | 0.79 (0.69, 0.91) | 0.65 (0.57, 0.75) | 0.95 (0.70, 1.29) | | 0.86 (0.63, 1.18) |  |
| Prostate |  |  |  | |  |  |
| Q1 | Ref. | Ref. | Ref. | | Ref. |  |
| Q2 | 1.04 (1.01, 1.07) | 1.04 (1.01, 1.07) | 1.00 (0.93, 1.07) | | 1.00 (0.94, 1.08) |  |
| Q3 | 1.05 (1.01, 1.09) | 1.05 (1.01, 1.09) | 1.02 (0.94, 1.11) | | 1.03 (0.94, 1.12) |  |
| Q4 | 1.10 (1.05, 1.15) | 1.10 (1.05, 1.16) | 1.00 (0.90, 1.12) | | 1.01 (0.90, 1.13) |  |
| Bladder |  |  |  | |  |  |
| Q1 | Ref. | Ref. | Ref. | | Ref. |  |
| Q2 | 0.92 (0.85, 1.00) | 0.90 (0.82, 0.97) | 0.99 (0.82, 1.20) | | 0.98 (0.81, 1.19) |  |
| Q3 | 0.93 (0.84, 1.02) | 0.89 (0.81, 0.99) | 0.86 (0.69, 1.07) | | 0.85 (0.68, 1.05) |  |
| Q4 | 0.78 (0.69, 0.89) | 0.75 (0.65, 0.85) | 0.91 (0.68, 1.21) | | 0.89 (0.66, 1.19) |  |
| Myeloma |  |  |  | |  |  |
| Q1 | Ref. | Ref. | Ref. | | Ref. |  |
| Q2 | 1.04 (0.90, 1.18) | 0.99 (0.86, 1.13) | 0.78 (0.57, 1.07) | | 0.76 (0.55, 1.04) |  |
| Q3 | 1.07 (0.92, 1.25) | 1.00 (0.86, 1.17) | 0.87 (0.61, 1.26) | | 0.83 (0.57, 1.20) |  |
| Q4 | 1.09 (0.91, 1.31) | 1.00 (0.83, 1.20) | 0.86 (0.55, 1.36) | | 0.81 (0.50, 1.29) |  |
| Melanoma skin |  |  |  | |  |  |
| Q1 | Ref. | Ref. | Ref. | | Ref. |  |
| Q2 | 1.17 (1.10, 1.23) | 1.13 (1.07, 1.19) | 1.11 (0.98, 1.25) | | 1.08 (0.96, 1.22) |  |
| Q3 | 1.34 (1.27, 1.43) | 1.28 (1.21, 1.37) | 1.26 (1.11, 1.44) | | 1.22 (1.06, 1.40) |  |
| Q4 | 1.60 (1.50, 1.71) | 1.50 (1.41, 1.61) | 1.36 (1.17, 1.59) | | 1.30 (1.11, 1.52) |  |
| Non-melanoma skin |  |  |  | |  |  |
| Q1 | Ref. | Ref. | Ref. | | Ref. |  |
| Q2 | 1.10 (1.06, 1.13) | 1.13 (1.09, 1.17) | 1.01 (0.95, 1.09) | | 1.03 (0.96, 1.11) |  |
| Q3 | 1.17 (1.13, 1.21) | 1.22 (1.18, 1.27) | 1.06 (0.98, 1.15) | | 1.09 (1.01, 1.19) |  |
| Q4 | 1.35 (1.30, 1.40) | 1.44 (1.37, 1.50) | 1.05 (0.96, 1.15) | | 1.09 (0.99, 1.20) |  |
| BMI = body mass index. CI = confidence interval. HR = hazard ratio. Q = quartile. All estimates were adjusted for birth cohort, year of conscription, parental education, and parental income. In both cohorts, the median (range) of W_max_ in Q1 was 217 (100-236), in Q2 it was 253 (237-270), in Q3 it was 290 (271-312), in Q4 it was 339 (313-999). | | | | | | |
