# Supplementary material for: Adolescent cardiorespiratory fitness and risk of cancer in late adulthood: A nationwide sibling-controlled cohort study in Sweden
Source: PLoS Med. 2025 May 8;22(5):e1004597. doi: 10.1371/journal.pmed.1004597 (PMC12061154; doi:10.1371/journal.pmed.1004597)
Supplement: S11 Table — (DOCX) [file pmed.1004597.s011.docx]

| **S11 Table. Hazard ratios for overall cancer diagnosis and mortality by quartiles of cardiorespiratory fitness in cohort analysis and sibling analysis using Cox regression compared to using Flexible parametric regression.** | | | | | | | | | | | | |
| --- | --- | --- | --- | --- | --- | --- | --- | --- | --- | --- | --- | --- |
| **Overall cancer diagnosis** | | | | | | | | | | | | |
| **Cohort analysis (N=1 124 049)** | | | | | |  | **Sibling analysis (N=477 453)** | | | | | |
|  | **Flexible parametric model** | |  | **Cox regression** | |  |  | **Flexible parametric model** | |  | **Cox regression** | |
| **Quartiles of fitness** | **HR** | **95% CI** |  | **HR** | **95% CI** |  | **Quartiles of fitness** | **HR** | **95% CI** |  | **HR** | **95% CI** |
| Q1 | 1.000 | - |  | 1.000 | - |  | Q1 | 1.000 | - |  | 1.000 | - |
| Q2 | 1.005 | 0.99, 1.02 |  | 1.005 | 0.989, 1.021 |  | Q2 | 0.995 | 0.960, 1.032 |  | 0.995 | 0.959, 1.032 |
| Q3 | 1.030 | 1.01, 1.05 |  | 1.029 | 1.010, 1.049 |  | Q3 | 1.015 | 0.972, 1.059 |  | 1.013 | 0.971, 1.058 |
| Q4 | 1.082 | 1.06, 1.11 |  | 1.081 | 1.057, 1.105 |  | Q4 | 1.003 | 0.952, 1.056 |  | 1.000 | 0.950, 1.054 |
| **Overall cancer mortality** | | | | | | | | | | | | |
| **Cohort analysis (N=1 124 049)** | | | | | | | **Sibling analysis (N=477 453)** | | | | | |
|  | **Flexible parametric model** | |  | **Cox regression** | |  |  | **Flexible parametric model** | |  | **Cox regression** | |
| **Quartiles of fitness** | **HR** | **95% CI** |  | **HR** | **95% CI** |  | **Quartiles of fitness** | **HR** | **95% CI** |  | **HR** | **95% CI** |
| Q1 | 1.000 | - |  | 1.000 | - |  | Q1 | 1.000 | - |  | 1.000 | - |
| Q2 | 0.829 | 0.798, 0.861 |  | 0.829 | 0.798, 0.861 |  | Q2 | 0.884 | 0.812, 0.963 |  | 0.884 | 0.812, 0.963 |
| Q3 | 0.762 | 0.728, 0.798 |  | 0.762 | 0.728, 0.798 |  | Q3 | 0.852 | 0.768, 0.945 |  | 0.852 | 0.768, 0.945 |
| Q4 | 0.712 | 0.672, 0.755 |  | 0.712 | 0.672, 0.755 |  | Q4 | 0.781 | 0.684, 0.893 |  | 0.781 | 0.683, 0.893 |
| CI = confidence interval. HR = hazard ratio. Q = quartile. All estimates are adjusted for age at conscription, year of conscription, body mass index, parental education, and parental income. In both cohorts, the median (range) of W_max_ in Q1 was 217 (100-236), in Q2 it was 253 (237-270), in Q3 it was 290 (271-312), in Q4 it was 339 (313-999). | | | | | | | | | | | | |
