# Supplementary material for: Adolescent cardiorespiratory fitness and risk of cancer in late adulthood: A nationwide sibling-controlled cohort study in Sweden
Source: PLoS Med. 2025 May 8;22(5):e1004597. doi: 10.1371/journal.pmed.1004597 (PMC12061154; doi:10.1371/journal.pmed.1004597)
Supplement: S12 Table — (DOCX) [file pmed.1004597.s012.docx]

| **S12 Table**. **Hazard ratios for overall cancer diagnosis and mortality by quartiles of cardiorespiratory fitness with and without allowing for effect modification by BMI in cohort and sibling analysis.** | | | | | |
| --- | --- | --- | --- | --- | --- |
|  | **Fully adjusted model assuming  no effect modification by BMI, as reported in the main article^a^** | |  | **Fully adjusted model including interaction terms between fitness and BMI^b^** | |
|  | **Cohort analysis  (N=1 124 049)** | **Sibling analysis  (N=477 453)** |  | **Cohort analysis  (N=1 124 049)** | **Sibling analysis  (N=477 453)** |
| **Quartiles of fitness** | **HR (95% CI)** | **HR (95% CI)** | **Quartiles of fitness** | **HR (95% CI)** | **HR (95% CI)** |
| **Total population** | **Overall cancer diagnosis** | | **Total population** | **Overall cancer diagnosis** | |
| Q1 | Ref. | Ref. | Q1 | Ref. | Ref. |
| Q2 | 1.01 (0.99, 1.02) | 1.00 (0.96, 1.03) | Q2 | 1.01 (0.99, 1.03) | 1.00 (0.96, 1.04) |
| Q3 | 1.03 (1.01, 1.05) | 1.02 (0.97, 1.06) | Q3 | 1.04 (1.02, 1.06) | 1.03 (0.98, 1.08) |
| Q4 | 1.08 (1.06, 1.11) | 1.00 (0.95, 1.06) | Q4 | 1.10 (1.08, 1.13) | 1.02 (0.97, 1.07) |
|  |  |  | **Underweight** |  |  |
|  |  |  | Q1 | Ref. | Ref. |
|  |  |  | Q2 | 1.04 (0.98, 1.10) | 1.00 (0.89, 1.12) |
|  |  |  | Q3 | 1.01 (0.93, 1.10) | 1.13 (0.96, 1.34) |
|  |  |  | Q4 | 0.99 (0.86, 1.14) | 1.10 (0.93, 1.46) |
|  |  |  | **Normal weight** |  |  |
|  |  |  | Q1 | Ref. | Ref. |
|  |  |  | Q2 | 1.01 (0.99, 1.03) | 1.00 (0.96, 1.04) |
|  |  |  | Q3 | 1.04 (1.02, 1.06) | 1.03 (0.98, 1.08) |
|  |  |  | Q4 | 1.10 (1.08, 1.13) | 1.02 (0.97, 1.07) |
|  |  |  | **Overweight** |  |  |
|  |  |  | Q1 | Ref. | Ref. |
|  |  |  | Q2 | 0.99 (0.93, 1.05) | 1.01 (0.89, 1.15) |
|  |  |  | Q3 | 0.99 (0.93, 1.05) | 0.92 (0.80, 1.05) |
|  |  |  | Q4 | 1.02 (0.95, 1.09) | 0.94 (0.81, 1.08) |
|  |  |  | **Obesity** |  |  |
|  |  |  | Q1 | Ref. | Ref. |
|  |  |  | Q2 | 1.01 (0.88, 1.15) | 0.95 (0.72, 1.27) |
|  |  |  | Q3 | 0.97 (0.84, 1.12) | 0.94 (0.69, 1.28) |
|  |  |  | Q4 | 1.03 (0.89, 1.20) | 0.89 (0.64, 1.23) |
| **Total population** | **Overall cancer mortality** | | **Total population** | **Overall cancer mortality** | |
| Q1 | Ref. | Ref. | Q1 | Ref. | Ref. |
| Q2 | 0.83 (0.80, 0.86) | 0.88 (0.81, 0.96) | Q2 | 0.85 (0.81, 0.88) | 0.90 (0.82, 0.98) |
| Q3 | 0.76 (0.73, 0.80) | 0.85 (0.77, 0.95) | Q3 | 0.80 (0.76, 0.84) | 0.86 (0.77, 0.96) |
| Q4 | 0.71 (0.67, 0.76) | 0.78 (0.68, 0.89) | Q4 | 0.76 (0.72, 0.81) | 0.79 (0.69, 0.91) |
|  |  |  | **Underweight** |  |  |
|  |  |  | Q1 | Ref. | Ref. |
|  |  |  | Q2 | 0.79 (0.68, 0.91) | 0.89 (0.66, 1.20) |
|  |  |  | Q3 | 0.71 (0.56, 0.89) | 1.05 (0.66, 1.66) |
|  |  |  | Q4 | 0.76 (0.50, 1.13) | 1.09 (0.47, 2.50) |
|  |  |  | **Normal weight** |  |  |
|  |  |  | Q1 | Ref. | Ref. |
|  |  |  | Q2 | 0.85 (0.81, 0.88) | 0.90 (0.82, 0.98) |
|  |  |  | Q3 | 0.80 (0.76, 0.84) | 0.86 (0.77, 0.96) |
|  |  |  | Q4 | 0.76 (0.72, 0.80) | 0.79 (0.69, 0.91) |
|  |  |  | **Overweight** |  |  |
|  |  |  | Q1 | Ref. | Ref. |
|  |  |  | Q2 | 0.93 (0.83, 1.05) | 0.94 (0.71, 1.24) |
|  |  |  | Q3 | 0.80 (0.70, 0.92) | 0.99 (0.74, 1.34) |
|  |  |  | Q4 | 0.73 (0.63, 0.85) | 0.91 (0.64, 1.27) |
|  |  |  | **Obesity** |  |  |
|  |  |  | Q1 | Ref. | Ref. |
|  |  |  | Q2 | 0.90 (0.71, 1.14) | 0.85 (0.47, 1.57) |
|  |  |  | Q3 | 0.89 (0.68, 1.17) | 0.78 (0.41, 1.49) |
|  |  |  | Q4 | 0.85 (0.62, 1.16) | 0.87 (0.42, 1.79) |
| BMI = body mass index. CI = confidence interval. HR = hazard ratio. Q = quartile. Estimates are obtained using flexible parametric survival models, extended to a marginalized between-within model in the sibling cohort.  ^a^Adjusted for age at conscription, year of conscription, BMI, parental education, and parental income. ^b^Adjusted for age at conscription, year of conscription, BMI, parental education, parental income, (and additionally for interaction terms between fitness and BMI categories in the total population).  In both cohorts, the median (range) of W_max_ in Q1 was 217 (100-236), in Q2 it was 253 (237-270), in Q3 it was 290 (271-312), in Q4 it was 339 (313-999). | | | | | |
