# Supplementary material for: Adolescent cardiorespiratory fitness and risk of cancer in late adulthood: A nationwide sibling-controlled cohort study in Sweden
Source: PLoS Med. 2025 May 8;22(5):e1004597. doi: 10.1371/journal.pmed.1004597 (PMC12061154; doi:10.1371/journal.pmed.1004597)
Supplement: S13 Table — (DOCX) [file pmed.1004597.s013.docx]

| **S13 Table. Hazard ratios for overall cancer diagnosis and mortality by quartiles of cardiorespiratory fitness in cohort and sibling analysis using complete-case analysis versus using multiple imputation.** | | | | | | | | | | | | |  |
| --- | --- | --- | --- | --- | --- | --- | --- | --- | --- | --- | --- | --- | --- |
| **Overall cancer diagnosis** | | | | | | | | | | | | |  |
| **Cohort anaysis** | | | | | | **Sibling analysis** | | | | | | |  |
|  | **Complete-case analysis (N=1 124 049)** | |  | **Multiple imputation using chained equations  (N=1 216 782, K=20)** | | |  | **Complete-case analysis  (N=477 453)** | |  | **Multiple imputation using chained equations  (N=506 096, K=20)** | | |
| **Quartiles of fitness** | **HR** | **95% CI** |  | **HR** | **95% CI** | | **Quartiles of fitness** | **HR** | **95% CI** |  | **HR** | **95% CI** | |
| Q1 | 1.00 | - |  | 1.00 | - | | Q1 | 1.00 | - |  | 1.00 | - | |
| Q2 | 1.01 | 0.99, 1.02 |  | 1.00 | 0.99, 1.02 | | Q2 | 1.00 | 0.96, 1.03 |  | 0.99 | 0.96, 1.03 | |
| Q3 | 1.03 | 1.01, 1.05 |  | 1.03 | 1.01, 1.04 | | Q3 | 1.02 | 0.97, 1.06 |  | 1.01 | 0.97, 1.05 | |
| Q4 | 1.08 | 1.06, 1.11 |  | 1.08 | 1.06, 1.10 | | Q4 | 1.00 | 0.95, 1.06 |  | 1.00 | 0.95, 1.05 | |
| **Overall cancer mortality** | | | | | | | | | | | | |  |
| **Cohort analysis** | | | | | | **Sibling analysis** | | | | | | |  |
|  | **Complete-case analysis (N=1 124 049)** | |  | **Multiple imputation using chained equations  (N=1 216 782, K=20)** | | |  | **Complete-case analysis  (N=477 453)** | |  | **Multiple imputation using chained equations  (N=506 096, K=20)** | | |
| **Quartiles of fitness** | **HR** | **95% CI** |  | **HR** | **95% CI** | | **Quartiles of fitness** | **HR** | **95% CI** |  | **HR** | **95% CI** | |
| Q1 | 1.00 | - |  | 1.00 | - | | Q1 | 1.00 | - |  | 1.00 | - | |
| Q2 | 0.83 | 0.80, 0.86 |  | 0.84 | 0.81, 0.87 | | Q2 | 0.88 | 0.81, 0.96 |  | 0.88 | 0.81, 0.95 | |
| Q3 | 0.76 | 0.73, 0.80 |  | 0.75 | 0.72, 0.79 | | Q3 | 0.85 | 0.77, 0.95 |  | 0.84 | 0.76, 0.92 | |
| Q4 | 0.71 | 0.67, 0.76 |  | 0.71 | 0.67, 0.75 | | Q4 | 0.78 | 0.68, 0.89 |  | 0.78 | 0.69, 0.89 | |
| CI = confidence interval. HR = hazard ratio. Q = quartile. ^a^The procedure was performed separately for the total cohort and full siblings (using K=20 repetitions), using linear and multinomial logistic models for continuous (BMI) and categorical parameters (quartiles of cardiorespiratory fitness, parental education, and income). Age at conscription, conscription year, follow-up time, and censoring were used as complete auxiliaries, while weight and length at conscription were treated as partially observed auxiliaries (imputed using a linear model). | | | | | | | | | | | | |  |
