# Supplementary material for: Adolescent cardiorespiratory fitness and risk of cancer in late adulthood: A nationwide sibling-controlled cohort study in Sweden
Source: PLoS Med. 2025 May 8;22(5):e1004597. doi: 10.1371/journal.pmed.1004597 (PMC12061154; doi:10.1371/journal.pmed.1004597)
Supplement: S14 Table — (DOCX) [file pmed.1004597.s014.docx]

| **S14 Table. Hazard ratios for overall cancer diagnosis and mortality by quartiles of cardiorespiratory fitness in cohort and sibling analysis using various numbers of knots for the baseline hazard based on Harrell’s recommendations^a^.** | | | | | | | | | | | | | | | | | | | | | | | | | | | | | | | | | | | | | | |
| --- | --- | --- | --- | --- | --- | --- | --- | --- | --- | --- | --- | --- | --- | --- | --- | --- | --- | --- | --- | --- | --- | --- | --- | --- | --- | --- | --- | --- | --- | --- | --- | --- | --- | --- | --- | --- | --- | --- |
| **Overall cancer diagnosis** | | | | | | | | | | | | | | | | | | | | | | | | | | | | | | | | | | | | | | |
| **Cohort analysis (N=1 124 049)** | | | | | | | | | | | | | | | | | | | | | | | | | | | | | | | | | | | | | | |
|  | **3 knots** | | |  | | **4 knots** | | |  | | | **5 knots** | | | | |  | **6 knots** | | | | | | | | | |  | | | | | **7 knots** | | | | | |
| **Quartiles of fitness** | **HR** | **95% CI** |  | | **HR** | | **95% CI** | |  | | | **HR** | | | **95% CI** | |  | **HR** | | | **95% CI** | | | | | | |  | | | | | **HR** | | **95% CI** | | | |
| Q1 | 1.000 | - |  | | 1.000 | | - | |  | | | 1.000 | | | - | |  | 1.000 | | | - | | | | | | |  | | | | | 1.000 | | - | | | |
| Q2 | 1.005 | 0.989, 1.022 |  | | 1.005 | | 0.989, 1.022 | |  | | | 1.005 | | | 0.989, 1.022 | |  | 1.005 | | | 0.989, 1.022 | | | | | | |  | | | | | 1.005 | | 0.989, 1.022 | | | |
| Q3 | 1.030 | 1.010, 1.049 |  | | 1.029 | | 1.010, 1.049 | |  | | | 1.030 | | | 1.011, 1.050 | |  | 1.030 | | | 1.010, 1.050 | | | | | | |  | | | | | 1.029 | | 1.010, 1.049 | | | |
| Q4 | 1.080 | 1.056, 1.105 |  | | 1.080 | | 1.056, 1.104 | |  | | | 1.082 | | | 1.058, 1.106 | |  | 1.082 | | | 1.058, 1.106 | | | | | | |  | | | | | 1.081 | | 1.057, 1.105 | | | |
| **Sibling analysis (N=477 453)** | | | | | | | | | | | | | | | | | | | | | | | | | | | | | | | | | | | | | | |
|  | **3 knots** | | |  | | **4 knots** | |  | | **5 knots** | | | | | | |  | **6 knots** | | | | | | | | |  | | | | | | | **7 knots** | | | | |
|  | **HR** | **95% CI** |  | | **HR** | | **95% CI** |  | | **HR** | | | | | **95% CI** | |  | **HR** | | **95% CI** | | | | | | |  | | | | | | | **HR** | **95% CI** | | | |
| Q1 | 1.000 | - |  | | 1.000 | | - |  | | 1.000 | | | | | - | |  | 1.000 | | - | | | | | | |  | | | | | | | 1.000 | - | | | |
| Q2 | 0.995 | 0.960, 1.032 |  | | 0.995 | | 0.959, 1.032 |  | | 0.995 | | | | | 0.960, 1.032 | |  | 0.995 | | 0.959, 1.032 | | | | | | |  | | | | | | | 0.995 | 0.959, 1.032 | | | |
| Q3 | 1.014 | 0.971, 1.058 |  | | 1.013 | | 0.971, 1.058 |  | | 1.015 | | | | | 0.972, 1.059 | |  | 1.015 | | 0.972, 1.059 | | | | | | |  | | | | | | | 1.014 | 0.971, 1.058 | | | |
| Q4 | 1.000 | 0.950, 1.054 |  | | 1.000 | | 0.949, 1.054 |  | | 1.003 | | | | | 0.952, 1.056 | |  | 1.002 | | 0.951, 1.056 | | | | | | |  | | | | | | | 1.001 | 0.950, 1.055 | | | |
| **Overall cancer diagnosis** | | | | | | | | | | | | | | | | | | | | | | | | | | | | | | | | | | | | | | |
| **Cohort analysis (N=1 124 049)** | | | | | | | | | | | | | | | | | | | | | | | | | | | | | | | | | | | | | | |
|  | **3 knots** | | |  | | **4 knots** | |  | | **5 knots** | | | | | | |  | | **6 knots** | | | | | |  | | | | | **7 knots** | | | | | | | | |
| **Quartiles of fitness** | **HR** | **95% CI** |  | | **HR** | | **95% CI** | |  | | | **HR** | | **95% CI** | | |  | | **HR** | | | **95% CI** | | |  | | | | **HR** | | | | | | | **95% CI** | | |
| Q1 | 1.000 | - |  | | 1.000 | | - | |  | | | 1.000 | | - | | |  | | 1.000 | | | - | | |  | | | | 1.000 | | | | | | | - | | |
| Q2 | 0.829 | 0.798, 0.861 |  | | 0.829 | | 0.798, 0.861 | |  | | | 0.829 | | 0.798, 0.861 | | |  | | 0.829 | | | 0.798, 0.861 | | |  | | | | 0.829 | | | | | | | 0.798, 0.861 | | |
| Q3 | 0.763 | 0.728, 0.799 |  | | 0.762 | | 0.728, 0.798 | |  | | | 0.762 | | 0.728, 0.798 | | |  | | 0.762 | | | 0.728, 0.799 | | |  | | | | 0.762 | | | | | | | 0.728, 0.798 | | |
| Q4 | 0.713 | 0.672, 0.755 |  | | 0.712 | | 0.672, 0.755 | |  | | | 0.712 | | 0.672, 0.755 | | |  | | 0.713 | | | 0.672, 0.755 | | |  | | | | 0.712 | | | | | | | 0.672, 0.755 | | |
| **Sibling analysis (N=477 453)** | | | | | | | | | | | | | | | | | | | | | | | | | | | | | | | | | | | | | | |
|  | **3 knots** | | |  | | **4 knots** | |  | | **5 knots (main analysis)** | | | | | |  | | **6 knots** | | | | | |  | | | | | | | **7 knots** | | | | | | | |
| **Quartiles of fitness** | **HR** | **95% CI** |  | | | **HR** | **95% CI** | |  | | **HR** | | **95% CI** | | |  | | **HR** | | | | | **95% CI** | | |  | | | | | | **HR** | | | | | **95% CI** |  |
| Q1 | 1.000 | - |  | | | 1.000 | - | |  | | 1.000 | | - | | |  | | 1.000 | | | | | - | | |  | | | | | | 1.000 | | | | | - |  |
| Q2 | 0.884 | 0.812, 0.963 |  | | | 0.884 | 0.812, 0.963 | |  | | 0.884 | | 0.812, 0.963 | | |  | | 0.884 | | | | | 0.812, 0.963 | | |  | | | | | | 0.884 | | | | | 0.812, 0.963 |  |
| Q3 | 0.853 | 0.769, 0.946 |  | | | 0.852 | 0.768, 0.945 | |  | | 0.852 | | 0.768, 0.945 | | |  | | 0.852 | | | | | 0.768, 0.945 | | |  | | | | | | 0.852 | | | | | 0.768, 0.945 |  |
| Q4 | 0.782 | 0.684, 0.894 |  | | | 0.782 | 0.684, 0.893 | |  | | 0.781 | | 0.684, 0.893 | | |  | | 0.781 | | | | | 0.684, 0.893 | | |  | | | | | | 0.781 | | | | | 0.684, 0.893 |  |
| CI = confidence interval. HR = hazard ratio. Q = quartile. All estimates are adjusted for age at conscription, year of conscription, body mass index, parental education, and parental income.  ^a^The placement of knots on the percentile of uncensored log survival times according to Harrell’s recommendations are as follows: for 3 knots: 10p, 50p, 90p; for 4 knots: 5p, 35p, 65p, 95p; for 5 knots: 5p, 27.5p, 50p, 72.5p, 95p; for 6 knots: 5p, 23p, 41p, 59p, 77p, 95p; and for 7 knots: 2.5p, 18.33p, 34.17p, 50p, 65.83p, 81.67p, 97.5p. | | | | | | | | | | | | | | | | | | | | | | | | | | | | | | | | | | | | | | |
