# Supplementary material for: Adolescent cardiorespiratory fitness and risk of cancer in late adulthood: A nationwide sibling-controlled cohort study in Sweden
Source: PLoS Med. 2025 May 8;22(5):e1004597. doi: 10.1371/journal.pmed.1004597 (PMC12061154; doi:10.1371/journal.pmed.1004597)
Supplement: S16 Table — (DOCX) [file pmed.1004597.s016.docx]

| **S16 Table. Time-specific^a^ hazard ratios for cancer by quartiles of cardiorespiratory fitness in cohort and sibling analysis.** | | | | | | | | | | | | | | | |
| --- | --- | --- | --- | --- | --- | --- | --- | --- | --- | --- | --- | --- | --- | --- | --- |
|  | **Cohort analysis (N=1 124 049)** | | | | | | | | **Sibling analysis (N=477 453)** | | | | | | |
|  | **Quartile 1** | **Quartile 2** | | | **Quartile 3** | | **Quartile 4** | | **Quartile 1** | **Quartile 2** | | **Quartile 3** | | **Quartile 4** | |
| **Overall cancer diagnosis** | | |  |  |  |  |  |  |  |  |  |  |  |  |  |
| **Age** | **Ref.** | **HR** | | **95% CI** | **HR** | **95% CI** | **HR** | **95% CI** | **Ref.** | **HR** | **95% CI** | **HR** | **95% CI** | **HR** | **95% CI** |
| 30 | 1.00 | 1.61 | | 1.45‐1.79 | 2.37 | 1.45, 1.79 | 2.85 | 2.59, 3.14 | 1.00 | 1.15 | 0.94, 1.42 | 1.36 | 1.11, 1.66 | 1.41 | 1.13, 1.76 |
| 35 | 1.00 | 1.32 | | 1.24, 1.40 | 1.71 | 1.24, 1.4 | 1.92 | 1.81, 2.03 | 1.00 | 1.11 | 0.98, 1.25 | 1.21 | 1.07, 1.37 | 1.24 | 1.08, 1.42 |
| 40 | 1.00 | 1.12 | | 1.08, 1.17 | 1.30 | 1.08, 1.17 | 1.39 | 1.33, 1.45 | 1.00 | 1.07 | 0.97, 1.17 | 1.10 | 0.99, 1.21 | 1.11 | 0.99, 1.24 |
| 45 | 1.00 | 1.01 | | 0.98, 1.05 | 1.07 | 0.98, 1.05 | 1.11 | 1.06, 1.15 | 1.00 | 1.03 | 0.94, 1.12 | 1.03 | 0.94, 1.12 | 1.02 | 0.93, 1.12 |
| 50 | 1.00 | 0.97 | | 0.95, 1.00 | 0.96 | 0.95, 1.00 | 0.98 | 0.95, 1.01 | 1.00 | 0.98 | 0.93, 1.04 | 0.99 | 0.93, 1.05 | 0.97 | 0.90, 1.04 |
| 55 | 1.00 | 0.98 | | 0.96, 1.01 | 0.94 | 0.96, 1.01 | 0.96 | 0.93, 1.00 | 1.00 | 0.96 | 0.90, 1.02 | 0.98 | 0.92, 1.05 | 0.95 | 0.88, 1.04 |
| 60 | 1.00 | 0.99 | | 0.98, 1.01 | 0.98 | 0.98, 1.01 | 0.99 | 0.96, 1.02 | 1.00 | 0.99 | 0.94, 1.03 | 1.00 | 0.95, 1.05 | 0.97 | 0.90, 1.04 |
| 65 | 1.00 | 1.00 | | 0.97, 1.03 | 1.03 | 0.97, 1.03 | 1.02 | 0.96, 1.08 | 1.00 | 1.01 | 0.95, 1.09 | 1.01 | 0.93, 1.11 | 0.98 | 0.86, 1.11 |
| **Overall cancer mortality** | | | | | | | | | | | | | | | |
| **Age** | **Ref.** | **HR** | | **95% CI** | **HR** | **95% CI** | **HR** | **95% CI** | **Ref.** | **HR** | **95% CI** | **HR** | **95% CI** | **HR** | **95% CI** |
| 30 | 1.00 | 0.93 | | 0.83, 1.04 | 0.90 | 0.83, 1.04 | 0.97 | 0.85, 1.1 | 1.00 | 0.87 | 0.67, 1.14 | 0.90 | 0.66, 1.22 | 1.03 | 0.75, 1.41 |
| 35 | 1.00 | 0.90 | | 0.81, 1.00 | 0.91 | 0.81, 1 | 0.94 | 0.84, 1.06 | 1.00 | 0.85 | 0.67, 1.09 | 0.92 | 0.71, 1.19 | 0.91 | 0.66, 1.25 |
| 40 | 1.00 | 0.88 | | 0.8, 0.97 | 0.89 | 0.8, 0.97 | 0.88 | 0.78, 0.98 | 1.00 | 0.85 | 0.68, 1.06 | 0.91 | 0.71, 1.16 | 0.84 | 0.63, 1.12 |
| 45 | 1.00 | 0.86 | | 0.8, 0.93 | 0.85 | 0.8, 0.93 | 0.79 | 0.73, 0.86 | 1.00 | 0.86 | 0.73, 1.01 | 0.89 | 0.74, 1.07 | 0.78 | 0.63, 0.97 |
| 50 | 1.00 | 0.85 | | 0.8, 0.89 | 0.79 | 0.8, 0.89 | 0.70 | 0.64, 0.75 | 1.00 | 0.87 | 0.77, 0.99 | 0.86 | 0.74, 0.99 | 0.73 | 0.61, 0.88 |
| 55 | 1.00 | 0.83 | | 0.78, 0.88 | 0.74 | 0.78, 0.88 | 0.63 | 0.57, 0.69 | 1.00 | 0.89 | 0.77, 1.03 | 0.83 | 0.7, 0.98 | 0.71 | 0.57, 0.88 |
| 60 | 1.00 | 0.82 | | 0.78, 0.85 | 0.72 | 0.78, 0.85 | 0.63 | 0.58, 0.69 | 1.00 | 0.90 | 0.81, 0.99 | 0.83 | 0.73, 0.94 | 0.74 | 0.62, 0.89 |
| 65 | 1.00 | 0.80 | | 0.75, 0.86 | 0.71 | 0.75, 0.86 | 0.65 | 0.56, 0.76 | 1.00 | 0.90 | 0.77, 1.05 | 0.83 | 0.67, 1.02 | 0.78 | 0.57, 1.05 |
| **Site-specific cancers (diagnosis or death)** | | | | | | | | | | | | | | | |
| **Melanoma** | | | | | | | | | | | | | | | |
| **Age** | **Ref.** | **HR** | | **95% CI** | **HR** | **95% CI** | **HR** | **95% CI** | **Ref.** | **HR** | **95% CI** | **HR** | **95% CI** | **HR** | **95% CI** |
| 30 | 1.00 | 1.29 | | 0.91, 1.82 | 1.99 | 0.91, 1.82 | 2.70 | 1.99, 3.66 | 1.00 | 0.62 | 0.32, 1.19 | 0.82 | 0.42, 1.59 | 1.19 | 0.6, 2.39 |
| 35 | 1.00 | 1.20 | | 0.99, 1.46 | 1.74 | 0.99, 1.46 | 2.10 | 1.76, 2.5 | 1.00 | 0.82 | 0.56, 1.19 | 1.06 | 0.73, 1.55 | 1.32 | 0.89, 1.96 |
| 40 | 1.00 | 1.14 | | 1, 1.31 | 1.54 | 1, 1.31 | 1.70 | 1.49, 1.94 | 1.00 | 1.02 | 0.75, 1.38 | 1.30 | 0.96, 1.77 | 1.41 | 1.02, 1.95 |
| 45 | 1.00 | 1.11 | | 0.99, 1.26 | 1.37 | 0.99, 1.26 | 1.47 | 1.31, 1.65 | 1.00 | 1.14 | 0.87, 1.49 | 1.40 | 1.07, 1.84 | 1.39 | 1.04, 1.86 |
| 50 | 1.00 | 1.13 | | 1.03, 1.23 | 1.22 | 1.03, 1.23 | 1.40 | 1.27, 1.55 | 1.00 | 1.12 | 0.92, 1.36 | 1.29 | 1.04, 1.6 | 1.25 | 0.98, 1.59 |
| 55 | 1.00 | 1.14 | | 1.04, 1.25 | 1.19 | 1.04, 1.25 | 1.44 | 1.31, 1.59 | 1.00 | 1.09 | 0.89, 1.33 | 1.18 | 0.95, 1.47 | 1.22 | 0.96, 1.56 |
| 60 | 1.00 | 1.13 | | 1.06, 1.21 | 1.21 | 1.06, 1.21 | 1.49 | 1.34, 1.65 | 1.00 | 1.11 | 0.95, 1.3 | 1.15 | 0.95, 1.4 | 1.32 | 1.04, 1.69 |
| 65 | 1.00 | 1.13 | | 1.02, 1.25 | 1.21 | 1.02, 1.25 | 1.51 | 1.28, 1.79 | 1.00 | 1.13 | 0.89, 1.45 | 1.13 | 0.83, 1.53 | 1.40 | 0.95, 2.06 |
| **Non, melanoma** | | | | | | | | | | | | | | | |
| **Age** | **Ref.** | **HR** | | **95% CI** | **HR** | **95% CI** | **HR** | **95% CI** | **Ref.** | **HR** | **95% CI** | **HR** | **95% CI** | **HR** | **95% CI** |
| 30 | 1.00 | 1.26 | | 0.99, 1.6 | 1.68 | 0.99, 1.6 | 2.36 | 1.91, 2.92 | 1.00 | 0.53 | 0.31, 0.92 | 0.71 | 0.42, 1.2 | 1.15 | 0.67, 1.97 |
| 35 | 1.00 | 1.24 | | 1.08, 1.43 | 1.60 | 1.08, 1.43 | 2.13 | 1.87, 2.42 | 1.00 | 0.74 | 0.54, 1.01 | 0.91 | 0.68, 1.24 | 1.19 | 0.87, 1.62 |
| 40 | 1.00 | 1.22 | | 1.12, 1.33 | 1.54 | 1.12, 1.33 | 1.94 | 1.79, 2.11 | 1.00 | 0.98 | 0.81, 1.17 | 1.13 | 0.94, 1.37 | 1.22 | 0.99, 1.5 |
| 45 | 1.00 | 1.19 | | 1.1, 1.28 | 1.41 | 1.1, 1.28 | 1.71 | 1.59, 1.85 | 1.00 | 1.13 | 0.95, 1.34 | 1.23 | 1.03, 1.48 | 1.20 | 0.98, 1.46 |
| 50 | 1.00 | 1.13 | | 1.07, 1.19 | 1.21 | 1.07, 1.19 | 1.42 | 1.34, 1.5 | 1.00 | 1.07 | 0.96, 1.2 | 1.11 | 0.98, 1.25 | 1.11 | 0.97, 1.27 |
| 55 | 1.00 | 1.08 | | 1.03, 1.14 | 1.06 | 1.03, 1.14 | 1.20 | 1.13, 1.29 | 1.00 | 0.96 | 0.85, 1.09 | 0.97 | 0.85, 1.12 | 1.01 | 0.86, 1.18 |
| 60 | 1.00 | 1.12 | | 1.08, 1.16 | 1.18 | 1.08, 1.16 | 1.26 | 1.18, 1.34 | 1.00 | 1.04 | 0.95, 1.14 | 1.11 | 1, 1.23 | 0.98 | 0.85, 1.13 |
| 65 | 1.00 | 1.16 | | 1.1, 1.23 | 1.33 | 1.1, 1.23 | 1.34 | 1.2, 1.49 | 1.00 | 1.14 | 0.99, 1.3 | 1.25 | 1.06, 1.49 | 0.96 | 0.75, 1.22 |
| **Prostate** | | | | | | | | | | | | | | | |
| **Age** | **Ref.** | **HR** | | **95% CI** | **HR** | **95% CI** | **HR** | **95% CI** | **Ref.** | **HR** | **95% CI** | **HR** | **95% CI** | **HR** | **95% CI** |
| 30 | 1.00 | 1.77 | | 0.97, 3.25 | 3.48 | 0.97, 3.25 | 5.91 | 3.27, 10.69 | 1.00 | 7.25 | 1.58, 33.29 | 7.59 | 1.41, 40.78 | 10.06 | 1.44, 70.31 |
| 35 | 1.00 | 1.54 | | 1, 2.37 | 2.48 | 1, 2.37 | 3.69 | 2.43, 5.61 | 1.00 | 4.16 | 1.41, 12.25 | 4.27 | 1.3, 14.05 | 5.25 | 1.33, 20.67 |
| 40 | 1.00 | 1.36 | | 1.02, 1.81 | 1.85 | 1.02, 1.81 | 2.45 | 1.87, 3.22 | 1.00 | 2.56 | 1.27, 5.17 | 2.60 | 1.2, 5.6 | 2.99 | 1.24, 7.19 |
| 45 | 1.00 | 1.22 | | 1.04, 1.43 | 1.43 | 1.04, 1.43 | 1.71 | 1.47, 1.99 | 1.00 | 1.68 | 1.15, 2.44 | 1.67 | 1.11, 2.52 | 1.82 | 1.14, 2.88 |
| 50 | 1.00 | 1.11 | | 1.03, 1.19 | 1.13 | 1.03, 1.19 | 1.24 | 1.14, 1.35 | 1.00 | 1.15 | 0.97, 1.36 | 1.14 | 0.95, 1.36 | 1.17 | 0.95, 1.44 |
| 55 | 1.00 | 1.04 | | 0.98, 1.11 | 1.00 | 0.98, 1.11 | 1.02 | 0.95, 1.1 | 1.00 | 0.96 | 0.84, 1.11 | 0.99 | 0.84, 1.15 | 0.96 | 0.8, 1.16 |
| 60 | 1.00 | 1.04 | | 0.98, 1.09 | 1.04 | 0.98, 1.09 | 1.03 | 0.94, 1.13 | 1.00 | 1.05 | 0.93, 1.19 | 1.14 | 0.99, 1.32 | 1.07 | 0.88, 1.31 |
| 65 | 1.00 | 1.02 | | 0.97, 1.07 | 1.06 | 0.97, 1.07 | 1.10 | 0.99, 1.21 | 1.00 | 0.92 | 0.82, 1.03 | 0.88 | 0.75, 1.03 | 0.84 | 0.67, 1.06 |
| **Oesophagus** | | | | | | | | | | | | | | | |
| **Age** | **Ref.** | **HR** | | **95% CI** | **HR** | **95% CI** | **HR** | **95% CI** | **Ref.** | **HR** | **95% CI** | **HR** | **95% CI** | **HR** | **95% CI** |
| 30 | 1.00 | 1.98 | | 0.6, 6.5 | 0.90 | 0.6, 6.5 | 1.30 | 0.36, 4.75 | 1.00 | , | , | 0.85 | 0.07, 9.85 | 0.24 | 0.02, 2.51 |
| 35 | 1.00 | 1.35 | | 0.63, 2.87 | 0.84 | 0.63, 2.87 | 1.01 | 0.44, 2.31 | 1.00 | , | , | 0.92 | 0.19, 4.49 | 0.36 | 0.08, 1.69 |
| 40 | 1.00 | 0.97 | | 0.62, 1.49 | 0.80 | 0.62, 1.49 | 0.81 | 0.5, 1.33 | 1.00 | 0.82 | 0.01, 103.35 | 0.99 | 0.35, 2.84 | 0.50 | 0.17, 1.49 |
| 45 | 1.00 | 0.74 | | 0.53, 1.03 | 0.75 | 0.53, 1.03 | 0.67 | 0.45, 0.99 | 1.00 | 0.92 | 0.21, 4.13 | 1.02 | 0.4, 2.58 | 0.65 | 0.24, 1.75 |
| 50 | 1.00 | 0.68 | | 0.52, 0.9 | 0.66 | 0.52, 0.9 | 0.54 | 0.38, 0.76 | 1.00 | 0.84 | 0.4, 1.78 | 0.84 | 0.39, 1.81 | 0.67 | 0.28, 1.56 |
| 55 | 1.00 | 0.77 | | 0.63, 0.95 | 0.54 | 0.63, 0.95 | 0.42 | 0.29, 0.63 | 1.00 | 0.75 | 0.47, 1.2 | 0.50 | 0.26, 0.98 | 0.51 | 0.25, 1.05 |
| 60 | 1.00 | 0.79 | | 0.65, 0.96 | 0.54 | 0.65, 0.96 | 0.45 | 0.31, 0.65 | 1.00 | 0.81 | 0.52, 1.27 | 0.53 | 0.3, 0.96 | 0.62 | 0.31, 1.22 |
| 65 | 1.00 | 0.69 | | 0.53, 0.9 | 0.62 | 0.53, 0.9 | 0.56 | 0.31, 1 | 1.00 | 1.01 | 0.51, 1.97 | 0.91 | 0.41, 2.02 | 1.19 | 0.4, 3.6 |
| **Stomach** | | | | | | | | | | | | | | | |
| **Age** | **Ref.** | **HR** | | **95% CI** | **HR** | **95% CI** | **HR** | **95% CI** | **Ref.** | **HR** | **95% CI** | **HR** | **95% CI** | **HR** | **95% CI** |
| 30 | 1.00 | 1.30 | | 0.65, 2.61 | 0.44 | 0.65, 2.61 | 1.24 | 0.6, 2.56 | 1.00 | 0.75 | 0.2, 2.81 | 0.16 | 0.02, 1.41 | 3.43 | 0.84, 14.12 |
| 35 | 1.00 | 1.10 | | 0.72, 1.68 | 0.61 | 0.72, 1.68 | 1.04 | 0.66, 1.64 | 1.00 | 0.83 | 0.36, 1.9 | 0.36 | 0.09, 1.38 | 1.99 | 0.8, 4.94 |
| 40 | 1.00 | 0.95 | | 0.7, 1.3 | 0.82 | 0.7, 1.3 | 0.90 | 0.63, 1.28 | 1.00 | 0.90 | 0.46, 1.77 | 0.70 | 0.28, 1.75 | 1.25 | 0.49, 3.2 |
| 45 | 1.00 | 0.87 | | 0.65, 1.17 | 0.93 | 0.65, 1.17 | 0.78 | 0.56, 1.1 | 1.00 | 0.96 | 0.49, 1.88 | 1.04 | 0.45, 2.39 | 0.87 | 0.34, 2.22 |
| 50 | 1.00 | 0.86 | | 0.7, 1.05 | 0.81 | 0.7, 1.05 | 0.68 | 0.52, 0.88 | 1.00 | 0.99 | 0.63, 1.58 | 0.95 | 0.54, 1.65 | 0.69 | 0.35, 1.34 |
| 55 | 1.00 | 0.86 | | 0.69, 1.06 | 0.63 | 0.69, 1.06 | 0.63 | 0.46, 0.87 | 1.00 | 0.98 | 0.63, 1.51 | 0.59 | 0.32, 1.1 | 0.71 | 0.35, 1.47 |
| 60 | 1.00 | 0.75 | | 0.64, 0.88 | 0.71 | 0.64, 0.88 | 0.66 | 0.5, 0.88 | 1.00 | 0.88 | 0.62, 1.25 | 0.58 | 0.36, 0.93 | 0.93 | 0.53, 1.62 |
| 65 | 1.00 | 0.62 | | 0.47, 0.81 | 0.85 | 0.47, 0.81 | 0.70 | 0.42, 1.18 | 1.00 | 0.76 | 0.39, 1.45 | 0.64 | 0.28, 1.46 | 1.24 | 0.45, 3.41 |
| **Colon** | | | | | | | | | | | | | | | |
| **Age** | **Ref.** | **HR** | | **95% CI** | **HR** | **95% CI** | **HR** | **95% CI** | **Ref.** | **HR** | **95% CI** | **HR** | **95% CI** | **HR** | **95% CI** |
| 30 | 1.00 | 1.23 | | 0.77, 1.97 | 1.33 | 0.77, 1.97 | 1.67 | 1.08, 2.58 | 1.00 | 2.66 | 0.88, 8.04 | 3.61 | 1.27, 10.22 | 3.25 | 0.97, 10.92 |
| 35 | 1.00 | 1.16 | | 0.88, 1.52 | 1.20 | 0.88, 1.52 | 1.23 | 0.95, 1.6 | 1.00 | 1.69 | 0.86, 3.32 | 2.00 | 1.06, 3.78 | 1.83 | 0.85, 3.91 |
| 40 | 1.00 | 1.09 | | 0.92, 1.3 | 1.09 | 0.92, 1.3 | 0.95 | 0.79, 1.15 | 1.00 | 1.15 | 0.76, 1.73 | 1.21 | 0.8, 1.82 | 1.11 | 0.68, 1.83 |
| 45 | 1.00 | 1.01 | | 0.86, 1.18 | 0.97 | 0.86, 1.18 | 0.79 | 0.66, 0.93 | 1.00 | 0.87 | 0.61, 1.22 | 0.86 | 0.6, 1.22 | 0.79 | 0.53, 1.19 |
| 50 | 1.00 | 0.89 | | 0.8, 1 | 0.81 | 0.8, 1 | 0.70 | 0.62, 0.81 | 1.00 | 0.75 | 0.59, 0.96 | 0.75 | 0.57, 0.99 | 0.69 | 0.51, 0.95 |
| 55 | 1.00 | 0.82 | | 0.73, 0.92 | 0.71 | 0.73, 0.92 | 0.68 | 0.58, 0.8 | 1.00 | 0.78 | 0.6, 1.02 | 0.81 | 0.59, 1.11 | 0.71 | 0.48, 1.05 |
| 60 | 1.00 | 0.87 | | 0.8, 0.95 | 0.79 | 0.8, 0.95 | 0.69 | 0.59, 0.81 | 1.00 | 0.90 | 0.74, 1.09 | 0.94 | 0.74, 1.19 | 0.69 | 0.49, 0.98 |
| 65 | 1.00 | 0.95 | | 0.84, 1.08 | 0.92 | 0.84, 1.08 | 0.70 | 0.53, 0.93 | 1.00 | 1.02 | 0.77, 1.35 | 1.05 | 0.72, 1.52 | 0.66 | 0.36, 1.21 |
| **Rectum** | | | | | | | | | | | | | | | |
| **Age** | **Ref.** | **HR** | | **95% CI** | **HR** | **95% CI** | **HR** | **95% CI** | **Ref.** | **HR** | **95% CI** | **HR** | **95% CI** | **HR** | **95% CI** |
| 30 | 1.00 | 0.83 | | 0.41, 1.67 | 1.35 | 0.41, 1.67 | 1.22 | 0.63, 2.33 | 1.00 | 2.87 | 0.75, 10.93 | 6.85 | 1.75, 26.78 | 10.14 | 2.68, 38.41 |
| 35 | 1.00 | 0.93 | | 0.6, 1.42 | 1.21 | 0.6, 1.42 | 1.12 | 0.75, 1.66 | 1.00 | 1.93 | 0.81, 4.57 | 2.80 | 1.16, 6.76 | 3.47 | 1.45, 8.33 |
| 40 | 1.00 | 1.02 | | 0.81, 1.3 | 1.10 | 0.81, 1.3 | 1.04 | 0.82, 1.31 | 1.00 | 1.37 | 0.8, 2.33 | 1.29 | 0.74, 2.27 | 1.37 | 0.76, 2.48 |
| 45 | 1.00 | 1.07 | | 0.89, 1.3 | 1.01 | 0.89, 1.3 | 0.95 | 0.77, 1.17 | 1.00 | 0.99 | 0.66, 1.51 | 0.73 | 0.47, 1.15 | 0.68 | 0.42, 1.1 |
| 50 | 1.00 | 1.00 | | 0.87, 1.16 | 0.93 | 0.87, 1.16 | 0.83 | 0.7, 0.98 | 1.00 | 0.72 | 0.53, 0.98 | 0.63 | 0.46, 0.87 | 0.51 | 0.35, 0.74 |
| 55 | 1.00 | 0.87 | | 0.77, 0.99 | 0.87 | 0.77, 0.99 | 0.72 | 0.6, 0.88 | 1.00 | 0.63 | 0.46, 0.87 | 0.83 | 0.6, 1.16 | 0.62 | 0.4, 0.97 |
| 60 | 1.00 | 0.90 | | 0.81, 0.99 | 0.81 | 0.81, 0.99 | 0.74 | 0.62, 0.89 | 1.00 | 0.88 | 0.71, 1.1 | 0.89 | 0.67, 1.17 | 0.59 | 0.4, 0.87 |
| 65 | 1.00 | 1.00 | | 0.86, 1.16 | 0.75 | 0.86, 1.16 | 0.82 | 0.59, 1.13 | 1.00 | 1.32 | 0.95, 1.84 | 0.85 | 0.53, 1.37 | 0.48 | 0.21, 1.09 |
| **Liver, bile ducts, and gallbladder** | | | | | | | | | | | | | | | |
| **Age** | **Ref.** | **HR** | | **95% CI** | **HR** | **95% CI** | **HR** | **95% CI** | **Ref.** | **HR** | **95% CI** | **HR** | **95% CI** | **HR** | **95% CI** |
| 30 | 1.00 | 0.76 | | 0.4, 1.45 | 0.62 | 0.4, 1.45 | 0.90 | 0.48, 1.68 | 1.00 | 3.23 | 0.66, 15.7 | 1.79 | 0.22, 14.33 | 0.46 | 0.05, 4.52 |
| 35 | 1.00 | 0.75 | | 0.51, 1.12 | 0.66 | 0.51, 1.12 | 0.88 | 0.59, 1.31 | 1.00 | 2.30 | 0.81, 6.52 | 1.46 | 0.42, 5.12 | 0.55 | 0.13, 2.3 |
| 40 | 1.00 | 0.75 | | 0.58, 0.97 | 0.71 | 0.58, 0.97 | 0.86 | 0.64, 1.16 | 1.00 | 1.71 | 0.84, 3.48 | 1.22 | 0.62, 2.41 | 0.64 | 0.28, 1.47 |
| 45 | 1.00 | 0.74 | | 0.58, 0.96 | 0.71 | 0.58, 0.96 | 0.79 | 0.59, 1.06 | 1.00 | 1.34 | 0.73, 2.47 | 1.06 | 0.6, 1.87 | 0.73 | 0.38, 1.41 |
| 50 | 1.00 | 0.74 | | 0.61, 0.89 | 0.66 | 0.61, 0.89 | 0.63 | 0.5, 0.79 | 1.00 | 1.13 | 0.72, 1.77 | 0.98 | 0.6, 1.6 | 0.77 | 0.44, 1.34 |
| 55 | 1.00 | 0.73 | | 0.63, 0.84 | 0.57 | 0.63, 0.84 | 0.43 | 0.32, 0.58 | 1.00 | 1.01 | 0.69, 1.49 | 0.95 | 0.61, 1.48 | 0.76 | 0.43, 1.35 |
| 60 | 1.00 | 0.76 | | 0.66, 0.88 | 0.54 | 0.66, 0.88 | 0.42 | 0.32, 0.56 | 1.00 | 1.00 | 0.72, 1.39 | 0.93 | 0.62, 1.38 | 0.84 | 0.49, 1.42 |
| 65 | 1.00 | 0.84 | | 0.71, 1 | 0.58 | 0.71, 1 | 0.60 | 0.39, 0.91 | 1.00 | 1.02 | 0.67, 1.55 | 0.89 | 0.49, 1.6 | 1.01 | 0.45, 2.27 |
| **Pancreas** | | | | | | | | | | | | | | | |
| **Age** | **Ref.** | **HR** | | **95% CI** | **HR** | **95% CI** | **HR** | **95% CI** | **Ref.** | **HR** | **95% CI** | **HR** | **95% CI** | **HR** | **95% CI** |
| 30 | 1.00 | 0.86 | | 0.38, 1.91 | 1.40 | 0.38, 1.91 | 1.24 | 0.58, 2.66 | 1.00 | 1.87 | 0.25, 14.2 | 4.29 | 0.6, 30.82 | 3.31 | 0.47, 23.45 |
| 35 | 1.00 | 0.88 | | 0.53, 1.46 | 1.10 | 0.53, 1.46 | 0.97 | 0.6, 1.58 | 1.00 | 1.26 | 0.33, 4.75 | 2.13 | 0.59, 7.75 | 1.47 | 0.41, 5.33 |
| 40 | 1.00 | 0.91 | | 0.67, 1.22 | 0.90 | 0.67, 1.22 | 0.79 | 0.57, 1.09 | 1.00 | 0.90 | 0.41, 1.97 | 1.17 | 0.53, 2.57 | 0.73 | 0.31, 1.74 |
| 45 | 1.00 | 0.92 | | 0.72, 1.17 | 0.78 | 0.72, 1.17 | 0.67 | 0.5, 0.91 | 1.00 | 0.72 | 0.43, 1.21 | 0.75 | 0.42, 1.34 | 0.45 | 0.22, 0.95 |
| 50 | 1.00 | 0.91 | | 0.74, 1.11 | 0.79 | 0.74, 1.11 | 0.64 | 0.5, 0.81 | 1.00 | 0.83 | 0.56, 1.23 | 0.73 | 0.47, 1.14 | 0.49 | 0.29, 0.83 |
| 55 | 1.00 | 0.87 | | 0.75, 1.02 | 0.90 | 0.75, 1.02 | 0.66 | 0.51, 0.85 | 1.00 | 1.44 | 0.96, 2.15 | 1.15 | 0.72, 1.84 | 0.93 | 0.52, 1.65 |
| 60 | 1.00 | 0.84 | | 0.73, 0.97 | 0.88 | 0.73, 0.97 | 0.77 | 0.61, 0.98 | 1.00 | 1.31 | 0.95, 1.79 | 1.11 | 0.76, 1.63 | 1.18 | 0.74, 1.89 |
| 65 | 1.00 | 0.82 | | 0.68, 0.99 | 0.72 | 0.68, 0.99 | 0.98 | 0.69, 1.4 | 1.00 | 0.78 | 0.49, 1.24 | 0.80 | 0.45, 1.42 | 1.25 | 0.63, 2.49 |
| **Head and neck** | | | | | | | | | | | | | | | |
| **Age** | **Ref.** | **HR** | | **95% CI** | **HR** | **95% CI** | **HR** | **95% CI** | **Ref.** | **HR** | **95% CI** | **HR** | **95% CI** | **HR** | **95% CI** |
| 30 | 1.00 | 1.51 | | 0.93, 2.46 | 2.09 | 0.93, 2.46 | 2.32 | 1.47, 3.65 | 1.00 | 0.75 | 0.31, 1.78 | 0.74 | 0.27, 2.01 | 0.47 | 0.15, 1.46 |
| 35 | 1.00 | 1.09 | | 0.83, 1.45 | 1.28 | 0.83, 1.45 | 1.44 | 1.1, 1.89 | 1.00 | 0.71 | 0.44, 1.16 | 0.70 | 0.4, 1.24 | 0.58 | 0.3, 1.13 |
| 40 | 1.00 | 0.85 | | 0.7, 1.03 | 0.87 | 0.7, 1.03 | 0.96 | 0.78, 1.18 | 1.00 | 0.69 | 0.47, 1.03 | 0.68 | 0.44, 1.03 | 0.69 | 0.42, 1.11 |
| 45 | 1.00 | 0.74 | | 0.62, 0.88 | 0.70 | 0.62, 0.88 | 0.71 | 0.59, 0.85 | 1.00 | 0.70 | 0.48, 1.02 | 0.68 | 0.46, 1.01 | 0.71 | 0.46, 1.1 |
| 50 | 1.00 | 0.74 | | 0.66, 0.84 | 0.67 | 0.66, 0.84 | 0.58 | 0.5, 0.68 | 1.00 | 0.74 | 0.57, 0.97 | 0.72 | 0.53, 0.98 | 0.63 | 0.43, 0.92 |
| 55 | 1.00 | 0.82 | | 0.72, 0.93 | 0.74 | 0.72, 0.93 | 0.59 | 0.49, 0.71 | 1.00 | 0.86 | 0.65, 1.15 | 0.80 | 0.58, 1.12 | 0.67 | 0.44, 1.01 |
| 60 | 1.00 | 0.90 | | 0.82, 0.99 | 0.83 | 0.82, 0.99 | 0.73 | 0.61, 0.89 | 1.00 | 1.18 | 0.93, 1.51 | 0.99 | 0.73, 1.36 | 1.07 | 0.71, 1.61 |
| 65 | 1.00 | 0.97 | | 0.83, 1.14 | 0.91 | 0.83, 1.14 | 0.88 | 0.64, 1.2 | 1.00 | 1.55 | 1.03, 2.32 | 1.18 | 0.69, 2.01 | 1.57 | 0.8, 3.09 |
| **Kidney** | | | | | | | | | | | | | | | |
| **Age** | **Ref.** | **HR** | | **95% CI** | **HR** | **95% CI** | **HR** | **95% CI** | **Ref.** | **HR** | **95% CI** | **HR** | **95% CI** | **HR** | **95% CI** |
| 30 | 1.00 | 0.56 | | 0.27, 1.15 | 0.72 | 0.27, 1.15 | 0.86 | 0.45, 1.63 | 1.00 | 0.69 | 0.16, 3.01 | 0.49 | 0.13, 1.85 | 0.95 | 0.2, 4.44 |
| 35 | 1.00 | 0.76 | | 0.5, 1.17 | 0.84 | 0.5, 1.17 | 0.83 | 0.56, 1.22 | 1.00 | 0.98 | 0.43, 2.24 | 0.81 | 0.37, 1.78 | 1.08 | 0.44, 2.65 |
| 40 | 1.00 | 0.99 | | 0.77, 1.27 | 0.96 | 0.77, 1.27 | 0.81 | 0.62, 1.05 | 1.00 | 1.30 | 0.79, 2.16 | 1.24 | 0.7, 2.19 | 1.20 | 0.6, 2.42 |
| 45 | 1.00 | 1.09 | | 0.88, 1.36 | 0.98 | 0.88, 1.36 | 0.76 | 0.59, 0.97 | 1.00 | 1.32 | 0.83, 2.11 | 1.39 | 0.82, 2.34 | 1.13 | 0.59, 2.18 |
| 50 | 1.00 | 0.91 | | 0.78, 1.07 | 0.81 | 0.78, 1.07 | 0.66 | 0.55, 0.79 | 1.00 | 0.97 | 0.69, 1.36 | 1.05 | 0.73, 1.51 | 0.89 | 0.58, 1.36 |
| 55 | 1.00 | 0.69 | | 0.59, 0.81 | 0.62 | 0.59, 0.81 | 0.58 | 0.46, 0.72 | 1.00 | 0.71 | 0.5, 1.03 | 0.71 | 0.47, 1.06 | 0.69 | 0.42, 1.16 |
| 60 | 1.00 | 0.78 | | 0.7, 0.88 | 0.64 | 0.7, 0.88 | 0.64 | 0.51, 0.79 | 1.00 | 1.00 | 0.77, 1.3 | 0.76 | 0.54, 1.09 | 0.77 | 0.48, 1.24 |
| 65 | 1.00 | 0.95 | | 0.8, 1.14 | 0.71 | 0.8, 1.14 | 0.73 | 0.49, 1.08 | 1.00 | 1.52 | 0.99, 2.34 | 0.91 | 0.48, 1.72 | 0.93 | 0.39, 2.19 |
| **Myeloma** | | | | | | | | | | | | | | | |
| **Age** | **Ref.** | **HR** | | **95% CI** | **HR** | **95% CI** | **HR** | **95% CI** | **Ref.** | **HR** | **95% CI** | **HR** | **95% CI** | **HR** | **95% CI** |
| 30 | 1.00 | 1.85 | | 0.67, 5.15 | 1.37 | 0.67, 5.15 | 1.78 | 0.63, 4.99 | 1.00 | 0.23 | 0.05, 1.02 | 0.26 | 0.05, 1.37 | 0.68 | 0.14, 3.23 |
| 35 | 1.00 | 1.45 | | 0.78, 2.71 | 1.17 | 0.78, 2.71 | 1.44 | 0.77, 2.73 | 1.00 | 0.36 | 0.14, 0.91 | 0.36 | 0.12, 1.1 | 0.66 | 0.23, 1.89 |
| 40 | 1.00 | 1.18 | | 0.82, 1.69 | 1.01 | 0.82, 1.69 | 1.21 | 0.82, 1.77 | 1.00 | 0.52 | 0.24, 1.1 | 0.47 | 0.19, 1.16 | 0.64 | 0.25, 1.63 |
| 45 | 1.00 | 1.01 | | 0.75, 1.37 | 0.93 | 0.75, 1.37 | 1.04 | 0.74, 1.46 | 1.00 | 0.74 | 0.33, 1.66 | 0.62 | 0.26, 1.53 | 0.69 | 0.26, 1.8 |
| 50 | 1.00 | 0.97 | | 0.77, 1.23 | 0.95 | 0.77, 1.23 | 0.94 | 0.72, 1.23 | 1.00 | 1.10 | 0.61, 1.99 | 0.92 | 0.49, 1.71 | 0.95 | 0.45, 1.99 |
| 55 | 1.00 | 1.02 | | 0.82, 1.28 | 1.05 | 0.82, 1.28 | 0.92 | 0.66, 1.26 | 1.00 | 1.24 | 0.67, 2.31 | 1.15 | 0.59, 2.24 | 1.24 | 0.54, 2.84 |
| 60 | 1.00 | 0.98 | | 0.83, 1.15 | 1.06 | 0.83, 1.15 | 0.97 | 0.74, 1.26 | 1.00 | 0.78 | 0.53, 1.15 | 0.99 | 0.64, 1.54 | 0.76 | 0.42, 1.4 |
| 65 | 1.00 | 0.90 | | 0.71, 1.14 | 1.02 | 0.71, 1.14 | 1.02 | 0.66, 1.6 | 1.00 | 0.45 | 0.25, 0.84 | 0.88 | 0.46, 1.67 | 0.40 | 0.12, 1.31 |
| **Lung** | | | | | | | | | | | | | | | |
| **Age** | **Ref.** | **HR** | | **95% CI** | **HR** | **95% CI** | **HR** | **95% CI** | **Ref.** | **HR** | **95% CI** | **HR** | **95% CI** | **HR** | **95% CI** |
| 30 | 1.00 | 0.90 | | 0.49, 1.64 | 1.50 | 0.49, 1.64 | 2.03 | 1.21, 3.42 | 1.00 | 0.44 | 0.13, 1.46 | 0.81 | 0.17, 3.84 | 0.86 | 0.19, 3.78 |
| 35 | 1.00 | 0.88 | | 0.61, 1.28 | 1.11 | 0.61, 1.28 | 1.29 | 0.92, 1.8 | 1.00 | 0.56 | 0.27, 1.18 | 0.87 | 0.34, 2.27 | 0.85 | 0.33, 2.17 |
| 40 | 1.00 | 0.87 | | 0.69, 1.09 | 0.85 | 0.69, 1.09 | 0.86 | 0.66, 1.13 | 1.00 | 0.70 | 0.43, 1.15 | 0.93 | 0.51, 1.68 | 0.84 | 0.45, 1.59 |
| 45 | 1.00 | 0.83 | | 0.68, 1.01 | 0.70 | 0.68, 1.01 | 0.61 | 0.47, 0.78 | 1.00 | 0.81 | 0.51, 1.3 | 0.94 | 0.55, 1.58 | 0.75 | 0.41, 1.36 |
| 50 | 1.00 | 0.74 | | 0.63, 0.87 | 0.61 | 0.63, 0.87 | 0.43 | 0.35, 0.54 | 1.00 | 0.82 | 0.57, 1.19 | 0.87 | 0.58, 1.29 | 0.54 | 0.33, 0.88 |
| 55 | 1.00 | 0.63 | | 0.55, 0.72 | 0.57 | 0.55, 0.72 | 0.33 | 0.24, 0.44 | 1.00 | 0.75 | 0.56, 1 | 0.74 | 0.5, 1.08 | 0.32 | 0.17, 0.62 |
| 60 | 1.00 | 0.67 | | 0.59, 0.75 | 0.57 | 0.59, 0.75 | 0.34 | 0.27, 0.44 | 1.00 | 0.79 | 0.61, 1.02 | 0.63 | 0.44, 0.91 | 0.36 | 0.22, 0.59 |
| 65 | 1.00 | 0.86 | | 0.74, 0.99 | 0.58 | 0.74, 0.99 | 0.43 | 0.29, 0.66 | 1.00 | 0.94 | 0.67, 1.32 | 0.54 | 0.3, 0.95 | 0.50 | 0.24, 1.04 |
| **Bladder** | | | | | | | | | | | | | | | |
| **Age** | **Ref.** | **HR** | | **95% CI** | **HR** | **95% CI** | **HR** | **95% CI** | **Ref.** | **HR** | **95% CI** | **HR** | **95% CI** | **HR** | **95% CI** |
| 30 | 1.00 | 2.69 | | 1.35, 5.36 | 3.40 | 1.35, 5.36 | 4.58 | 2.39, 8.8 | 1.00 | 2.63 | 0.77, 8.9 | 2.20 | 0.65, 7.43 | 2.63 | 0.61, 11.33 |
| 35 | 1.00 | 1.73 | | 1.14, 2.64 | 2.06 | 1.14, 2.64 | 2.35 | 1.57, 3.53 | 1.00 | 1.72 | 0.8, 3.69 | 1.51 | 0.69, 3.28 | 1.77 | 0.7, 4.46 |
| 40 | 1.00 | 1.18 | | 0.93, 1.51 | 1.33 | 0.93, 1.51 | 1.32 | 1.02, 1.72 | 1.00 | 1.19 | 0.72, 1.97 | 1.09 | 0.63, 1.87 | 1.26 | 0.66, 2.38 |
| 45 | 1.00 | 0.91 | | 0.74, 1.1 | 0.97 | 0.74, 1.1 | 0.84 | 0.67, 1.05 | 1.00 | 0.94 | 0.6, 1.45 | 0.87 | 0.53, 1.4 | 0.93 | 0.53, 1.65 |
| 50 | 1.00 | 0.82 | | 0.7, 0.96 | 0.84 | 0.7, 0.96 | 0.63 | 0.52, 0.76 | 1.00 | 0.88 | 0.64, 1.22 | 0.79 | 0.55, 1.14 | 0.73 | 0.47, 1.12 |
| 55 | 1.00 | 0.89 | | 0.78, 1.02 | 0.87 | 0.78, 1.02 | 0.59 | 0.47, 0.75 | 1.00 | 0.98 | 0.71, 1.33 | 0.83 | 0.58, 1.21 | 0.67 | 0.4, 1.11 |
| 60 | 1.00 | 0.91 | | 0.81, 1.02 | 0.87 | 0.81, 1.02 | 0.64 | 0.53, 0.78 | 1.00 | 0.99 | 0.77, 1.26 | 0.82 | 0.61, 1.09 | 0.85 | 0.57, 1.26 |
| 65 | 1.00 | 0.86 | | 0.75, 0.99 | 0.82 | 0.75, 0.99 | 0.70 | 0.51, 0.96 | 1.00 | 0.94 | 0.65, 1.36 | 0.76 | 0.46, 1.26 | 1.16 | 0.63, 2.13 |
| CI = confidence interval. HR = hazard ratio. Q = quartile. HRs are adjusted for age at conscription, year of conscription, body mass index, parental education, and parental income. In both cohorts, the median (range) of W_max_ in Q1 was 217 (100, 236), in Q2 it was 253 (237, 270), in Q3 it was 290 (271, 312), in Q4 it was 339 (313, 999). | | | | | | | | | | | | | | | |
| ^a^The hazard ratios were allowed to vary across time using an interaction between a restricted cubic spline with three degrees of freedom of the follow-up time (centile 33 and 67 of the distribution of the uncensored log survival times) and the fitness quartiles. | | | | | | | | | | | | | | | |
