# Supplementary material for: Adolescent cardiorespiratory fitness and risk of cancer in late adulthood: A nationwide sibling-controlled cohort study in Sweden
Source: PLoS Med. 2025 May 8;22(5):e1004597. doi: 10.1371/journal.pmed.1004597 (PMC12061154; doi:10.1371/journal.pmed.1004597)
Supplement: S17 Table — (DOCX) [file pmed.1004597.s017.docx]

| **S17 Table**. **Hazard ratios for site-specific cancer mortality by quartiles of cardiorespiratory fitness in cohort analysis.** | | |
| --- | --- | --- |
|  | **Cohort analysis  (N=1 124 049)** | |
| **Cancer-specific mortality  by quartiles of cardiorespiratory fitness** | **Cases, n (%)** | **HR (95% CI)** |
| **Head and neck** |  |  |
| Q1 | 341 (0.12) | Ref. |
| Q2 | 174 (0.06) | 0.61(0.51, 0.74) |
| Q3 | 113 (0.04) | 0.60 (0.48, 0.76) |
| Q4 | 59 (0.02) | 0.47 (0.34, 0.64) |
| **Oesophagus** |  |  |
| Q1 | 317 (0.11) | Ref. |
| Q2 | 197 (0.07) | 0.75 (0.62, 0.90) |
| Q3 | 96 (0.03) | 0.58 (0.45, 0.74) |
| Q4 | 44 (0.02) | 0.42 (0.30, 0.59) |
| **Lung** |  |  |
| Q1 | 990 (0.35) | Ref. |
| Q2 | 490 (0.17) | 0.69 (0.62, 0.77) |
| Q3 | 197 (0.08) | 0.48 (0.41, 0.56) |
| Q4 | 93 (0.03) | 0.39 (0.31, 0.49) |
| **Stomach** |  |  |
| Q1 | 258 (0.09) | Ref. |
| Q2 | 190 (0.07) | 0.89 (0.74, 1.08) |
| Q3 | 103 (0.04) | 0.75 (0.59, 0.96) |
| Q4 | 63 (0.02) | 0.70 (0.51, 0.95) |
| **Pancreas** |  |  |
| Q1 | 658 (0.23) | Ref. |
| Q2 | 418 (0.15) | 0.78 (0.69, 0.89) |
| Q3 | 273 (0.10) | 0.82 (0.70, 0.95) |
| Q4 | 144 (0.05) | 0.70 (0.57, 0.86) |
| **Liver, bile ducts, and gallbladder** |  |  |
| Q1 | 611 (0.22) | Ref. |
| Q2 | 356 (0.12) | 0.71 (0.62, 0.81) |
| Q3 | 168 (0.06) | 0.53 (0.44, 0.63) |
| Q4 | 121 (0.04) | 0.59 (0.47, 0.74) |
| **Colon** |  |  |
| Q1 | 570 (0.20) | Ref. |
| Q2 | 416 (0.15) | 0.86 (0.87, 0.98) |
| Q3 | 268 (0.10) | 0.75 (0.64, 0.88) |
| Q4 | 183 (0.07) | 0.65 (0.54, 0.79) |
| **Rectum** |  |  |
| Q1 | 354 (0.13) | Ref. |
| Q2 | 248 (0.09) | 0.85 (0.72, 1.01) |
| Q3 | 163 (0.06) | 0.82 (0.67, 1.00) |
| Q4 | 83 (0.03) | 0.58 (0.44, 0.76) |
| **Kidney** |  |  |
| Q1 | 230 (0.08) | Ref. |
| Q2 | 150 (0.05) | 0.73 (0.59, 0.90) |
| Q3 | 102 (0.04) | 0.73 (0.57, 0.94) |
| Q4 | 60 (0.02) | 0.62 (0.45, 0.85) |
| **Prostate** |  |  |
| Q1 | 391 (0.14) | Ref. |
| Q2 | 289 (0.10) | 1.03 (0.88, 1.21) |
| Q3 | 125 (0.05) | 0.84 (0.68, 1.03) |
| Q4 | 63 (0.02) | 0.87 (0.65, 1.16) |
| **Myeloma** |  |  |
| Q1 | 132 (0.05) | Ref. |
| Q2 | 124 (0.04) | 1.17 (0.91, 1.50) |
| Q3 | 64 (0.02) | 0.98 (0.71, 1.34) |
| Q4 | 40 (0.01) | 1.02 (0.68, 1.52) |
| **Melanoma skin** |  |  |
| Q1 | 288 (0.10) | Ref. |
| Q2 | 246 (0.09) | 0.99 (0.83, 1.18 |
| Q3 | 194 (0.07) | 1.07 (0.88, 1.31) |
| Q4 | 147 (0.05) | 1.06 (0.84, 1.34) |
| CI = confidence interval. HR = hazard ratio. Q = quartile. HRs are adjusted for age at conscription, year of conscription, body mass index, parental education, and parental income. In both cohorts, the median (range) of W_max_ in Q1 was 217 (100-236), in Q2 it was 253 (237-270), in Q3 it was 290 (271-312), in Q4 it was 339 (313-999). | | |
