# Supplementary figures and images for: Adolescent cardiorespiratory fitness and risk of cancer in late adulthood: A nationwide sibling-controlled cohort study in Sweden
Source: PLoS Med. 2025 May 8;22(5):e1004597. doi: 10.1371/journal.pmed.1004597 (PMC12061154; doi:10.1371/journal.pmed.1004597)

**
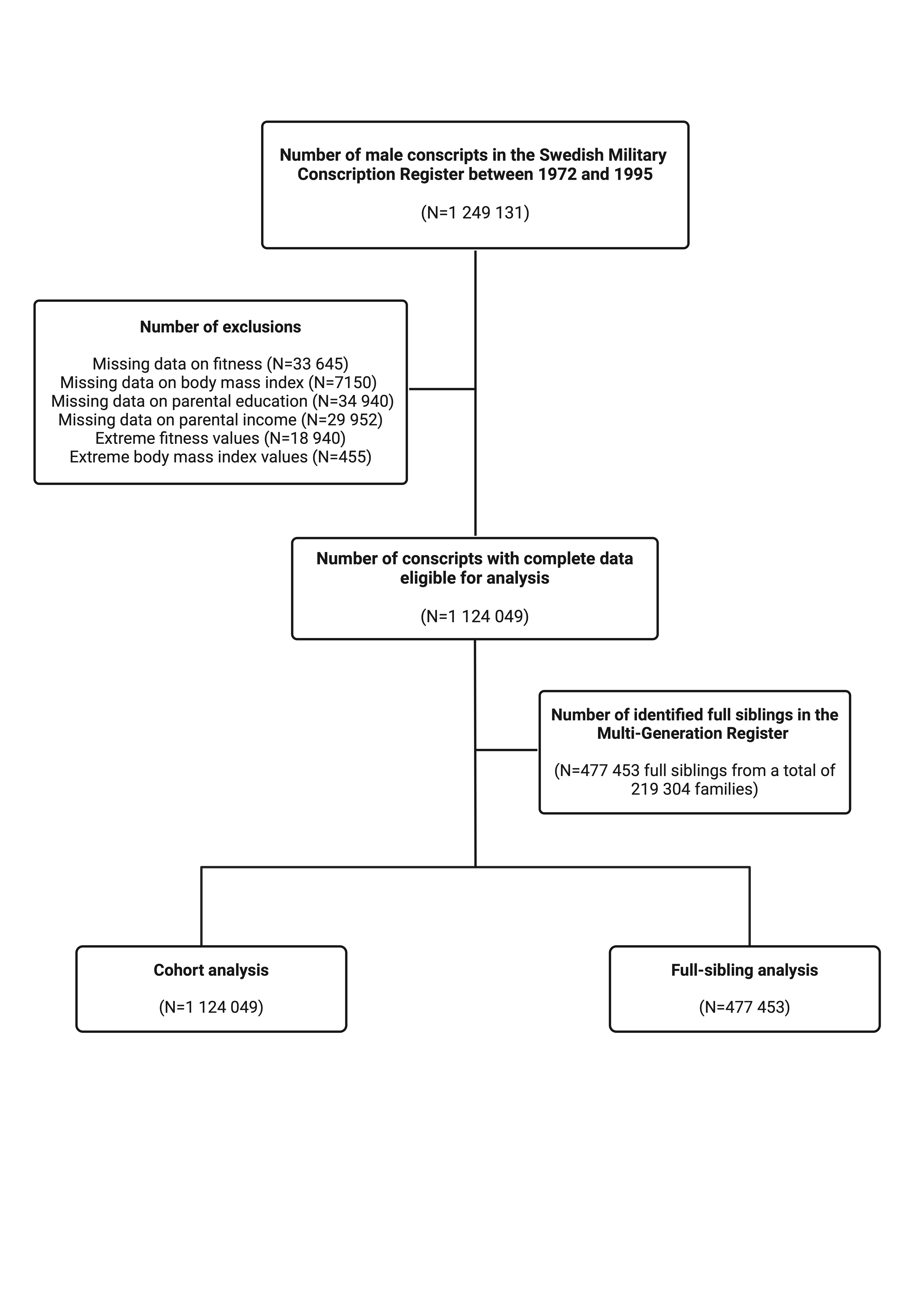
**

**S1 Fig. Participant flow chart.** Created in BioRender. Ahlqvist, V. (2025) <https://BioRender.com/zd6mzpp>

Supplement: S1 Fig — (DOCX) [file pmed.1004597.s018.docx]
