# Supplementary material for: Adolescent cardiorespiratory fitness and risk of cancer in late adulthood: A nationwide sibling-controlled cohort study in Sweden
Source: PLoS Med. 2025 May 8;22(5):e1004597. doi: 10.1371/journal.pmed.1004597 (PMC12061154; doi:10.1371/journal.pmed.1004597)
Supplement: S2 Fig — (DOCX) [file pmed.1004597.s019.docx]

**
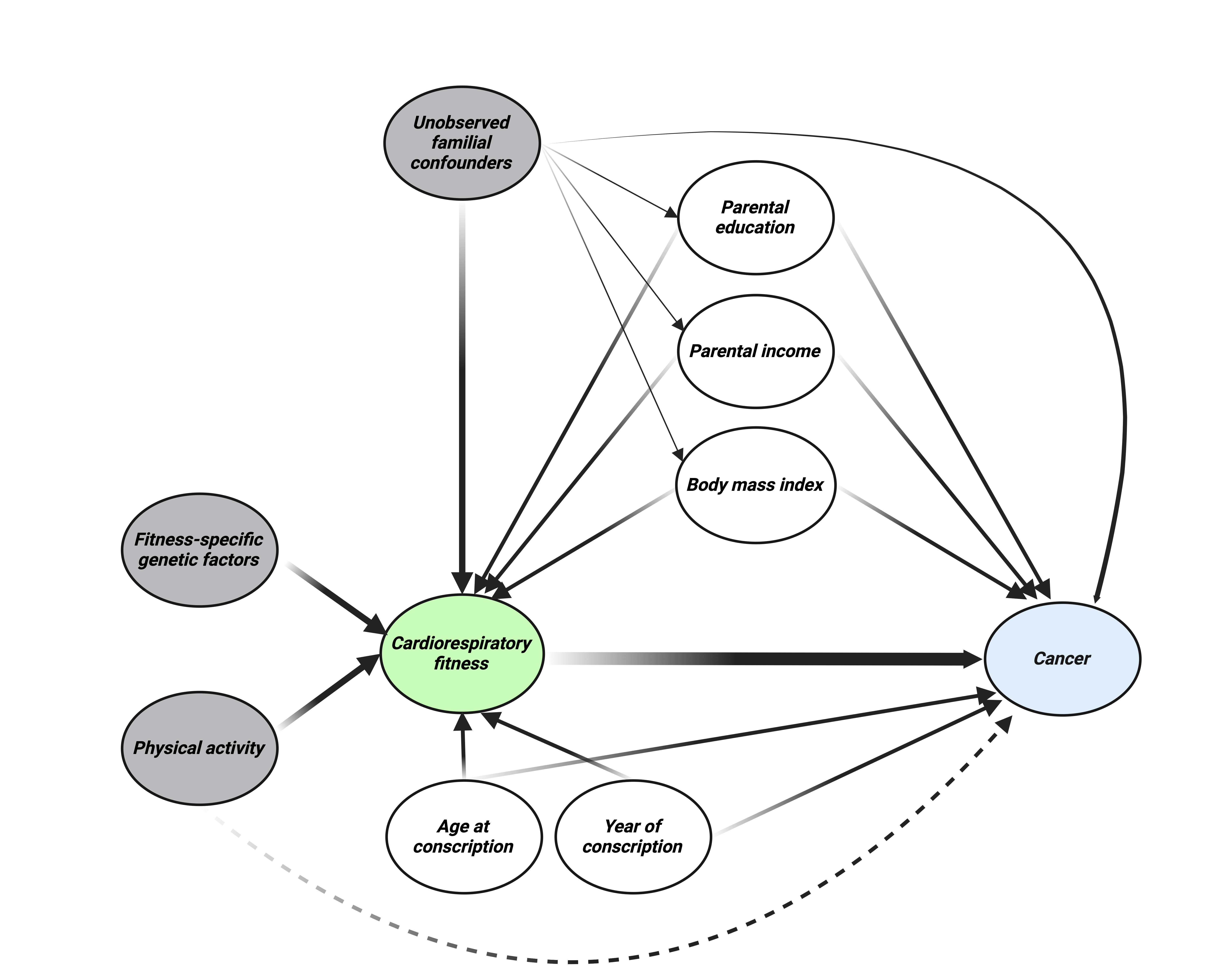
**

**S2 Fig. Directed acyclic graph for the association between adolescent cardiorespiratory fitness and risk of cancer in late adulthood, where observed (white) and unobserved (gray) confounders are illustrated. We primarily viewed physical activity as a (near) instrumental variable for fitness, meaning that physical activity has a causal effect on fitness and only lowers the risk of cancer via its effect on fitness and not independently. However, physical activity could also be postulated to have certain effects that lower the risk of cancer independent of fitness, which would make it a confounder.**

Created in BioRender. Ahlqvist, V. (2025) <https://BioRender.com/1y07w3x> .
