# Supplementary material for: Adolescent cardiorespiratory fitness and risk of cancer in late adulthood: A nationwide sibling-controlled cohort study in Sweden
Source: PLoS Med. 2025 May 8;22(5):e1004597. doi: 10.1371/journal.pmed.1004597 (PMC12061154; doi:10.1371/journal.pmed.1004597)
Supplement: S3 Fig — (DOCX) [file pmed.1004597.s020.docx]

**
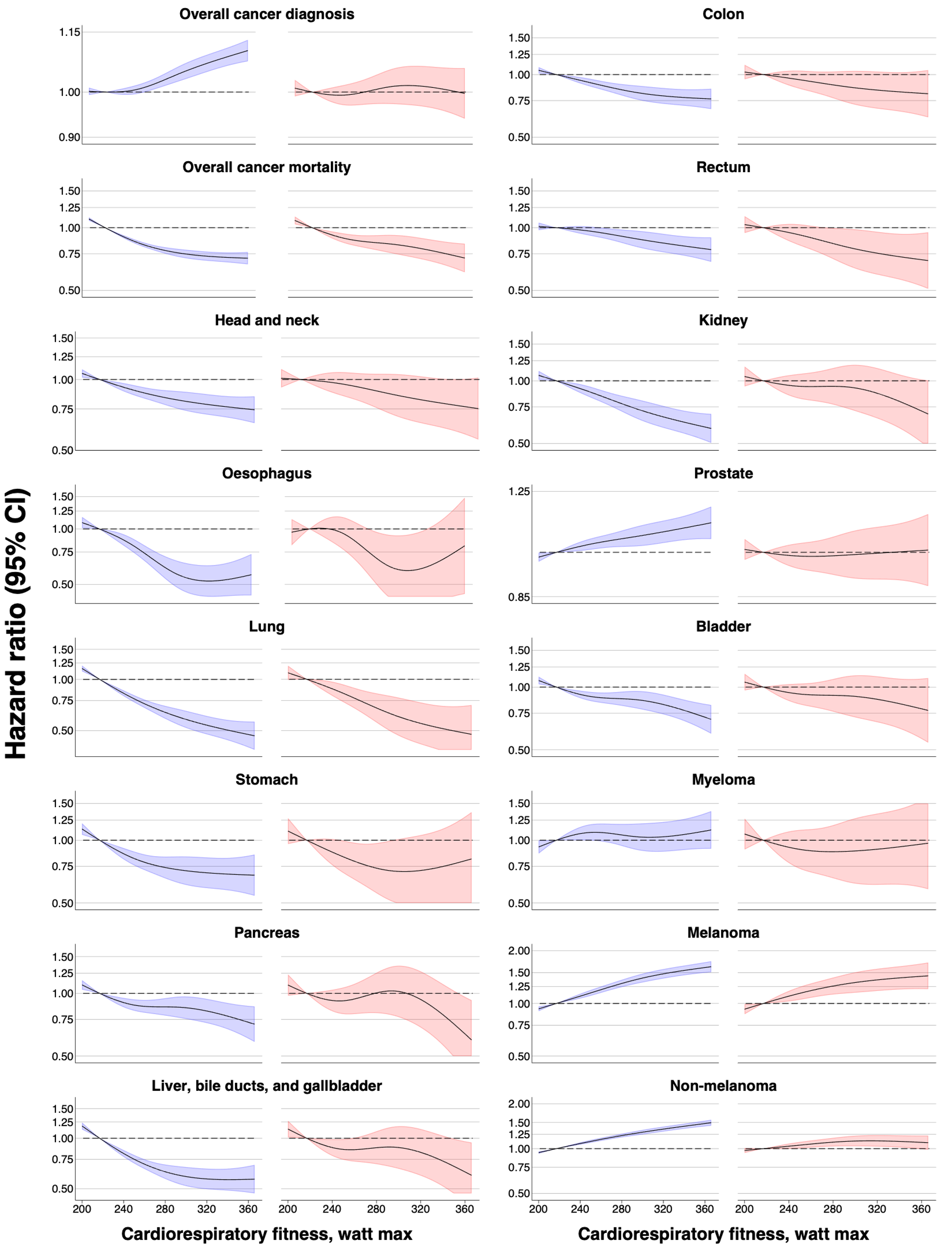
**

**S3 Fig. Hazard ratios for overall cancer diagnosis and mortality and site-specific cancer (diagnosis or death) across restricted cubic splines of cardiorespiratory fitness in cohort (blue) and sibling analysis (red).** Estimates were obtained using flexible parametric survival models, extended to a marginalized between-within model in the sibling cohort, with knots placed at the 5^th^, 35^th^, 65^th^, and 95^th^ percentile, and using age as the underlying time scale. The referent was set to the median value of the bottom quartile (217 W_max_). The models were adjusted for age at conscription, year of conscription, body mass index, parental education, and parental income. For graphical purposes, the x-axis was limited to span from the 5^th^ to the 95^th^ percentile of the distribution.
