# Supplementary material for: Adolescent cardiorespiratory fitness and risk of cancer in late adulthood: A nationwide sibling-controlled cohort study in Sweden
Source: PLoS Med. 2025 May 8;22(5):e1004597. doi: 10.1371/journal.pmed.1004597 (PMC12061154; doi:10.1371/journal.pmed.1004597)
Supplement: S4 Fig — (DOCX) [file pmed.1004597.s021.docx]

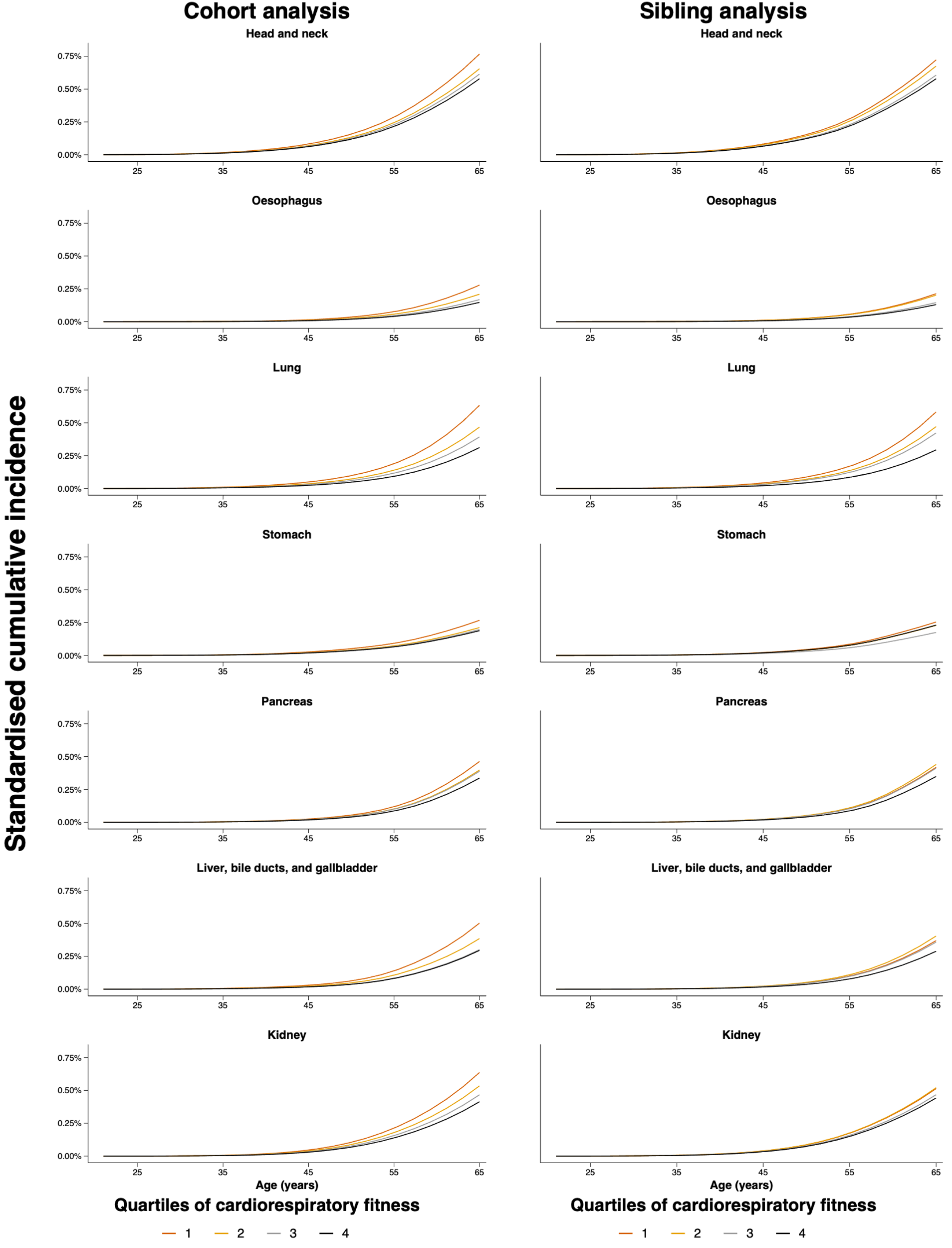


**S4 Fig. Standardized cumulative incidence for site-specific cancer (diagnosis or death) by quartiles of cardiorespiratory fitness in cohort and sibling analysis.** Estimates obtained using flexible parametric survival models, extended to a marginalized between-within model in the sibling cohort, with baseline knots placed at the 5^th^, 27.5^th^, 50^th^, 72.5^th^, and 95^th^ percentile of the uncensored log survival times, and using age as the underlying time scale. All models were adjusted for age at conscription, year of conscription, body mass index, parental education, and parental income. Inferential measures for the incidences were omitted for clarity as they are also reflected in S5 and S6 Tables.
