# Supplementary material for: Adolescent cardiorespiratory fitness and risk of cancer in late adulthood: A nationwide sibling-controlled cohort study in Sweden
Source: PLoS Med. 2025 May 8;22(5):e1004597. doi: 10.1371/journal.pmed.1004597 (PMC12061154; doi:10.1371/journal.pmed.1004597)
Supplement: Attachment — Submitted filename: Authors responses.docx [file pmed.1004597.s027.docx]

# Comments from the reviewers:

## Reviewer #1:

Using a Swedish population-level conscription database (and other linked datasets), this research investigated the relationship between adolescent cardiovascular fitness and time to subsequent cancer diagnoses and mortality, controlling for key, known confounders. Differentiating this research from earlier, similar research, the authors controlled for some unobserved confounding using between-within modelling of sibling clusters (in addition to modelling broader cohort) and flexible parametric survival models allowing estimation of baseline hazard instead of the more traditional Cox regression. Overall, this manuscript is an excellent initial submission. It is clearly written, logically presented, and cogently argued. The high level of care taken by the authors to use methods that produce more interpretable and rigorous results is laudable. Relatedly, the authors have recognised some of their methods are relatively novel and cited appropriate methodological work providing helpful background and justifying their choices well. That said, there are some areas of potential confusion and opportunities to improve clarity. Detailed feedback on the manuscript is provided below. My primary focus has been methodological, but I have made a few broader points as well.  All items are major unless indicated otherwise.

Response: We thank the reviewer for their endorsement of our paper and for their constructive comments which we believe have helped improve our manuscript. We have done our best to resolve the areas of potential confusion and attempted to improve the paper further.

1. The authors prosecute a compelling argument against analysing composite endpoints of cancer detection and survival. They then use the precise approach they argued against in their specific cancer analyses. While they note this apparent contradiction in their discussion, it is not sufficiently explained or justified; noting, of course, insufficient sample size is no justification for poor analytical choices. Suggest the authors provide a compelling argument as to why they are going against their own recommendations or adjust their analyses to align e.g., by including analyses of cancer-specific survival only (and omitting detection); noting the authors similarly argue that cancer detection alone as an endpoint is misleading due to the potential confounding of cardiovascular fitness by health-seeking behaviours.

Response: Thank you for the opportunity to expand on this matter. We agree with the reviewer that focusing solely on diagnostic incidence, or as a composite, is suboptimal, as noted in our original submission. To address this, we separated overall diagnosis from mortality in our original submission, which prior studies have typically not considered.

Despite our large sample size and extended follow-up, relatively few cancer-specific mortality events were observed, limiting our ability to fully address these nuances for specific cancers. While we agree that statistical power alone is not a justification for suboptimal analytical choices, practical constraints of the data sometimes limit the granularity of analysis that can be performed.

Nevertheless, as we principally agree with the reviewer and recognize the value of cancer-specific mortality analyses, we revisited these data. We were able to perform analysis in the total cohort at least, but not the sibling analysis; in the total cohort we achieved model convergence for all cancers except non-melanoma skin and bladder, (two cancers with relatively low mortality before age 65 in Sweden; both with fewer than 300 events out of the 1.1 million conscripts; detailed censoring counts are now available in S4 Table). We now report hazard ratios for these analyses in the supplementary material (S17 Table), which may be valuable for future meta-analyses despite being underpowered. Taking the estimates at face value, despite lower statistical power, the findings support our hypothesis that the excess risks of melanoma and prostate cancer observed in the composite analysis (which heavily favors diagnosis) are likely attributable to bias. This is demonstrated by the reversal of associations for prostate cancer (e.g., Q4 vs. Q1: HR: 0.87, 95% CI: 0.65–1.16) and the attenuation toward unity for melanoma (e.g., Q4 vs. Q1: HR: 1.06, 95% CI: 0.84–1.34) when mortality is examined as the outcome. These results are also more consistent with the findings from our sibling analysis of the composite endpoint.

We have updated the manuscript as follows:

Methods Section:

“Finally, despite few cases (S4 Table), we also computed HRs for site-specific mortality in cohort analysis for 12 of the 14 sites where we observed >300 censoring events (excluding non-melanoma skin and bladder), as these may still be valuable for future meta-analyses.”

Results Section:

“Finally, albeit estimated with greater uncertainty, analysis of cancer-specific mortality in the total cohort yielded similar findings as when using a composite endpoint of diagnosis and deaths for many cancers (S17 Table). The exception was melanoma skin and prostate cancer, for which the excess risks observed in the composite analysis were closer to unity for the former (e.g., top vs bottom quartile: HR: 1.06, 95% CI: 0.84–1.34, *P =* 0.633), and protective for the latter (e.g., top vs bottom quartile: HR: 0.87, 95% CI: 0.65–1.16, *P =* 0.335) (S17 Table).“

And the discussion section to indicate that this analysis strengthens our conclusion, albeit compromised in power:

“However, we provide evidence to suggest that this risk appears to be fully explained by bias as it could not be replicated after accounting for unobserved confounders shared between full siblings. This is further supported by the analysis of prostate-related mortality, where, despite lower statistical power, cardiorespiratory fitness was not associated with an excess risk (e.g., top vs bottom quartile: HR 0.87; 0.65 to 1.16). Unfortunately, the sibling analysis cannot pinpoint the exact confounding factor(s), but it may be a combination of differential health-seeking behaviours (including participation in cancer screening), as well as other shared environmental factors and genetics. “

And revising the limitation point where we previously said we did not complete such analysis:

“Third, due to the limited number of mortality censoring events for site-specific cancers, we were unable to estimate cancer-specific mortality with high confidence; longer follow-up will be necessary to improve the precision of these estimates, and to enable such analyses using sibling comparisons.”

2. Methodological aspects of the authors' presentation of sibling comparison studies seems more optimistic than the literature they cite. For example, they authors say "One possible way to account for unobserved factors is to compare family members, such as siblings;[24] who are assumed to share a large part of these behaviours and other important genetic and environmental confounders.[25,26]". The methodological paper cited ([24]) however says "A popular way to reduce confounding bias is to use sibling comparisons, which implicitly adjust for several factors in the early environment or upbringing without requiring them to be measured or known." Indeed, the assumption that any given potential confounder is sufficiently shared between siblings to provide adequate control in any modelling is particularly important and perhaps not yet recognised sufficiently clearly in the manuscript. Suggest the authors consider moderating their language throughout the manuscript (including the abstract) to reflect that sibling comparison studies account for some, but not necessarily "most" or "all", of potential confounders and including key assumptions/limitations.

Response: Thank you for the opportunity to address this point. We would first like to clarify that we fully recognize sibling comparisons can still be subject to non-shared confounding. While we appreciate the strengths of the method, we do not view it as a ‘silver bullet.’ However, we do believe that sibling comparisons help account for *more* confounding than standard cohort analyses, which is the perspective we aimed to convey in the manuscript.

Upon review, we agree with the reviewer that some parts of the manuscript may come across as overly enthusiastic. We have therefore carefully revised relevant sections to provide a more balanced discussion of the strengths and limitations of the sibling-comparison design. Particular attention has been given to moderating the language in the abstract and early sections of the manuscript (e.g., lines 76-77, 148-149, 480-481).

We have also more clearly stated that the PAFs we estimated could be interpreted as an upper bound for public health benefit:

“Taken at face value, the hypothetically preventable share of cancer mortality at 65 years of age, should everyone from the bottom quartile be moved to the second lowest quartile, was estimated to be 21.4% when comparing unrelated individuals in the population and 14.8% when comparing full siblings. This shift assumed that those with the lowest fitness would increase their fitness level (i.e., W_max_) by 14% on average. However, whether such changes can be achieved through large-scale public health and community interventions, and whether the 14.8% observed in sibling comparisons will hold up under further causal scrutiny, remains to be explored. It seems plausible that this represents an upper bound, as several genetic, environmental, and behavioral confounders may differ even between full siblings.”

3. Relatedly but separately, in the Methods/Statistical Analysis the authors say "For the full-sibling analysis, the aforementioned model was extended to a marginalized between-within model with robust standard errors, which enables control for all unobserved confounders (including genetic and shared environmental factors)." Saying all unobserved confounders are controlled is clearly not correct. Wording similar to that used elsewhere in the manuscript should be used here noting control of all unobserved confounders _shared_between_the_siblings_.

Response: Thank you for highlighting this issue. We have revised the relevant text and conducted a thorough review of the entire manuscript to ensure that the same error does not appear elsewhere.

4. [MINOR] In reference 43 the authors note that traditional Cox regression HR results are very similar to those obtained from their flexible parametric approach. Consider including this point as it potentially reassures readers more familiar with Cox regression than the flexible parametric approach employed.

Response: Thank you for this suggestion. You are correct that the hazard ratios are essentially equivalent between flexible parametric and Cox regression models, as both are estimated using the same assumptions and on the same scales. The primary difference lies in the baseline: Cox regression cancels it, while the flexible parametric model explicitly estimates it – enabling estimation of absolute quantities (e.g., the failure curves).

In response to this comment and a similar remark from another reviewer, we have now repeated our main analysis using Cox regression and included it in the appendix to demonstrate that the results are indeed consistent. This also addresses the needs of readers who may be more familiar with the Cox model. We have updated the sensitivity analysis section to reflect this change:

“We also repeat our analysis using Cox regression, to facilitate comparison between the modelling approaches….”

Additionally, the results section has been revised to acknowledge this addition:

“The hazard ratios were consistent using a Cox regression instead of flexible parametric models; these models rely on the same assumptions so expectation is that they should be consistent.”

We have also clarified in the statistical analysis section that HRs are similar between these models:

These models yield similar HRs as the conventional Cox regression, while having the additional advantage of directly estimating the baseline hazard, thereby enabling the computation of absolute effects measures [42].

5. It's not clear to me how reference 45 relates to this research. It appears to focus on non-survival endpoints and the only reference to survival endpoints (last para of the discussion) refers to results from a separate paper using a fully parametric approach rather than the flexible parametric approach employed by the authors. Apologies if I am missing something here but would appreciate clarification.

Response: Thank you for the opportunity to clarify this point. Sjölander (2021, ref 44) outlines a general approach for constructing a within-estimator. Unlike conditional estimators (e.g., conditional logistic regression or stratified Cox regression), this method incorporates the ‘average’ exposure within clusters (in our case, sibling pairs). While such a ‘between-within’ approach has been previously applied under the conditional between-within framework using random effects (e.g., a random intercept per cluster, see for example: PMID: 23456754, Sjölander (2021) provides a generalization, demonstrating that random intercepts are unnecessary if the goal is to obtain a within-cluster estimate. This approach can be viewed as a ‘marginalized’ version of the conditional between-within framework. It allows for the use of *any* type of model to estimate within-cluster terms by including the average exposure level (i.e., the between terms). Notably, this also has advantages in terms of relaxing certain assumptions, as detailed in ref 25.

While Sjölander 2021 does not specifically address time-to-event analysis (but the same authors did in PMID: 23456754, we believe this is not a necessity. The procedure is independent of the link function or outcome structure and primarily pertains to the framework for specifying the combination of terms required to derive a within-cluster estimate (i.e., using cluster-average fixed-effects and excluding the cluster-level random effects). As such, we can apply Sjölander’s (2021) general procedure to a flexible parametric model with cluster-average terms to transform it into a ‘sibling comparisons analysis’.

We have now added a reference to our prior application of these methods in the time-to-event space:

“The between-within model isolates the individual-level variation (within effect) from the family-level variation (between effect) by including a term for the exposure/covariate and a term for its family average. We have previously employed these methods for time-to-event analysis [27].”

6. Did the authors consider analysing BMI as an effect modifier (i.e., including an interaction between BMI and CV fitness)? Either way, suggest providing some more detail on their decisions regarding how they chose to treat BMI once acknowledged as a "mediator".

Response: Thank you for the opportunity to elaborate on this point and for suggesting an analysis of potential interaction or effect modification between BMI and fitness. In our primary conceptualization, BMI was treated as a confounder in the fitness-cancer relationship, given its assumed role in influencing sedentary behavior (and thereby fitness) and its well-established associations with many cancers. However, recognizing that BMI could also act as a mediator (where lower fitness leads to higher BMI), we included an analysis without adjustment for BMI in the appendix.

Nevertheless, we agree with the reviewer that BMI could theoretically interact with or modify the relationship between fitness and cancer. Yet, our cohort consists primarily of normal weight individuals, as the study population comprises young men conscripted prior to the full onset of current obesity trends. We therefore do not expect significant differences if we were to allow for such interactions, and the within-BMI strata associations are likely underpowered. However, to empirically show this, we have now conducted an additional analysis incorporating product terms between BMI WHO categories (i.e., underweight, normal weight, overweight, and obesity) and fitness quartiles into the flexible parametric model. We then computed marginal hazard ratios for the total population while allowing for interaction/effect modification, in both cohort and sibling analyses. As anticipated, given the predominance of normal weight individuals, the results are consistent with our main findings (see excerpt below):

| Excerpt of S12 Table. | | | | | |
| --- | --- | --- | --- | --- | --- |
| Fully adjusted model assuming no effect modification by BMI, as reported in the main article^a^ | | | Fully adjusted model including interaction terms between cardiorespiratory fitness and BMI^b^ | | |
|  | Cohort analysis  (N=1 124 049) | Sibling analysis  (N=477 453) |  | Cohort analysis  (N=1 124 049) | Sibling analysis  (N=477 453) |
| Quartiles of fitness | HR (95% CI) | HR (95% CI) | Quartiles of fitness | HR (95% CI) | HR (95% CI) |
|  | Overall cancer diagnosis | |  | Overall cancer diagnosis | |
| Q1 | Ref. | Ref. | Q1 | Ref. | Ref. |
| Q2 | 1.01 (0.99, 1.02) | 1.00 (0.96, 1.03) | Q2 | 1.01 (0.99, 1.03) | 1.00 (0.96, 1.04) |
| Q3 | 1.03 (1.01, 1.05) | 1.02 (0.97, 1.06) | Q3 | 1.04 (1.02, 1.06) | 1.03 (0.98, 1.08) |
| Q4 | 1.08 (1.06, 1.11) | 1.00 (0.95, 1.06) | Q4 | 1.10 (1.08, 1.13) | 1.02 (0.97, 1.07) |
|  | Overall cancer mortality | |  | Overall cancer mortality | |
| Q1 | Ref. | Ref. | Q1 | Ref. | Ref. |
| Q2 | 0.83 (0.80, 0.86) | 0.88 (0.81, 0.96) | Q2 | 0.85 (0.81, 0.88) | 0.90 (0.82, 0.98) |
| Q3 | 0.76 (0.73, 0.80) | 0.85 (0.77, 0.95) | Q3 | 0.80 (0.76, 0.84) | 0.86 (0.77, 0.96) |
| Q4 | 0.71 (0.67, 0.76) | 0.78 (0.68, 0.89) | Q4 | 0.76 (0.72, 0.81) | 0.79 (0.69, 0.91) |
| BMI = body mass index. CI = confidence interval. HR = hazard ratio. Q = quartile. Estimates are obtained using flexible parametric survival models, extended to a marginalized between-within model in the sibling cohort.  ^a^Adjusted for age at conscription, year of conscription, BMI (linear and cubed term), parental education, and parental income. ^b^Adjusted for age at conscription, year of conscription, BMI WHO categories, parental education, parental income, *and additionally for interaction terms between cardiorespiratory fitness quartiles and BMI categories.* | | | | | |

Additionally, using the procedure outlined above, we post-estimated hazard ratios within BMI strata, which were largely consistent with the primary results (see S12 Table). However, as expected, the statistical power was limited due to the relatively small number of individuals with overweight or obesity, particularly among those with high cardiorespiratory fitness, in our study population of conscripts.

The statistical description of these analyses has been added to the methods section:

“In addition to adjusting for BMI and excluding it due to potential mediation, we conducted an analysis incorporating World Health Organization BMI categories (i.e., underweight <18.5 kg/m^2^, normal weight 18.5-24.9 kg/m^2^, overweight 25.0-29.9 kg/m^2^, obesity ≥30.0 kg/m^2^), and their interaction terms with cardiorespiratory fitness quartiles, because BMI may effect-modify the relationship between cardiorespiratory fitness and the outcomes. We computed marginal HRs across BMI categories, allowing for interaction, and post-estimated HRs within each BMI category.”

We also briefly describe these findings in the supplementary results section and have added them to the appendix:

“The findings remained largely consistent when accounting for interactions between BMI and cardiorespiratory fitness (S12 Table). However, BMI-strata-specific estimates were limited by low statistical power, as most conscripts were normal weight.”

7. While data missingness leading to subject exclusion from the cohort was objectively low, it is interesting that missing covariate data caused more than double the number of patients to be excluded than missing endpoint data. Suggest the authors provide further information on what variables are driving missingness, whether this introduces any potential biases, and, if so, what was done to minimise the impact. This is particularly important given the small numbers in some of the specific cancer analyses.

Response: Thank you for allowing us to expand on this point. The higher proportion of missing covariate data compared to endpoint data is a natural consequence of the complete (≈100%) coverage of outcomes during follow-up, facilitated by the Swedish nationwide registries. This data source ensures virtually no loss to follow-up (beyond the emigration out of Sweden). However, to provide further clarification of the specific covariates that led to exclude, we have added a flowchart to the supplement that illustrates the distribution of missingness across the specific variables (see S1 Figure).

Furthermore, based on another reviewer’s suggestion, we have now performed a sensitivity analysis using multiple imputations to address the missing covariate data, rather than relying on complete case analysis. This approach resulted in virtually no difference in the estimates (see S13 Table).

8. [MINOR] Ensure confidence intervals are reported consistently across the results (e.g., incidence difference).

Response: Thank you for pointing this out. This oversight has been corrected, and we have carefully reviewed the manuscript to ensure it does not appear elsewhere. Additionally, in accordance with PLOS Medicine reporting standards, we now report p-values in all instances where statistical inferences are made.

9. Suggest saying why smoking was not adjusted for in the analyses. I assume this is because a smoking variable was not available in any of the included datasets but this is worth saying explicitly given how important it is as a modifiable risk factor and, as noted by the authors, would have been particularly useful in the analysis of lung cancer specifically.

Response: We have now added a description of the lack of available data on smoking, and alcohol, as recommended by another review, to the limitations section in the Discussion:

“Fourth, we were unable to adjust our analyses for certain important risk factor, such as smoking and alcohol consumption, which were not available in the databases used”

10. Given the historical and ongoing underrepresentation of women as participants in clinical and observational health research, extreme care should be taken making assertions like this in the discussion "While we may believe there are limited reasons for sex-specific mechanisms linking cardiorespiratory fitness and physical activity to cancer …". At a minimum prior, highly reputable research should be cited and clear arguments made for taking this position.

Response: Thank you for raising this important point. We recognize that our phrasing may have been unnecessarily focused on the mechanism of effect, overlooking the complex issue of underrepresentation. We have now revised the relevant section to more strongly encourage future studies in women, emphasizing that such future studies should also try to use causal methods:

“First, our study was limited to men due to historical conscription practices in Sweden. While some associations could be hypothesized to be similar, we emphasize the importance of replicating our findings in female populations, particularly given sex-specific cancer rates and cancers unique to females. Such replication efforts should prioritize using robust causal methods to address the apparent important role of unobserved confounding.”

11. [MINOR] Notwithstanding the myriad problems associated with the use and interpretation of p-values, I suggest one is included with the point estimate and 95% CI in the very specific case where a result is explicitly said to be "statistically significant" e.g., in the discussion "The association between high adolescent cardiorespiratory fitness and lower risk of overall cancer mortality remained statistically significant…"

Response: Thank you for bringing this to our attention. In the referenced sentence in the discussion, we have now added point estimates and inferential measures. Additionally, in accordance with PLOS Medicine guidelines, we have included p-values *and* 95% confidence intervals wherever statistical inferences are made.

12. [MINOR] The authors and the cited literature very effectively argue against the sole reliance on hazard ratios to characterise exposure-outcome relationships in survival analyses. Consider this advice with respect to the presented abstract.

Response: Thank you for this suggestion. We have carefully revised the abstract to include absolute measures alongside the hazard ratios.

13. [MINOR] Given the relative novelty of some of the methods used, suggest including representative or actual Stata code.

Response: We have uploaded the analytical code to GitHub, generated a unique identifier (https://doi.org/10.5281/zenodo.14673830), and referenced it in the Data Availability section.

14. [MINOR] Ideally tables and figures should be interpretable independently of the main paper. Suggest removing references to "main article" or similar in supplement and, instead, providing sufficient context in the text accompanying the table or figure.

Response: We have carefully reviewed all table and figure legends to ensure they are self-contained, with particular attention to those in the supplemental material.

## Reviewer #2:

This is an interesting study that attempts to examine the relationship between cardiorespiratory fitness measured in adolescence and later life risk of developing cancer.  The data resource employed is novel and the analytic strategy for this type of data seems appropriate.  The main challenge of the paper was the investigation of potential unmeasured confounding using sibling-oriented analysis while measuring few if any risk factors for cancer.

Response: Thank you for acknowledging our study as interesting and novel. We have carefully revised the manuscript in response to your and other reviewers’ comments, and we believe it now provides a more thorough description of both the methodological rigor and the scientific novelty.

Statements that the analysis works by "…controlling for all behavioural, environmental, and genetic confounders that they [siblings] share." is an extraordinarily strong statement with strong assumptions that cannot be tested in this study.

Response: Thank you for allowing us to elaborate on this point. Sibling analysis, or more broadly within-cluster analysis, accounts for *all* factors shared within clusters (families) by design (for a detailed review, see Sjölander 2022 [ref 24 in the original submission], doi: 10.1146/annurev-statistics-040120-024521). This is accomplished by making comparisons within the clusters, assessing how much of the variation in outcomes within clusters can be attributed to differences in exposure within the same clusters. As such, the method inherently adjusts for shared factors (e.g., behavioral, environmental, genetic, and others). Practically, it can be conceptualized as a form of matching among family members.

Importantly, this approach has been widely applied in the causal inference literature across various fields. Some examples include investigations into early risk factors for neurodevelopmental conditions (doi: 10.1017/S0954579420000620), twin comparisons in dementia risk analysis (doi: 10.1093/eurheartj/ehac744), and studies of income inequalities (doi: 10.1257/000282802320191570).

However, as outlined in the discussion, this method depends on several assumptions. To yield an unbiased estimate of the causal effect, we must assume that no other factors contribute to within-family differences (e.g., no measurement error, carry-over effects, or non-shared confounding). While these assumptions are strong and untestable, they are inherent to the methodology and cannot be empirically verified even with extensive data collection. Nonetheless, we believe this method offers superior control for confounding compared to a standard cohort analysis, as it accounts for a substantial proportion of confounders—as many of these are likely to be shared among siblings (e.g., environmental, genetic, and behavioral factors).

It is reasonable to envision the divergence of many behavioral risk factors over 38 years of follow-up and the results of the study point to this as an issue in this study.

Response: We appreciate the opportunity to clarify this matter. While we agree that many risk factors, including fitness, are unlikely to have remained entirely stable over the follow-up period, the focus of our manuscript is specifically on adolescent fitness and its association with later cancer risk. This focus is particularly relevant given the ongoing efforts to promote physical activity and cardiorespiratory fitness among adolescents. Importantly, our study does not address changes in fitness across the lifespan, which represents an interesting but entirely distinct research question.

In addition, our manuscript examines whether the evidence used to inform such adolescent efforts—typically derived from classical cohort studies—provides accurate estimates of potential benefits, or if these estimates differ substantially when analyzed using sibling analysis. In other words, the sibling analysis approach inherently accounts for all unobserved confounders shared within families, and our core objective is to evaluate differences in risk estimates between cohort and sibling analyses for adolescent fitness.

We recognize, however, that the reviewer is suggesting a related but separate research question: to what extent does variation in fitness across the lifespan influence cancer risk? While this falls outside the scope of our study, we share the reviewer’s interest in this topic and have added a discussion of it to the manuscript:

“In our study, we examine adolescent cardiorespiratory fitness, but another question is how variations in fitness across the lifespan influence cancer risk [58]. To our knowledge, no existing studies have explored the role of unobserved confounding in such questions, which we believe would be a valuable area for future research.”

Yet, the authors in this reviewer's reading work to interpret the results in both ways, (a) bias is a problem and (b) early life fitness is associated with lower risk for some cancers. It's a confusing message that does not make an important or adequately strong contribution for publication in a top-tier medical journal.

Response: Thank you for this important comment. First, we want to emphasize that the decision to publish scientific work in a ‘top-tier’ journal should, in our view, be based entirely on its methodological rigor and the scientific advancements it provides—not on the perceived ‘message’ of the findings. Second, our experience with observational studies suggests that results (the ‘message’) are often more nuanced than they initially appear. Simple results are frequently the result of biased methods, which can lead to misleading interpretations. As such, we also believe that complexity—or the lack thereof—should not be a criterion for publication, nor a justification against it.

That said, we recognize the importance of clarity without sacrificing nuance, especially in the abstract. We have revised the conclusion in the abstract to highlight that while adolescent fitness appears to be associated with a lower risk of cancer, standard cohort analyses may introduce biases (e.g., an apparent excess risk of total cancer incidence). Importantly, our findings suggest that some cancers are more strongly linked to fitness, whereas others are more influenced by unobserved confounders and bias. Ultimately, our study underscores the importance of robust study design when investigating the relationship between fitness and subsequent cancer risk if we are to inform public health efforts. The conclusion in the abstract now reads:

“High adolescent cardiorespiratory fitness was associated with lower overall cancer mortality, a finding that persisted in sibling comparisons. However, the influence of unobserved familial confounding appeared to vary by cancer type and be more pronounced for cancer diagnoses than for mortality. This underscores the need for robust causal methods to triangulate results, rather than relying on correlations alone, to better inform public health efforts.”

Additional comments: In the context of risk factors that are not well established, and fitness and cancer risk certainly fall into this category, estimating PAF's may not be appropriate.

Response: Thank you for this suggestion. We generally agree that fitness is an important and novel risk factor for cancer, though we note that the closely related concept of physical activity has received considerably more attention in the literature (and is also recognized by the WHO as an important measure in cancer prevention, as we discuss in the manuscript).

However, given that the primary focus of our paper is the comparison between conventional cohort analysis and sibling analysis—and the differences in estimates between the two—we view the population attributable fractions (PAFs) as an interesting measure of potential public health benefit. The contrast in PAFs across the two analytic approaches provides insight into how public health benefits may be under- or overestimated depending on the method used. We also find the difference in PAFs easier to interpret than changes in, for example, hazard ratios.

That said, we share the reviewer’s concern about the premature interpretation of PAFs in this context and agree with the implicit suggestion that we should provide a more cautious description of these estimates. To address this, we have revised the relevant section in the discussion to include a caveat that there are still several uncertainties that should be addressed before these findings should be translated into public health action:

“Taken at face value, the hypothetically preventable share of cancer mortality at 65 years of age, should everyone from the bottom quartile be moved to the second lowest quartile, was estimated to be 21.4% when comparing unrelated individuals in the population and 14.8% when comparing full siblings. This shift assumed that those with the lowest fitness would increase their fitness level by 14% on average. However, whether such changes can be achieved through large-scale public health and community interventions, and whether the 14.8% observed in sibling comparisons will hold up under further causal scrutiny, remains to be explored. It seems plausible that this represents an upper bound, as several genetic, environmental, and behavioral confounders may differ even between full siblings.”

The authors make no mention of logical biological mechanisms that provide a direct link between cardiorespiratory fitness (a phenotypic response to behavior and genetics) and cancer biology.  The plausibility of any association observed is not well supported here.

Response: Thank you for allowing us to elaborate on this matter. Several proposed mechanisms link adolescent physical fitness with subsequent cancers. We did not initially provide much mechanistic description or hypothesis because our work builds on an extensive existing literature on this topic; as we note in the introduction, there has been a series of individual and meta-studies on this topic. However, we extend this by being the first to use methods that account for unobserved confounders in a large-scale dataset. In other words, we primarily relied on hypotheses set forth by others and aim to provide a more rigorous testing of the existing hypotheses regarding the relationship between adolescent fitness and late adulthood cancers.

Recognizing that not all readers may be as familiar with this existing literature, we have now amended the introduction to include a brief description of the mechanistic hypotheses involved, along with additional references that carefully outline these mechanisms:

“Recently, cardiorespiratory fitness, a robust marker reflecting long-term physical activity and genetic predisposition [3, 4], has also been implicated in cancer risk [5-9]. This has been partly attributed to its moderating effect on the relationship between adiposity and cancer mortality [5], its influence on endogenous sex hormones, metabolic factors, growth factors, and immune-related pathways [10], as well as its potential to reduce the risk of other somatic diseases that may impact cancer prognosis [10].”

This report should find a good home somewhere in the literature, but not sure it's of sufficiently high quality for this journal—too many untestable assumptions.

Response: Thank you for this comment. We are uncertain about the ‘untestable assumptions’ the reviewer is referencing, particularly as our manuscript emphasizes the assumptions inherent in the existing literature. The introduction highlights these assumptions, many of which rely on observational evidence that presumes no alternative explanations for the reported associations. To address this foundational assumption, we employ a novel methodology in this field—sibling comparisons. While this method is robust and well-established in other contexts, it is not without its own assumptions, which we carefully consider in the discussion.

Moreover, much of the existing literature relies heavily on cancer diagnoses, which we argue introduces strong assumptions and may yield spurious findings due to the interplay of true signals and healthcare-seeking behaviors. In contrast, our analysis directly assesses the biases associated with studying incident diagnoses rather than mortality. We specifically focus the discussion on the differences between results based on incidence of diagnosis and those based on mortality.

However, in light of this comment, and with encouragement from other reviewers, we have revised sections of the discussion to more clearly outline the assumptions of sibling analysis, and we have adjusted parts of the conclusion to better reflect these assumptions. Furthermore, keeping in mind the usefulness for future meta-analyses despite compromised power, we now also provide a broader set of estimates for the mortality of specific cancers, as this sheds further light on the assumptions involved in using incident diagnoses for certain cancers.

## Reviewer #3:

This study addresses a timely and important issue, and the authors have taken advantage of a very valuable data source in addressing this issue. A very comprehensive approach to data analysis was undertaken, and the findings provide an important advance in our understanding of the relationship between cardiorespiratory fitness (CRF) during adolescence and cancer risk later in life.  The conclusions are appropriately supported by the findings.  The manuscript is well organized and, for the most part, clearly written.  However, the quality of the manuscript could be enhanced by addressing the following concerns:

Response: Thank you for your endorsement of our work and for the constructive comments. We believe these suggestions have helped to further enhance the quality of our manuscript

1. The phrasing of the Objective in the Abstract is awkward.  The phrase after the comma is not clear.

Response: Thank you for this comment. We have revised the objective, which now reads as follows:

“*Background*: Cardiorespiratory fitness has been linked to both lower and higher risks of cancer, but observational studies may be influenced by unobserved confounders and bias processes. We aimed to examine the associations between adolescent cardiorespiratory fitness and risk of cancer in late adulthood, while attempting to address the unknown influence of unobserved confounders and bias processes.”

2. The purpose statement provided at the end of the Introduction is very long and convoluted.  It would be more effective if it was split into two sentences.

Response: Thank you for the opportunity to revise this. We have now divided the paragraph into two sections and revised it for greater clarity and conciseness:

“In this study, we aim to address the existing inconsistencies and establish a more accurate appreciation for the cancer prevention potential of cardiorespiratory fitness. We conducted a nationwide cohort study over six decades, encompassing over 1.1 million men, to examine the associations between adolescent cardiorespiratory fitness and risk of cancer in late adulthood. To address the influence of unobserved confounders and bias processes, we performed sibling-comparison analyses in nearly 0.5 million full brothers.”

3. In the section on measurement of CRF, the units for expression of Wmax should be provided. Was this expressed relative to body weight?  Also, units should be provided in Table 1.

Response: Thank you for allowing us to elaborate on this. As described in the Methods section, 'W_max_' represents the maximum *wattage* achieved on a maximal ergometer bicycle test with gradually increasing resistance (measured in *watts*). It is an absolute measure and is not relative to body weight; while the starting resistance is relative to body weight, the recorded maximum resistance is recorded as is. However, we account for this in the statistical analysis. Specifically, we adjust for BMI to address the fact that heavier individuals may be likely to generate a higher power output (W_max_) and may also have an increased risk of cancer.

We have now revised the Methods-exposure section to further clarify the definition:

“The test results were recorded as the maximum number of watts of resistance achieved (W_max_), which correlates strongly with maximal oxygen uptake [38].”

Finally, we have included a brief footnote in Table 1 for further clarification:

“^a^W_max_ is the maximum number of watts of resistance achieved on a maximal ergometer bicycle test with gradually increasing resistance”

4.  The first sentence in the Discussion section seems odd and unclear.  This does not seem like an effective way to start the Discussion.  While the point is significant, it does seem to be the major finding of the study.

Response: We appreciate this comment. Typically, we begin the discussion with a sentence summarizing the findings we consider most noteworthy. However, we have now revised the opening to clearly state that high adolescent fitness is associated with lower cancer mortality, followed by a discussion of the more nuanced findings regarding widespread confounding:

“In this nationwide cohort study, we found that higher levels of adolescent cardiorespiratory fitness were associated with lower overall cancer mortality in late adulthood, and this association replicated when comparing full siblings. At the same time, we found evidence suggesting that the associations between adolescent fitness and later-life cancer risk may be easily affected by confounding factors, and there appeared to be varying confounding effects by specific cancer sites.”

5. In the Strengths and Limitations section, the authors have done a good job of noting most of the study's limitations.  However, they fail to mention that, while the sibling analysis is helpful, the data source includes no information on the subjects' physical activity either at baseline or at any point during follow-up.

Response: Thank you for allowing us to elaborate on this point. We investigated cardiorespiratory fitness, which reflects a combination of accumulated strenuous physical activity and genetic predisposition. We believe this is an important area of research in its own right, and has the added advantage of being potentially easier to clinically assess or screen for (e.g., via a maximal exhaustion test) compared to physical activity. Physical activity, often self-reported, suffers from measurement issues, and accelerometers are typically impractical for routine clinical use. Furthermore, we speculate that etiological studies on physical activity may be more strongly affected by behavioral confounders due to its inherently behavioral nature (e.g., participation in cancer screening or engagement in outdoor activities relevant to sun exposure and skin cancer), which may be somewhat mitigated by instead studying the resultant fitness – albeit we provide evidence of the presence of unobserved confounding in cardiorespiratory fitness too.

For these reasons, we view our use of cardiorespiratory fitness as a strength rather than a limitation. That said, we acknowledge that our findings may not perfectly transfer to those from studies focusing on physical activity, and we think it would be valuable to perform similar types of causal analysis of directly measured physical activity (e.g., sibling analysis of accelerometer measured physical activity and long-term cancer mortality). To address this, we have added a paragraph to the discussion noting this distinction:

“Additionally, while our study provides insight into adolescent cardiorespiratory fitness, it would be valuable to apply similar causal approaches to estimate the influence of measured adolescent physical activity on future cancer risk. To our knowledge, existing studies have primarily relied on standard observational analysis [20], with no examination of unobserved confounding in relation to physical activity.”

## Reviewer #4:

It is my pleasure to review this work. The study investigated the associations between adolescent cardiorespiratory fitness and cancer outcomes later in life using a large sibling-controlled cohort of Swedish men who underwent military conscription examinations. Over a median follow-up of 55.9 years, the study found that higher adolescent fitness levels were associated with a reduced risk of overall cancer mortality, as well as decreased risks for several site-specific cancers such as rectum, head and neck, bladder, stomach, pancreas, colon, and others. However, higher risks were observed for prostate and skin cancers, leading to an increased overall cancer diagnosis risk. Sibling comparisons revealed that shared familial confounders accounted for some of these associations, particularly the higher risks for prostate and skin cancers, which attenuated to null in this analysis. The findings underscore the protective effects of high adolescent cardiorespiratory fitness on cancer mortality but highlight the variable role of confounding across cancer types. I have a few suggestions that may improve the manuscript.

Response: Thank you for providing an excellent summary of our paper and conducting a meticulous review of our work.

1. Derivation of study population, the authors have excluded 72,042 individuals due to missing covariate data. The authors should provide the exclusion percentages for each covariate and compare the baseline characteristics between the entire study population and the population after excluding individuals with missing covariates. Moreover, the proportion of missing covariates exceeds 5% (72,042/1,249,131). The authors may consider the use of multiple imputation in the primary analysis and supplement the sensitivity analysis with results from the complete case analysis.

Response: Thank you for these suggestions. Firstly, we have now:

1. Revised the methods section to list the specific covariates that led to exclusions and included the corresponding percentages.
2. Added a participant flow chart, which illustrates the distribution of exclusions for each covariate (see S1 Figure).
3. Provided a descriptive table comparing participants excluded for covariates with those retained for the final analysis (see S2 Table); those excluded were born earlier, conscribed at a slightly older age, and had lower fitness.

Regarding multiple imputation, we suspect that most of the missing data are missing completely at random (MCAR) or possibly missing not at random (MNAR) in a few instances. Therefore, we do not believe that multiple imputation would yield less biased estimates than that of complete case analysis; the former would be less biased under missing at random (MAR). While the proportion of missing covariate data was 5.8%, the decision to use multiple imputation should typically be based on the fraction of missing information (for a detailed discussion, see Madley-Dowd 2019; PMID: 30878639), that the data are MAR (i.e., recoverable conditional on observed covariates), and the presence of strong auxiliary variables. Since we suspect the fraction of missing information is low, the data is likely MCAR or possibly MNAR (and by extension lacks strong auxiliary variables), we chose not to use multiple imputation for our primary analysis.

That said, instead of solely speculating on the results from multiple imputation, we now also perform this as a sensitivity analysis using chained equations with K=20 repetitions. The results are presented in S13 Table, and an excerpt is provided below. The findings are consistent with those obtained using complete case analysis:

| Excerpt of S13 Table. | | | | | | | | | | | |
| --- | --- | --- | --- | --- | --- | --- | --- | --- | --- | --- | --- |
| Cancer Incidence | | | | | | | | | | | |
| Cohort analysis | | | | | | Sibling analysis | | | | | |
|  | Main analysis (complete case) (N=1 124 049) | |  | Multiple imputation using chained equations (N=1 216 782, K=20) | |  | Main analysis (complete case) (N=477 453) | |  | Multiple imputation using chained equations (N=506 096, K=20) | |
|  | HR | 95% CI |  | HR | 95% CI |  | HR | 95% CI |  | HR | 95% CI |
| Quartile 1 | 1.00 | - |  | 1.00 | - | Quartile 1 | 1.00 | - |  | 1.00 | - |
| Quartile 2 | 1.01 | 0.99‐1.02 |  | 1.00 | 0.99‐1.02 | Quartile 2 | 1.00 | 0.96‐1.03 |  | 0.99 | 0.96‐1.03 |
| Quartile 3 | 1.03 | 1.01‐1.05 |  | 1.03 | 1.01‐1.04 | Quartile 3 | 1.02 | 0.97‐1.06 |  | 1.01 | 0.97‐1.05 |
| Quartile 4 | 1.08 | 1.06‐1.11 |  | 1.08 | 1.06‐1.10 | Quartile 4 | 1.00 | 0.95‐1.06 |  | 1.00 | 0.95‐1.05 |
| Cancer Mortality | | | | | | | | | | | |
| Cohort analysis | | | | | | Sibling analysis | | | | | |
|  | Main analysis (complete case) (N=1 124 049) | |  | Multiple imputation using chained equations (N=1 216 782, K=20) | |  | Main analysis (complete case) (N=477 453) | |  | Multiple imputation using chained equations (N=506 096, K=20) | |
|  | HR | 95% CI |  | HR | 95% CI |  | HR | 95% CI |  | HR | 95% CI |
| Quartile 1 | 1.00 | - |  | 1.00 | - | Quartile 1 | 1.00 | - |  | 1.00 | - |
| Quartile 2 | 0.83 | 0.80‐0.86 |  | 0.84 | 0.81‐0.87 | Quartile 2 | 0.88 | 0.81‐0.96 |  | 0.88 | 0.81‐0.95 |
| Quartile 3 | 0.76 | 0.73‐0.80 |  | 0.75 | 0.72‐0.79 | Quartile 3 | 0.85 | 0.77‐0.95 |  | 0.84 | 0.76‐0.92 |
| Quartile 4 | 0.71 | 0.67‐0.76 |  | 0.71 | 0.67‐0.75 | Quartile 4 | 0.78 | 0.68‐0.89 |  | 0.78 | 0.69‐0.89 |
| ^a^The procedure was performed separately for the total cohort and full siblings (using K=20 repetitions), using linear and multinomial logistic models for continuous (BMI) and categorical parameters (quartiles of cardiorespiratory fitness, parental education, and income). Age at conscription, conscription year, follow-up time, and censoring were used as complete auxiliaries, while weight and length at conscription were treated as partially observed auxiliaries (imputed using a linear model). | | | | | | | | | | | |

“Using multiple imputation with chained equations to impute individuals excluded due to missing covariate data yielded results consistent with the main analysis based on complete cases (S13 Table).”

We also briefly describe this in the Sensitivity analyses methods sections:

“In the primary analysis, we excluded conscripts with missing cardiorespiratory fitness and covariates (complete-case analysis), as we suspected the data be missing completely at random or not at random. As an alternative, we applied multiple imputation with chained equations (K=20 repetitions) to retain these individuals. The procedure was performed separately for the total cohort and full siblings, using linear and multinomial logistic models for continuous (BMI) and categorical parameters (quartiles of cardiorespiratory fitness, parental education, and income). Age at conscription, conscription year, follow-up time, and censoring were used as complete auxiliaries, while weight and length at conscription were treated as partially observed auxiliaries (imputed using a linear model).”

2. The authors adjusted for confounders including age at conscription, year of conscription, BMI, parental education, and parental income in the primary analysis. Could the authors clarify the criteria for adjusting for these confounders? Please also include a directed acyclic graph (DAG) to illustrate the relationships between exposure, outcome, and covariates.

Response: Thank you for this important comment. We selected covariates that we assume, based on existing literature and clinical expertise, are causally related to both the exposure and the outcome (i.e., potential confounders). However, we were constrained by a limited set of available covariates, as the data we rely on were collected some time ago, and at that time, only a few registries were available. As a result, much of the information we would consider important today, such as smoking status, was not recorded; we note this as a limitation in the discussion. This also highlights the value of the sibling analysis. To clarify our covariate selection process, we have revised the methods section to read:

“We adjusted for observed covariates which we assumed, based on existing literature and clinical expertise, to be causally related to both the exposure and the outcome (i.e., potential confounders) (S2 Figure).”

We have also added a directed acyclic graph to the supplemental material (see S2 Figure):

“S2 Figure. Directed acyclic graph for the association between adolescent cardiorespiratory fitness and risk of cancer in late adulthood, where observed (white) and unobserved (gray) confounders are illustrated.”

3. The authors used flexible parametric survival models (FPSMs) to estimate hazard ratios. As far as I know, FPSMs rely on selecting an appropriate spline function form and the placement of knots to model the baseline hazard function. The authors selected the 5th, 27.5th, 50th, 72.5th, and 95th percentiles as knot positions. Could the authors clarify how these specific knot positions were chosen?

Response: Thank you for allowing us to clarify this point. FPSMs have the advantage of enabling the estimation of absolute quantities (e.g., the standardized failure function or cumulative incidence) by directly modeling the underlying baseline hazard function. We believe this is important for making the findings clinically and public health relevant, as hazard ratios alone are difficult to interpret. The complicating aspect is the need to choose the spline function, the number of knots (i.e., degrees of freedom), and their placement:

- The shape is nearly always modeled using restricted cubic splines (so-called 'natural splines'), where the shape is allowed to fluctuate cubically between knots but is restricted to be linear outside the boundary knots (to avoid volatility at the tails of the distribution).
- The number of knots is somewhat arbitrary and is typically determined by the complexity of the model that can be fitted to the observed data.
- The placement of the knots is also somewhat arbitrary, though a standard has emerged based on Harrell’s recommendations, which we followed (these are also the default in most existing software). We have now clarified in the statistical analysis section that we rely on Harrell’s recommendations and included the appropriate reference to his book on regression modelling:

“We based the knot placement on Harrell’s recommendations [43].”

Regarding the number of knots, this does not directly affect the hazard ratios, but it could affect the absolute estimates. However, we have often found that this rarely matters as long as a sufficient number of knots is used (e.g., 4 or more). To demonstrate this, we have now included a sensitivity analysis where we use different numbers of knots to estimate the absolute risk differences at age 65 for cancer incidence overall and cancer mortality, all using Harrell’s recommended positions for the knots. As we suspected, the findings are largely unaffected by the number of knots; the estimates for the hazard ratios only changed by single units at the third decimal (see S14 Table), and the absolute risk differences at age 65 were largely consistent (see S15 Table). We now briefly describe this in the sensitivity analysis method section:

“We also repeated our primary analysis (based on 5 knots) varying the number of knots for the baseline hazard between 3 and 7, basing the placement on Harrell’s recommendations [43].”

And the sensitivity analysis result section:

“The findings were consistent when using a different number of knots for the baseline hazard (S14 Table and S15 Table).”

4. When modeling cardiorespiratory fitness using restricted cubic splines (RCS), the knot positions were selected at the 5th, 35th, 65th, and 95th percentiles. What was the rationale behind this selection? The knot positions here differ from those used in the previous analysis. Could the authors explain the relationship between these two sets of knot placements?

Response: Thank you for allowing us to expand on this. To clarify, modeling the baseline hazard is distinct from modeling the relationship between the exposure and the hazard. For example, in some analyses, we model the baseline hazard using 5 knots while modeling the exposure using quartiles. In other analyses, we model both the baseline hazard using 5 knots and the exposure non-linearly using a restricted cubic spline approach with 4 knots. In such instances, and similar to the baseline hazard (see rebuttal to comment 3), the positioning of the knots in the exposure distribution was based on Harrell’s recommendations.

Importantly, Harrell provides different positioning recommendations depending on the number of knots, which ensures they are somewhat evenly distributed across the space. Unlike the baseline hazard, we chose to use only 4 knots rather than 5, as we were more concerned about the power to use a large number of knots in the exposure distribution, particularly in the sibling analysis. In retrospect, we could have harmonized the number of knots between the exposure distribution and the baseline hazard, but we do not believe this would have affected the results much (see results above under comment 3 for findings using 4 knots for the baseline hazard).

As discussed in comment 3, this approach is standard practice when using restricted cubic splines, and the knot positions based on Harrell’s recommendations are the default in most statistical software (e.g., Stata, SAS, and many R packages).

5. FPSMs offer advantages in flexible modeling and estimating absolute effect sizes. I am wondering whether the results from the Cox proportional hazards model are consistent with the current findings. If the results from both models are consistent, the conclusions of the study would be more robust. The authors may include the results from Cox regression in the sensitivity analysis.

Response: The reviewer is absolutely correct about the advantages of the flexible parametric model over Cox regression. As noted by a previous reviewer, it is likely that the hazard ratios obtained from the Cox model will be very similar to those from the flexible parametric model. This is because both models operate on the same scale (the log hazard) and make the same assumptions (e.g., the betas are assumed to be constant across follow-up). Therefore, it would be surprising if they yielded substantially different estimates, with any differences likely stemming from algorithmic variations or the minor influence of additional parameters (such as the baseline splines adding some degrees of freedom to the model but having minimal impact on the individual hazard ratio estimations). For these reasons, we hesitate to agree with the reviewer that consistency would necessarily make the conclusions more robust; essentially, it would be duplicating the same analysis, which does not really enhance the credibility of the conclusions.

That said, for purely statistical curiosity, we now report the hazard ratios from a Cox model in the supplementary material (see S11 Table). An excerpt of these findings is provided below – we show three decimals simply to highlight that the findings rarely differ:

| Excerpt of S11 Table. | | | | | | | | | | | | |
| --- | --- | --- | --- | --- | --- | --- | --- | --- | --- | --- | --- | --- |
| Cancer Incidence | | | | | | | | | | | | |
| Cohort analysis | | | | | |  | Sibling analysis | | | | | |
|  | Flexible parametric model (main analysis) | |  | Cox regression | |  |  | Flexible parametric model (main analysis) | |  | Cox regression | |
|  | HR | 95% CI |  | HR | 95% CI |  |  | HR | 95% CI |  | HR | 95% CI |
| Quartile 1 | 1.000 | - |  | 1.000 | - |  | Quartile 1 | 1.000 | - |  | 1.000 | - |
| Quartile 2 | 1.005 | 0.99‐1.02 |  | 1.005 | 0.989‐1.021 |  | Quartile 2 | 0.995 | 0.960‐1.032 |  | 0.995 | 0.959‐1.032 |
| Quartile 3 | 1.030 | 1.01‐1.05 |  | 1.029 | 1.010‐1.049 |  | Quartile 3 | 1.015 | 0.972‐1.059 |  | 1.013 | 0.971‐1.058 |
| Quartile 4 | 1.082 | 1.06‐1.11 |  | 1.081 | 1.057‐1.105 |  | Quartile 4 | 1.003 | 0.952‐1.056 |  | 1.000 | 0.950‐1.054 |
| Cancer Mortality | | | | | | | | | | | | |
| Cohort analysis | | | | | |  | Sibling analysis | | | | | |
|  | Flexible parametric model (main analysis) | |  | Cox regression | |  |  | Flexible parametric model (main analysis) | |  | Cox regression | |
|  | HR | 95% CI |  | HR | 95% CI |  |  | HR | 95% CI |  | HR | 95% CI |
| Quartile 1 | 1.000 | - |  | 1.000 | - |  | Quartile 1 | 1.000 | - |  | 1.000 | - |
| Quartile 2 | 0.829 | 0.798‐0.861 |  | 0.829 | 0.798‐0.861 |  | Quartile 2 | 0.884 | 0.812‐0.963 |  | 0.884 | 0.812‐0.963 |
| Quartile 3 | 0.762 | 0.728‐0.798 |  | 0.762 | 0.728‐0.798 |  | Quartile 3 | 0.852 | 0.768‐0.945 |  | 0.852 | 0.768‐0.945 |
| Quartile 4 | 0.712 | 0.672‐0.755 |  | 0.712 | 0.672‐0.755 |  | Quartile 4 | 0.781 | 0.684‐0.893 |  | 0.781 | 0.683‐0.893 |

To describe this, we have added the following to the sensitivity analysis section:

“...we repeated our analysis using Cox regression, to facilitate comparison between the modelling approaches.”

And described that it does not differ much in the results section:

“The HRs were consistent using Cox regression instead of flexible parametric models (S11 Table).”

6. Cardiopulmonary health is a reliable indicator of long-term physical activity, and meeting the physical activity levels recommended by the WHO guidelines has a positive effect on cancer mortality and incidence. Therefore, physical activity may be an important confounder in this study, but it seems that the authors did not adjust for it. Full siblings may have different physical activity patterns, how did the authors address this important confounder of physical activity in their analysis?

Response: We appreciate the opportunity to expand on this matter. While we recognize the reviewer’s perspective, we do not necessarily agree that physical activity should be classified as a confounder. This is because we remain uncertain whether physical activity exerts a (non-trivial) causal effect on cancer risk independently of fitness. Instead, we suspect that the observed association between meeting the WHO guidelines for physical activity and reduced cancer risk is primarily attributable to the resulting fitness achieved through sustained high levels of physical activity. In causal graphical terms, physical activity would be considered an ancestor of the exposure rather than a confounder (see the directed acyclic graph provided in response to comment 2). Unfortunately, our dataset does not include information on physical activity, which limits our ability to empirically test this hypothesis. However, one could hypothesize that siblings may share many behaviors and propensities for physical activity, as such behaviors are often shaped by familial and parental influences.

That said, we share the reviewer’s interest in physical activity as an important and related exposure. We agree that studying physical activity as a separate research question—such as examining long-term cancer risk among discordant siblings based on accelerometer-measured physical activity—would be highly valuable. Furthermore, it would be interesting to compare such findings to the results presented in our study; it could be that fitness has a larger magnitude of association by being an isolated marker of the eventual benefit of physical activity, or they share a similar magnitude of association. To address this, we have added a paragraph to the discussion that highlights this point:

“Additionally, while our study provides insight into adolescent cardiorespiratory fitness, it would be valuable to apply similar causal approaches to estimate the influence of measured adolescent physical activity on future cancer risk. To our knowledge, existing studies have primarily relied on standard observational analysis [20], with no examination of unobserved confounding in relation to physical activity.”

7. Among the 14 site-specific cancers in men, pancreatic cancer is likely to be strongly associated with alcohol consumption, while lung cancer is likely to be strongly associated with smoking behavior. I am not sure if I agree the statement "siblings may share smoking patterns" in the limitations section. Smoking and alcohol consumption may be two important confounders in this study, and although they may not be available in the database, are there any alternative variables for smoking and alcohol use, such as smoking disorders and alcohol use disorders?

Response: Thank you for allowing us to expand on this point. First, we would like to emphasize that we agree with the reviewer that different cancers are likely influenced by distinct confounding structures. We believe our findings and manuscript reflect this notion, as some of the variation in attenuation between the cohort and sibling analyses across cancer types likely arises from differences in these underlying confounding structures.

Furthermore, we suspect that siblings share several key risk behaviors, such as smoking and alcohol consumption, to a greater extent than two randomly selected individuals from the general population. This is supported by evidence on the shared genetic and environmental influences on such behaviors. For instance, a meta-analysis of male twins estimated that 37% of the variance in smoking initiation is attributable to additive genetic factors, while 49% is due to shared environmental factors (Li et al., 2002: <https://doi.org/10.1046/j.1360-0443.2003.00295.x>). Similarly, nicotine dependence has been shown to have an even stronger genetic component, with heritability estimates as high as 75% (Vink et al., 2005: <https://doi.org/10.1007/s10519-004-1327-8>; see reference 57 in the original submission).

As we outlined in the original manuscript, we agree that it would have been valuable to directly measure these behaviors and empirically estimate the extent to which the sibling analysis accounts for them, rather than speculate on its ability to address such confounding. Unfortunately, we are unaware of alternative variables that could serve as proxies for these behaviors. For example, later diagnoses of smoking-related disorders and alcohol use disorders are likely substantially underreported in Swedish registries and would often occur well after the exposure assessment, potentially leading to a mediating rather than confounding role.

Nevertheless, while we previously acknowledged that sibling analysis cannot identify specific confounders it controls for, we have now expanded on this limitation in the discussion, using smoking and alcohol consumption as illustrative examples (lines xx-xx):

“Although we were unable to adjust directly for smoking, our sibling analysis likely partly captured smoking behaviour and secondary smoking exposure through control for shared environmental factors, given their large influence on smoking [55]. However, had smoking and other behaviours (e.g., alcohol intake) been directly measured at conscription we could have empirically examined their confounding potential on the relationship between cardiorespiratory fitness and cancer. Nevertheless, these findings also extend those of a previous meta-analysis of four small studies wherein smoking was adjusted for [5].”

8. The authors categorized adolescent cardiorespiratory fitness into quartiles. Could the authors clarify the rationale for this categorization? Are there alternative categorization schemes that might be more clinically or public health relevant?

Response: We appreciate this insightful comment. In the field of physical activity and fitness research, it is common practice to categorize exposures into quartiles, with the lowest quartile serving as the reference group, to illustrate the potential effects of increasing activity or fitness levels across the population. Unfortunately, unlike physical activity, there is no established benchmark for "recommended levels" of fitness to guide a natural clinical or public health categorization (especially, perhaps, because it depends largely on the fitness metric/assessment). Efforts are underway to develop such standards to enable population-wide screening, including ongoing work by members of our team (FB, as part of the World Health Organization working group).

From a statistical perspective, we initially considered using finer categorizations, such as deciles, for our primary analysis. However, given the limited statistical power for some of the rarer site-specific cancers, we ultimately opted for quartiles to provide more robust and stable estimates.

While categorization is a common practice in fitness research, we sought to address the inherent limitations and assumptions of this approach by also conducting a non-linear continuous analysis using restricted cubic splines. This method provides a more detailed and flexible exploration of the exposure-response relationship, avoiding the reliance on arbitrary categorizations and enabling a comprehensive assessment of the associations. However, these results are presented in the appendix and are only briefly discussed in the main text.

9. Maternal factors such as singleton status, low birth weight, and preterm birth may affect adolescent cardiorespiratory fitness. Did the authors consider the influence of these maternal factors on the study outcomes?

Response: Thank you for this interesting suggestion. While we suspect that our sibling comparisons partly account for such factors (given the shared environmental and genetic influences of early-life exposures), we agree that this would be a worthwhile research avenue to pursue.

In previous work, we studied similar questions using a younger cohort of conscripts, exploring the influence of early-life factors such as birth weight (PMID: 32000561) and cesarean delivery (PMID: 33122786) on cardiorespiratory fitness. These analyses revealed associations for birth weight that could not be explained by familial factors, whereas those for cesarean delivery were largely attributable to shared familial influences.

Unfortunately, the Swedish Medical Birth Register, which contains detailed early-life data, does not overlap with the older cohort of males analyzed in the current study. The register only includes some of the most recent conscription cohorts, many of whom are still too young (e.g., around 40 years of age) to have accumulated sufficient cancer outcomes to support independent analysis.

Nevertheless, we recognize the potential of this line of research and have added a note to the discussion highlighting its promise as a future avenue of study:

“An interesting and related question is whether factors present even before adolescence could be incorporated to provide a deeper understanding of the life course trajectory of risk factors. Such studies may become feasible as the youngest individuals in the current cohort—who have data on factors at birth [59, 60]—reach ages where the incidence of somatic diseases is expected to rise,”

10. In comparing Q4 to Q1 for adolescent cardiorespiratory fitness, the authors reported higher risks of prostate and skin cancer, and because these are the most common cancer types, this led to an overall higher risk of cancer diagnosis (HR 1.08; 1.06-1.11). I am also interested in the broader overall cancer diagnosis risk, such as cases with ICD-10 diagnosis codes C00-C97. Could the authors provide results for this broader cancer category?

Response: Thank you for the opportunity to clarify. In our manuscript, we refer to ICD-10 C00-C97 as “overall cancer diagnosis”, and these are the estimates highlighted by the reviewer (HR 1.08; 95% CI: 1.06–1.11).

We are not entirely certain if the reviewer is asking for additional details beyond these estimates. It is possible the reviewer is referring to the abstract, where we noted the higher risk of overall cancer (ICD-10 C00-C97) without including specific numerical estimates. These estimates are, however, outlined in the main text under the Results section and in the accompanying illustrations. To address any potential ambiguity, we have now added these numerical estimates to the abstract for consistency and to avoid further confusion:

“…the highest quartile of fitness had a higher risk of overall cancer diagnosis (HR 1.08; 1.06 to 1.11, *P*<0.001, risk difference 1.32; 0.94 to 1.70 percentage points). “

11. It would be interesting to examine how early-life cardiorespiratory health influences cancer risk and progression over the long term. Could the authors provide the impact of cardiorespiratory fitness on cancer outcomes at different time lags (e.g., 10 years, 20 years)?

Response: We share the reviewer’s interest in this, as is reflected by our original analysis. Specifically, the long-term risk at different time points throughout the follow-up period is reflected in the standardized cumulative incidence reported in Figure 1, with site-specific estimates provided in the Supplement. We also specifically highlight the risk at age 65 (approximately 47 years after conscription assessment) and report the difference in risk, attributable fractions, and competing risk-adjusted risks. We believe that these estimates reflect the ‘cancer risk … over the long term’.

However, in the original submission, we did not report hazard ratios at different time points over the follow-up, as time-specific hazard ratios are generally not interpretable as causal parameters. Specifically, the time-specific hazard ratio is influenced by the depletion of cases over time, as it is conditional on being disease-free up until that time point (as discussed by Hernán, 2010: PMID: 20010207). Nonetheless, we have now included the time-specific hazard ratios for every 5-year interval from age 30 to 65 for all outcomes in the appendix, allowing for the hazard ratios to vary as a function of the follow-up time. While we caution that these are conditional on survival until the respective time point and may not be directly interpretable, we believe they might be useful for future meta-analyses. The estimates are presented in S16 Table, and are described briefly in the results section:

“When the associations were estimated in 5-year intervals, higher fitness was associated with a higher risk of certain cancers at a young age (e.g., age 30), which generally attenuated towards the null in the sibling analysis (S16 Table).”

To the sensitivity analysis section in the Methods section, we added:

“Fifth, we computed time-specific HRs for every 5-year interval from age 30 to 65 for all outcomes, allowing the HRs to vary as a function of the follow-up time.”

12. Please include the information on disease incidence density in the results section.

Response: We appreciate the opportunity to expand on our results section. In the original submission, we only reported the hazard ratios and incidence differences by age 65. In response to the reviewer’s suggestion, we now also report the absolute standardized cumulative incidence by age 65 in the text, as originally reflected in Figure 1. This measure represents the incidence density (1-Survival) that we model. For example, information on disease incidence for overall cancer mortality now reads as follows:

“Compared to the bottom quartile of cardiorespiratory fitness, the HR in the top quartile was 1.08 (1.06 to 1.11, *P*<0.001). The standardised cumulative incidence at 65 years of age was 18.04% (17.78 to 18.30) in the bottom quartile and 19.36% (19.06 to 19.67) in the top quartile, with a difference of 1.32 (0.94 to 1.70) percentage points.”

13. Please add a description of the follow-up start and end points for the siblings.

Response: Thank you for your comment – we might not have been entirely clear on this matter. *All* participants in the study began their follow-up at the date of conscription and were followed until the occurrence of their first outcome, death, emigration, or until December 31, 2023, whichever came first. Additionally, we report the follow-up time, number of events, and number of censored observations in both the cohort and sibling analyses for each specific outcome in the appendix. However, to make it even clearer that this was *the same* in the sibling and cohort analysis, we have now explicitly stated this in the manuscript.

“Participants were followed from the day of conscription until the date of a cancer outcome, emigration, death from non-cancer causes, or end of follow-up (31 December 2023), whichever came first. The sibling analysis was based on the same start of follow-up and end points as the cohort analysis.”

14. Please ensure that the number of decimal places for incidence differences is consistent, for example, 1.58 (1.0 to 1.9) percentage point difference and 0.08 (-0.9 to 1.1).

Response: Thank you for pointing this out. We have now corrected this throughout the manuscript and appendix.

**General editorial requests**

(Note: not all will apply to your paper, but please check each item carefully)

*	We ask every co-author listed on the manuscript to fill in a contributing author statement, making sure to declare all competing interests. If any of the co-authors have not filled in the statement, we will remind them to do so when the paper is revised. If all statements are not completed in a timely fashion this could hold up the re-review process. If new competing interests are declared later in the revision process, this may also hold up the submission. Should there be a problem getting one of your co-authors to fill in a statement we will be in contact. Please do not add or remove authors without first discussing this with the handling editor. You can see our competing interests policy here: http://journals.plos.org/plosmedicine/s/competing-interests

Response: Check.

*	Please upload any figures associated with your paper as individual TIF or EPS files with 300dpi resolution at resubmission; please read our figure guidelines for more information on our requirements: http://journals.plos.org/plosmedicine/s/figures. While revising your submission, please upload your figure files to the PACE digital diagnostic tool, https://pacev2.apexcovantage.com/. PACE helps ensure that figures meet PLOS requirements. To use PACE, you must first register as a user. Then, login and navigate to the UPLOAD tab, where you will find detailed instructions on how to use the tool. If you encounter any issues or have any questions when using PACE, please email us at PLOSMedicine@plos.org.

Response: Check.

*	Please ensure that the paper adheres to the PLOS Data Availability Policy (see http://journals.plos.org/plosmedicine/s/data-availability), which requires that all data underlying the study's findings be provided in a repository or as Supporting Information. For data residing with a third party, authors are required to provide instructions with contact information (web or email address) for obtaining the data. Please note that a study author cannot be the contact person for the data. PLOS journals do not allow statements supported by "data not shown" or "unpublished results." For such statements, authors must provide supporting data or cite public sources that include it.

Response: Check.

*	We expect all researchers with submissions to PLOS in which author-generated code underpins the findings in the manuscript to make all author-generated code available without restrictions upon publication of the work. In cases where code is central to the manuscript, we may require the code to be made available as a condition of publication. Authors are responsible for ensuring that the code is reusable and well documented. Please make any custom code available, either as part of your data deposition or as a supplementary file. Please add a sentence to your data availability statement regarding any code used in the study, e.g. "The code used in the analysis is available from Github [URL] and archived in Zenodo [DOI link]" Please review our guidelines at https://journals.plos.org/plosmedicine/s/materials-software-and-code-sharing and ensure that your code is shared in a way that follows best practice and facilitates reproducibility and reuse. Because Github depositions can be readily changed or deleted, we encourage you to make a permanent DOI'd copy (e.g. in Zenodo) and provide the URL.

Response: Check.

*	FINANCIAL DISCLOSURES: The funding statement should include: specific grant numbers, initials of authors who received each award, URLs to sponsors’ websites. Also, please state whether any sponsors or funders (other than the named authors) played any role in study design, data collection and analysis, the decision to publish, or preparation of the manuscript. If they had no role in the research, include this sentence: “The funders had no role in study design, data collection and analysis, decision to publish, or preparation of the manuscript.”

Response: Check.

*	COMPETING INTERESTS: All authors must declare their relevant competing interests per the PLOS policy, which can be seen here: <https://journals.plos.org/plosmedicine/s/competing-interests> For authors with ties to industry, please indicate whether any of the interests has a financial stake in the results of the current study.

Response: Check.

# **FORMATTING – GENERAL**

*	Abstract: Please structure your abstract using the PLOS Medicine headings (Background, Methods and Findings, Conclusions). Please combine the Methods and Findings sections into one section.

Response: Check.

*	At this stage, we ask that you include a short, non-technical Author Summary of your research to make findings accessible to a wide audience that includes both scientists and non-scientists. The Author Summary should immediately follow the Abstract in your revised manuscript. This text is subject to editorial change and should be distinct from the scientific abstract. Ideally each sub-heading should contain 2-3 single sentence, concise bullet points containing the most salient points from your study. In the final bullet point of 'What Do These Findings Mean?', please include the main limitations of the study in non-technical language. Please see our author guidelines for more information: https://journals.plos.org/plosmedicine/s/revising-your-manuscript#loc-author-summary.

Response: Check.

*	Please express the main results with 95% CIs as well as p values. When reporting p values please report as p<0.001 and where higher as the exact p value p=0.002, for example. Throughout, suggest reporting statistical information as follows to improve clarity for the reader "22% (95% CI [13%,28%]; p</=)". Please be sure to define all numerical values at first use.

Response: Check.

*	Please include page numbers and line numbers in the manuscript file. Use continuous line numbers (do not restart the numbering on each page).

Response: Check.

*	Please cite the reference numbers in square brackets. Citations should precede punctuation.

Response: Check.

FIGURES AND TABLES

*	Please provide titles and legends for all figures and tables (including those in Supporting Information files).

Response: Check.

*	Please define all abbreviations used in each figure/table (including those in Supporting Information files).

Response: Check.

*	Please consider avoiding the use of red and green in order to make your figure more accessible to those with color blindness.

Response: Check

## SUPPLEMENTARY MATERIAL

*	Please note that supplementary material will be posted as supplied by the authors. Therefore, please amend it according to the relevant comments outlined here.

*	Please cite your Supporting Information as outlined here: <https://journals.plos.org/plosmedicine/s/supporting-information>

Response: Check.

REFERENCES

*	PLOS uses the numbered citation (citation-sequence) method and first six authors, et al.

Response: Check.

*	Please ensure that journal name abbreviations match those found in the National Center for Biotechnology Information (NCBI) databases (http://www.ncbi.nlm.nih.gov/nlmcatalog/journals), and are appropriately formatted and capitalised.

Response: Check.

*	Where website addresses are cited, please include the complete URL and specify the date of access (e.g. [accessed: 12/06/2024]).

Response: Check.

*	Please also see https://journals.plos.org/plosmedicine/s/submission-guidelines#loc-references for further details on reference formatting.

Response: Check.

# **STUDY TYPE-SPECIFIC REQUESTS**

*	Abstract: Please include the study design, population and setting, number of participants, years during which the study took place (enrollment and follow up), length of follow up, and main outcome measures.

Response: Check.

*	Please ensure that the study is reported according to the RECORD guideline (available from https://www.record-statement.org) and include the completed checklist as Supporting Information. Please add the following statement, or similar, to the Methods: "This study is reported as per the Reporting of Studies Conducted using Observational Routinely-Collected Data (RECORD) guideline (S1 Checklist)." When completing the checklist, please use section and paragraph numbers, rather than page numbers.

Response: Check.

*	For all observational studies, in the manuscript text, please indicate: (1) the specific hypotheses you intended to test, (2) the analytical methods by which you planned to test them, (3) the analyses you actually performed, and (4) when reported analyses differ from those that were planned, transparent explanations for differences that affect the reliability of the study's results. If a reported analysis was performed based on an interesting but unanticipated pattern in the data, please be clear that the analysis was data driven.

Response: Check.

*	Please state in the Methods section whether the study had a prospective protocol or analysis plan. If a prospective analysis plan (from your funding proposal, IRB or other ethics committee submission, study protocol, or other planning document written before analyzing the data) was used in designing the study, please include the relevant document(s) with your revised manuscript as a Supporting Information file to be published alongside your study and cite it in the Methods section. A legend for this file should be included at the end of your manuscript. If no such document exists, please make sure that the Methods section transparently describes when analyses were planned, and when/why any data-driven changes to analyses took place. Changes in the analysis, including those made in response to peer review comments, should be identified as such in the Methods section of the paper, with rationale.

Response: Check.
