# Supplementary material for: Adolescent cardiorespiratory fitness and risk of cancer in late adulthood: A nationwide sibling-controlled cohort study in Sweden
Source: PLoS Med. 2025 May 8;22(5):e1004597. doi: 10.1371/journal.pmed.1004597 (PMC12061154; doi:10.1371/journal.pmed.1004597)
Supplement: Attachment — Submitted filename: Comments to the Author-PLOS Medicine-2025-01-21.docx [file pmed.1004597.s026.docx]

Although the authors have addressed most of the reviewer’s concerns and presented and explained the relevant results in the manuscript, there are certain points in the response letter with which we disagree. We still have the following issues:

1. For question #1: The authors have addressed our concerns and supplemented the results with those from multiple imputation, which are consistent with the results from complete case analysis. However, the authors suggest that most of the missing data are based on mechanisms of missing completely at random (MCAR) and missing not at random (MNAR). Furthermore, they state that in the case of missing data being missing at random (MAR), multiple imputation would lead to less bias compared to complete case analysis. I am puzzled by the authors' assertion that "most of the missing data are based on mechanisms of MCAR and MNAR." My main concern is: what is the rationale behind the authors' speculation that "most of the missing data are based on MCAR and MNAR mechanisms"?

In the supplementary analysis (Table S2), it is clear that the median year of birth for individuals with complete covariate data is 1966 (1960 to 1971), while for those excluded, the median year of birth is 1956 (1947 to 1977). The difference of 10 years in the median birth year between these two groups raises the question: does the author believe there is no significant difference? This is just an example to illustrate that some covariates may have substantial differences between the groups. This highlights the point that the missing data mechanism—whether MNAR or MAR—cannot be inferred from observed data alone (*BMJ* 2009;338:b2393).

2. For question #3: The authors used flexible parametric survival models (FPSMs) to estimate hazard ratios. However, they argue that this approach is important for making the findings clinically and publicly relevant, as hazard ratios alone are difficult to interpret. This seems somewhat contradictory. Why is it that hazard ratios alone are difficult to interpret? I am also confused about the benefit of estimating the standardized failure function or cumulative incidence using FPSMs for public health significance. Since the authors believe that hazard ratios alone are hard to interpret, for example, shouldn’t they have used restricted mean survival time (RMST) to make the estimates instead? (e.g., Multiply robust estimator for the difference in survival functions using pseudo-observations. *BMC Medical Research Methodology*. 2023. PMID: 37872495)
3. For question #6: We have significant disagreements with the authors regarding the issue of whether physical activity should be considered an important confounder in this study. The authors are uncertain whether physical activity has a (non-trivial) causal effect on cancer risk independent of fitness. However, numerous high-quality observational studies and Mendelian randomization studies have shown that both self-reported and accelerometer-measured physical activity can significantly reduce cancer risk. These studies provide strong evidence supporting physical activity as an independent protective factor. Therefore, we believe that viewing physical activity as solely influencing cancer risk through fitness levels is insufficient.

From both of these perspectives, physical activity not only reduces cancer risk through improving fitness but also acts as an independent exposure factor that likely affects cancer risk through multiple mechanisms. We therefore consider physical activity to be an important confounder in cancer risk.

While we understand that the study was limited by the lack of data on physical activity, which should be addressed in the limitations section. However, we do not agree with the authors' neglect of physical activity as a potential confounder.
