# Supplementary material for: Adolescent cardiorespiratory fitness and risk of cancer in late adulthood: A nationwide sibling-controlled cohort study in Sweden
Source: PLoS Med. 2025 May 8;22(5):e1004597. doi: 10.1371/journal.pmed.1004597 (PMC12061154; doi:10.1371/journal.pmed.1004597)
Supplement: Attachment — Submitted filename: Authors_responses_auresp_3.docx [file pmed.1004597.s028.docx]

# **Comments from the editorial team:**

Please look carefully at the comments provided by the reviewers and the additional input provided by the statistical reviewer. We feel that it is very important to balance and tone down the presentation of conclusions based on potential confounding. We agree with reviewer #4 regarding their comments on physical activity and feel that physical activity should be considered as a confounder. If the data are not available, this should be discussed more clearly as a strong limitation.

**Response: We thank the editorial team for another meticulous review of our revised paper. We have carefully addressed all of the remaining comments from the reviewers. In particular, we have toned down the conclusions, including with respect to the PAF estimates. The Implications section has therefore been carefully revised to provide a more nuanced and cautious interpretation. This includes removing the initial sentence which suggested strong support for initiatives targeting fitness to reduce cancer burden. The section now reads as follows, with revision marked in yellow:**

“The implications of these findings may be considered in the light of the downward trends in population levels of cardiorespiratory fitness, which is observed across all ages, and especially in young people [12-14]. Our findings alone are not sufficient to inform definitive policy statements as they need to be corroborated by further causal analyses. Yet, our findings suggest that the influence of behavioral, environmental, and genetic confounders shared between full siblings might only partly account for the beneficial association between higher fitness and lower cancer mortality. For instance, the hypothetically preventable share of cancer mortality at 65 years of age, should everyone from the bottom quartile be moved to the second lowest quartile, was estimated to be 21.4% when comparing unrelated individuals in the population and 14.8% when comparing full siblings. This shift assumed that those with the lowest fitness would increase their fitness level (i.e., W_max_) by 14% on average. However, whether such changes can be achieved through large-scale public health and community interventions, and whether the 14.8% observed in sibling comparisons will hold up under further causal scrutiny, remains to be explored. It seems plausible that this represents an upper bound, as several genetic, environmental, and behavioral confounders may differ even between full siblings. We want to underscore that the exact point estimates should be very cautiously interpreted given that PAF estimations also rely on strong assumptions. Nevertheless, comparisons of PAF estimates derived from standard cohort analysis versus those from sibling analysis may still be informative with respect to facilitating the clinical interpretation of the degree of influence that shared familial factors may play in the association between fitness and cancer.”

**Moreover, we argue that the role of physical activity as a confounder or not is a highly complex discussion as multiple pathways may be involved. While we primarily view physical activity as a (near) instrumental variable for fitness, meaning that physical activity has a causal effect on fitness and only lowers the risk of cancer via its effect on fitness and not independently, we nevertheless do agree with the reviewers and the editorial team that physical activity could also be postulated to have certain effects that lower the risk of cancer independent of fitness (i.e., it could therefore confound the estimates). However, although many potential mechanisms that may or may not be unique to activity/fitness have been proposed these remain incompletely understood, as highlighted in a recent state-of-the-art review (Kunutsor et al. *GeroScience*, 2024;*46*(6), 5559-5585.) Regardless, we concede that in our previous version of the manuscript we did not emphasize this complexity sufficiently, and we understand that elaboration is warranted. As such, we have revised our DAG to illustrate a potential independent pathway from physical activity to cancer (i.e., it would become a confounder):**

**Moreover, we agree that it would have been optimal if data on physical activity were available, as this would have allowed us to empirically evaluate the role of physical activity as a confounder, rather than having to speculate. A clear statement in this regard was lacking in our previous version of the manuscript, and we have therefore elaborated on this in the Discussion section, in line with the reviewers’ comments:**

“Additionally, a limitation is the lack of data on physical activity, which would have been valuable to explore its potential as a confounder in the association between fitness and cancer. While there have been many suggested mechanisms thought to explain the link between fitness and cancer risk [5,10,11], it is possible that physical activity also has independent links to cancer risk. As outlined in a recent review, however, there is a need for more mechanistic studies on this topic [11]. We welcome future studies to attempt to distinguish between the role of activity versus fitness in cancer risk.”

# **Comments from the reviewers:**

## **Reviewer #1**

Thanks to the authors for their considered responses to my queries. Their clarifications have addressed all my feedback, and the final version of the manuscript is notably strong in how the methodological details are presented. Please accept my congratulations for an excellent paper.

**Response: We are pleased to hear that the extensive work put into our revision satisfied the reviewer’s concern, especially given that the reviewer provided such a constructive and careful review of our paper. We are grateful to the reviewer for recognizing this effort and for helping us strengthen and improve the paper.**

## **Reviewer #2:**

I continue to have limited enthusiasm for this report.

**Response: We appreciate the reviewer’s feedback, although we do not necessarily agree with all of the concerns raised. Nevertheless, we remain confident in the contribution and novelty of our study. Reviewer #1 raised similar concerns and appeared satisfied with our clarifications, which we hope indicates that our explanations were helpful. Below, we have provided further elaboration on the remaining comments and hope the reviewer finds them useful.**

Reviewer 1 and I largely agree on the authors' overly strong assumptions related to confounding problems caused by common cancer risk factors that were not directly examined in this report. Indeed, the authors state in response to my first comment that, "While these assumptions are strong and untestable, they are inherent to the methodology and cannot be empirically verified, even with extensive data collection." It not uncommon for many siblings to diverge in their life course regarding key cancer risk factors such as weight gain, alcohol consumption, smoking, and exercise, which raises questions about the effectiveness of the method used to control for these factors in this analysis.

**Response: Thank you for allowing us to further clarify the rationale behind the sibling-comparison design. First, we want to underscore that we fully agree that risk factors may diverge throughout life, with some likely being more stable than others. However, the rationale behind studying the role of early-life exposures (e.g., fitness) is to better understand the hypothetical benefits of intervening upon such factors in youth, regardless of what happens post-exposure (i.e., later in life).**

**The reviewer seems to suggest that factors differentiating siblings after baseline would somehow invalidate the analysis (i.e., “It [is] not uncommon for many siblings to diverge in their life course regarding key cancer risk factors …”). However, this is not the case, as such changes would either be mediators, colliders, or entirely unrelated to the exposure. Controlling for these factors would either introduce bias or have no impact on the findings. That is, even if we had measured risk factors in siblings across the entire life course, adjusting for them would not be appropriate when trying to study early-life risk factors, as they would either act as mediators or colliders. In other words, only *pre-baseline* factors with strong causal effects on both the exposure and the outcome (i.e., confounders) could bias the results. The sibling analysis accounts for all such shared factors (under certain assumptions that we have outlined), which can be qualitatively argued to provide more valid estimates than comparisons between unrelated individuals (which is reflected in our conclusions).**

**Furthermore, it is important to emphasize that a central aspect of our study—beyond the significance of early-life risk factors—is the comparison between the sibling-analysis and cohort-analysis approaches. Even if the reviewer is particularly concerned with post-baseline factors (despite their inability to introduce bias), it appears unlikely that the divergence in risk profiles over time would be greater between siblings than between unrelated individuals. So, if anything, this further underscores the value of the sibling-analysis approach in studying the long-term implications of early-life fitness (?).**

**In summary, it is inevitable that individuals (whether siblings or unrelated individuals) will change their risk profile over time and may become more or less similar to their peers. However, this will not bias inferences about early-life exposures, as such exposures cannot act as confounders. That said, this could represent an interesting but distinct research question—one we acknowledged in the previous revision (see the section on life-course trajectories and changes: (“In our study, we examined adolescent cardiorespiratory fitness, but another question is how variations in fitness across the lifespan influence cancer risk [59]. To our knowledge, no existing studies have explored the role of unobserved confounding in such questions, which we believe would be a valuable area for future research. An interesting and related question is whether factors present even before adolescence could be incorporated to provide a deeper understanding of the life course trajectory of risk factors. Such studies may become feasible as the youngest individuals in the current cohort – who have data on factors at birth [60, 61] – reach ages where the incidence of somatic diseases is expected to rise. ”).**

In response to my second comment that was also focused on this issue, the authors chose to focus their response on the stability fitness over time vs. divergence of risk factors in siblings that could bias their results.

**Response: We would like to refer to our response to the comment above, underscoring the rationale for the study and the causal question (early-life exposure modification).**

The evidence linking fitness to incident cancer risk remains somewhat limited, and the studies cited (refs 5-9) do not provide strong insight. Refs 5 and 9 appear to be reviews of the same studies, while the study with better control over relevant cancer risk factors (ref 8) found an association with only colon and lung cancer. Furthermore, the latter cancer site is often confounded by smoking due to the strong associations between smoking and lung cancer (i.e., residual confounding resulting from measurement error). Many believe that estimates of PAF assume that risk factors have been convincingly linked to the outcome of interest, and using this metric to describe the public health impact of changes in behaviors with more limited evidence is not advisable.

**Response: We fully agree that further evidence is needed to clarify the relationship between fitness and cancer risk, particularly through studies employing robust causal inference methods. In this regard, we believe our study makes a valuable contribution by addressing a critical gap in the existing literature—namely, the application of methods designed to control for unobserved confounders and bias. The importance of such approaches in assessing causality was recently emphasized in a state-of-the-art review of this topic (Kunutsor et al., GeroScience, 2024; 46(6), 5559–5585).**

**Regarding the use of PAFs, we acknowledge the reviewer’s concern that these estimates should be interpreted with caution and do not necessarily reflect the direct public health impact of interventions targeting fitness. However, we respectfully disagree that PAFs should be removed from the manuscript. These estimates serve as a standardized rescaling of our model outputs and do not introduce additional assumptions beyond those inherent in hazard ratios or absolute risk measures.**

**As previously stated, our primary reason for including PAF estimates was not to quantify the prevention potential of increasing fitness levels. Rather, our intent was to enhance the interpretability of our findings by illustrating the impact of adjusting for familial confounders in comparison to standard cohort analyses. Hazard ratios alone, while informative, have limited clinical utility, and shifts in their magnitude across analytic approaches can be difficult to contextualize. By incorporating PAFs, we provide a clearer comparative framework between cohort-based and sibling-based estimates, thereby increasing the public health relevance of the attenuation we observe once adjusting for all unobserved factors shared between siblings (e.g., genetics, environment, behaviors).**

**That said, we appreciate the reviewer’s perspective and recognize the need for cautious interpretation of PAFs in this context. In response, we have again revised the discussion to ensure that our conclusions remain appropriately measured. Specifically, we have refined the implications section, including the removal of the initial sentence that suggested direct support for fitness-based interventions as a means of reducing cancer burden. The revised section now reads as follows:**

“The implications of these findings may be considered in the light of the downward trends in population levels of cardiorespiratory fitness, which is observed across all ages, and especially in young people [12-14]. Our findings alone are not sufficient to inform definitive policy statements as they need to be corroborated by further causal analyses. Yet, our findings suggest that the influence of behavioral, environmental, and genetic confounders shared between full siblings might only partly account for the beneficial association between higher fitness and lower cancer mortality. For instance, the hypothetically preventable share of cancer mortality at 65 years of age, should everyone from the bottom quartile be moved to the second lowest quartile, was estimated to be 21.4% when comparing unrelated individuals in the population and 14.8% when comparing full siblings. This shift assumed that those with the lowest fitness would increase their fitness level (i.e., W_max_) by 14% on average. However, whether such changes can be achieved through large-scale public health and community interventions, and whether the 14.8% observed in sibling comparisons will hold up under further causal scrutiny, remains to be explored. It seems plausible that this represents an upper bound, as several genetic, environmental, and behavioral confounders may differ even between full siblings. We want to underscore that the exact point estimates should be very cautiously interpreted given that PAF estimations also rely on strong assumptions, and our estimates in particular were based on prevalences dating back to the 1970s. Nevertheless, comparisons of PAF estimates derived from standard cohort analysis versus those from sibling analysis may still be informative with respect to facilitating the clinical interpretation of the degree of influence that shared familial factors may play in the association between fitness and cancer.”

**Accordingly, we have clarified in the methods section the rationale for including PAFs in the study:**

**“**To further aid the interpretation of potential changes in estimates from cohort analysis to sibling-analysis, we estimated population-attributable fractions (PAF)…”

Last, the authors provided no plausible mechanism linking fitness (not activity) to biological pathways that may reduce cancer risk. They seem to suggest that activity increases fitness, and the mechanisms related to activity may also be operative for fitness. Perhaps providing evidence of mechanisms linking fitness to cancer in the context of a causal diagram that provides evidence for the direct effects of fitness on biological mechanisms related to cancer independent of physical activity would be useful. If the mechanisms are activated by exercise/activity and fitness is just a bystander in relation to cancer risk, what does that say about the potential for a putative causal effect of fitness on cancer risk?

**Response: We agree that much remains to be understood regarding the mechanisms linking fitness with cancer. As outlined in the introduction in response to the reviewer's comment in the previous round, numerous mechanisms have been proposed to explain this association:**

**“Recently, cardiorespiratory fitness, a robust marker reflecting long-term physical activity and genetic predisposition (3, 4), has also been implicated in cancer risk (5-9). This has been partly attributed to its moderating effect on the relationship between adiposity and cancer mortality (5), its influence on endogenous sex hormones, metabolic factors, growth factors, immune-related pathways (10), inflammation reduction (11), as well as its potential to reduce the risk of other somatic diseases that may impact cancer prognosis (10).”**

**While we share the reviewer’s interest in mechanistic research, we believe that extensively speculating on potential pathways falls beyond the scope of our study, given that we do not provide direct empirical mechanistic evidence. As described in the introduction, our primary focus is on addressing the methodological challenges inherent in existing observational studies in this field and advancing the research through improved confounder control.**

**That said, we *strongly* encourage further research to explore these mechanistic pathways in greater detail. In particular, future studies could investigate whether physical activity exerts an independent effect beyond its established benefits on fitness. To reflect this perspective, we have now expanded on this discussion in the revised manuscript:**

“Additionally, a limitation is the lack of data on physical activity, which would have been valuable to explore its potential as a confounder in the association between fitness and cancer. While there have been many suggested mechanisms thought to explain the link between fitness and cancer risk [5,10,11], it is possible that physical activity also has independent links to cancer risk. As outlined in a recent review, however, there is a need for more mechanistic studies on this topic [11]. We welcome future studies to attempt to distinguish between the role of activity versus fitness in cancer risk.”

## **Reviewer #4:**

Although the authors have addressed most of the reviewer's concerns and presented and explained the relevant results in the manuscript, there are certain points in the response letter with which we disagree. We still have the following issues:
1. For question #1: The authors have addressed our concerns and supplemented the results with those from multiple imputation, which are consistent with the results from complete case analysis. However, the authors suggest that most of the missing data are based on mechanisms of missing completely at random (MCAR) and missing not at random (MNAR). Furthermore, they state that in the case of missing data being missing at random (MAR), multiple imputation would lead to less bias compared to complete case analysis. I am puzzled by the authors' assertion that "most of the missing data are based on mechanisms of MCAR and MNAR." My main concern is: what is the rationale behind the authors' speculation that "most of the missing data are based on MCAR and MNAR mechanisms"?

**Response: Thank you for the opportunity to elaborate on this matter. Our characterization of the missing data as predominantly MCAR, with some instances of MNAR, is based on our extensive experience working with this data in various contexts – albeit it is admittedly speculation on our behalf. Concerning MCAR, some missing data have been lost due to archival issues—one notable example being a fire in an archival unit in 1982, which resulted in data loss. Many of these factors can be reasonably assumed to be random. Concerning MNAR, this aligns with our description in the Methods section, where we note that individuals with care needs or disabilities were typically exempt from conscription. While such individuals should not have been called for conscription, in practice, some may have been called and undergone initial assessment before it became evident that they were ineligible (e.g., due to a clear disability or previously undetected congenital conditions such as heart malformations). Although these mechanisms of missingness—whether random or non-random—cannot be empirically confirmed, our assessment is informed by extensive experience curating these data and consultations with the Swedish Armed Forces.**

**That said, as also noted by the Statistical Reviewer, we have conducted analyses using both complete case and multiple imputation approaches. In total, these should provide robust estimates under a range of missing data mechanisms (regardless of whether it is MAR, MCAR, or MNAR).**

In the supplementary analysis (Table S2), it is clear that the median year of birth for individuals with complete covariate data is 1966 (1960 to 1971), while for those excluded, the median year of birth is 1956 (1947 to 1977). The difference of 10 years in the median birth year between these two groups raises the question: does the author believe there is no significant difference? This is just an example to illustrate that some covariates may have substantial differences between the groups. This highlights the point that the missing data mechanism—whether MNAR or MAR—cannot be inferred from observed data alone (BMJ 2009;338:b2393).

**Response: We appreciate the opportunity to clarify this. In Table S2, we only showcased data for the analytical sample and for those excluded due to missing covariate data. We recognize that this was not sufficiently detailed. To improve clarify, we have revised Table S2 to showcase the data after stepwise exclusions, beginning with showcasing all available data from the underlying sources, including individuals who were initially excluded for conscribing before 1972 and after 1995, which were originally omitted due to inconsistencies in data collection. Additionally, the early years of data were affected by poor storage of weight records, which were part of the complete-covariate requirement. These factors collectively explain the differences in median birth years between the columns. We have also added available data on BMI to the table. Nevertheless, as also echoed by the Statistical reviewer, the missing data is relatively small.**

2. For question #3: The authors used flexible parametric survival models (FPSMs) to estimate hazard ratios. However, they argue that this approach is important for making the findings clinically and publicly relevant, as hazard ratios alone are difficult to interpret. This seems somewhat contradictory. Why is it that hazard ratios alone are difficult to interpret? I am also confused about the benefit of estimating the standardized failure function or cumulative incidence using FPSMs for public health significance. Since the authors believe that hazard ratios alone are hard to interpret, for example, shouldn't they have used restricted mean survival time (RMST) to make the estimates instead? (e.g., Multiply robust estimator for the difference in survival functions using pseudo-observations. BMC Medical Research Methodology. 2023. PMID: 37872495)

**Response: We appreciate the opportunity to clarify this matter. It is well recognized that hazard ratios can be challenging to interpret—not only because they represent a relative measure that does not convey absolute risk (as is also the case for relative risks and odds ratios) but also because they are a weighted average over the follow-up period and are therefore inherently subject to selection bias (see Hernán, *The Hazard of Hazard Ratios*, PMID: 20010207). This is also echoed by the Statistical Reviewer (see below).**

**That said, hazard ratios remain the standard measure in survival analysis, and we consider it necessary to report them. However, to address their limitations, we also present absolute cumulative risks. These provide a more clinically informative measure of risk and enhance the interpretability of our findings; there are many clinical measures (e.g., RMST) that could also have been estimated, but we chose the absolute cumulate risk (failure) simply because we find it the most intuitive of available measures.**

3. For question #6: We have significant disagreements with the authors regarding the issue of whether physical activity should be considered an important confounder in this study. The authors are uncertain whether physical activity has a (non-trivial) causal effect on cancer risk independent of fitness. However, numerous high-quality observational studies and Mendelian randomization studies have shown that both self-reported and accelerometer-measured physical activity can significantly reduce cancer risk. These studies provide strong evidence supporting physical activity as an independent protective factor. Therefore, we believe that viewing physical activity as solely influencing cancer risk through fitness levels is insufficient.

From both of these perspectives, physical activity not only reduces cancer risk through improving fitness but also acts as an independent exposure factor that likely affects cancer risk through multiple mechanisms. We therefore consider physical activity to be an important confounder in cancer risk.

While we understand that the study was limited by the lack of data on physical activity, which should be addressed in the limitations section. However, we do not agree with the authors' neglect of physical activity as a potential confounder.

**Response: We thank the reviewer for the opportunity to elaborate on the role of physical activity. We agree that determining whether physical activity serves as a confounder is inherently complex, given the multiple biological and behavioral pathways involved. We speculated that physical activity may function as a (near) instrumental variable for fitness—meaning that physical activity exerts a causal effect on fitness, which in turn lowers cancer risk, and that physical activity itself is not directly linked to cancer risk.**

**It is also in this light that we interpret the studies referenced by the reviewer—both classic observational studies and Mendelian Randomization studies. We *believe* that the observed association between physical activity and cancer risk in these studies primarily reflects the effect of physical activity on fitness, which subsequently confers protection against somatic diseases. To our knowledge, none of these studies—whether observational or Mendelian Randomization—have provided evidence that physical activity is directly linked to cancer risk *independent* of its effect on fitness (i.e., none of them, as far as we are aware, have tried to parse out the mediation via fitness).**

**That said, we recognize that this remains speculative in the absence of direct data, and we appreciate the reviewer’s emphasis on the need for a more nuanced discussion of this complexity. We also agree that access to physical activity data would have strengthened our ability to empirically evaluate its role as a confounder. However, such data are not collected in any nationwide registry in Sweden, limiting our ability to formally assess this issue. This limitation and nuance was not sufficiently articulated in the previous version of the manuscript, and we have now expanded on this point in the Discussion section:**

“Additionally, a limitation is the lack of data on physical activity, which would have been valuable to explore its potential as a confounder in the association between fitness and cancer. While there have been many suggested mechanisms thought to explain the link between fitness and cancer risk [5,10,11], it is possible that physical activity also has independent links to cancer risk. As outlined in a recent review, however, there is a need for more mechanistic studies on this topic [11]. We welcome future studies to attempt to distinguish between the role of activity versus fitness in cancer risk.”

## **Additional comments from the statistical reviewer:**

Point 1: The reviewer seems to accept the newly provided results that complement the full case analysis with MI for the relatively small amount of missing data. They seem to have concerns about the reasoning behind the missingness mechanisms proposed by the author. While I agree that it would be interesting to hear more of the authors' reasoning for why they think the data are MCAR and MNAR, it doesn't ultimately make any difference to the analyses underlying the current research for two reasons. Firstly, the amount of missing data is very small, so the likelihood of the missing data having a significant impact on the conclusions of this research is minimal. Secondly, and with this in mind, the authors have already presented results using CCA and MI to deal with missingness and have shown comparable results. MI is valid for MCAR and MAR, but may be biased for MNAR if CCA should be considered (https://academic.oup.com/ije/article/48/4/1294/5382162). By presenting both CCA and MI results, the authors have adequately covered their bases.

**Response: Thank you for highlighting that our presentation of both complete case analysis (CCA) and multiple imputation (MI), along with the limited extent of missing data, sufficiently addresses concerns about its potential impact on our conclusions. Regarding the mechanisms of missingness, we have provided further elaboration to the reviewer above. However, as this remains largely speculative, we have opted not to include additional details on this matter in the manuscript.**

Point 2: The reviewer makes a similar point to my own review point:

"""12. [MINOR] The authors and the cited literature very effectively argue against the sole reliance on hazard ratios to characterise exposure-outcome relationships in survival analyses. Consider this advice with respect to the presented abstract.

Response: Thank you for this suggestion. We have carefully revised the abstract to include absolute measures alongside the hazard ratios."""

The interpretability of hazard ratios is a well-documented challenge, but they are also the main statistic for survival/time to event analyses and this is not likely to change in the near future. With this reality in mind, I think the authors have done a good job of walking the fine line between raising the issues with hazard ratios, but also presenting them for comparability with other research in addition to their absolute effect estimates made available by using FPSMs, i.e. "These [FSPM] models yield similar HRs as conventional Cox regression, while having the additional advantage of directly estimating the baseline hazard, thereby enabling the computation of absolute effects measures" (see lines 234 to 237 in the latest submission). I think this fully addresses the reviewer's point and obviates the need for other statistics such as RMST.

**Response: We thank the statistical reviewer for their encouraging words and are pleased to hear that we have successfully balanced the discussion of the limitations of hazard ratios while still presenting them for comparability with other research, alongside absolute measures.**
