# Supplementary material for: Adolescent cardiorespiratory fitness and risk of cancer in late adulthood: A nationwide sibling-controlled cohort study in Sweden
Source: PLoS Med. 2025 May 8;22(5):e1004597. doi: 10.1371/journal.pmed.1004597 (PMC12061154; doi:10.1371/journal.pmed.1004597)
Supplement: Attachment — Submitted filename: Authors_responses_auresp_4.docx [file pmed.1004597.s029.docx]

# **Authors’ responses**

**Comments from Reviewers:**

Reviewer #1: Thank you for the opportunity to review the ongoing discussion regarding this manuscript. I remain supportive of its acceptance for publication.

- **Response:** Thank you, we are happy for the support and care taken during the review.

Reviewer #4: The authors have effectively addressed our initial concerns and made appropriate revisions to the paper. Congratulations on the great work.

- **Response:** We are happy that the reviewer is satisfied with our revision and for the endorsement, thank you.

**Requests from Editors:**

GENERAL

* Please ensure that all abbreviations are defined at first use throughout the text (including statistical abbreviations). Please also check figures and tables.

- **Response:** We have now checked and ensured the definition of abbreviations at first use.

* Please review your text for claims of novelty or primacy (e.g. 'for the first time', ‘novel’) and remove this language.

- **Response:** We have reviewed the text accordingly and did not identify any claims of novelty or primacy.

* Please check that any use of statistical terms (such as trend or significant) are supported by the data, and if not please remove them.

- **Response:** We have checked this and confirm that mentioning of statistical terms are always supported by the data.

* Please ensure that tables and figures, including those in supplementary files, are appropriately referenced in the main text.

- **Response:** We have ensured that all tables and figures, including supplementary files, are appropriately referenced.

* Statistical reporting: Please revise throughout the manuscript, including tables and figures (including Supplementary Materials).
a) Please report statistical information as follows to improve clarity for the reader "22% (95% CI [13%,28%]; p</=)".
b) Please separate upper and lower bounds with commas instead of hyphens as the latter can be confused with reporting of negative values.
c) Please define statistical definitions at first use and repeat the abbreviated definitions (HR, CI etc.) for each set of parentheses.

- **Response:** All of the above have been revised throughout.

* The link in the data availability statement leads to a page that says "The page could not be found". Please replace the link and be sure to provide a link that provides a clear point of contact for researchers interested in the data.

- **Response:** We have replaced the link ensuring it works and has a clear point of contact for researchers interested in the data:

* Financial Disclosure: Please ensure that the details provided on page 29 are included in the relevant statement in the metadata in the online submission form.

- **Response:** Confirmed.

* Competing Interest: Please ensure that the details provided on page 29 are included in the relevant statement in the metadata in the online submission form.

- **Response:** Confirmed.

* Title: We suggest adding 'in Sweden' (or similar) to the title.

- **Response:** We have now added “in Sweden” to the title.

ABSTRACT

* Please confirm that your abstract complies with our requirements, including providing all the information relevant to this study type<https://journals.plos.org/plosmedicine/s/submission-guidelines#loc-abstract>

- **Response:** Confirmed.

* l.54ff: Please clarify what "they" refers to (the highest quartile of fitness?). For clarity, we suggest changing the text in the preceding lines to: "In the cohort analysis, individuals in the highest quartile of fitness...".

- **Response:** We have changed the text according to this suggestion.

* Please ensure that all numbers presented in the abstract are present and identical to numbers presented in the main manuscript text.

- **Response:** Confirmed.

* l.60ff: We suggest changing to: Individuals in the highest quartile of fitness had a higher risk of overall cancer diagnosis (HR 1.08; 1.06 to 1.11, P<0.001, incidence difference 1.32; 0.94 to 1.70 percentage points), results driven by prostate and skin cancer being the most common types of cancer.

- **Response:** We have changed according to the suggestion.

* l.64: We suggest removing the word "all" and simply stating "thereby controlling for unobserved shared confounders."

- **Response:** We have changed according to the suggestion.

* In the Abstract Conclusion, first sentence, please make sure it is clear that the association refers to overall cancer mortality in late adulthood.

- **Response:** We have changed according to the suggestion.

AUTHOR SUMMARY

* We suggest re-phrasing the bullet points under ‘Why Was This Study Done?’:
   1) High cardiorespiratory fitness, a measure of regular physical activity and linked to genetics, has been associated with reduced cancer risk.
   2) Improving fitness in young people is a growing public health focus.
   3) Existing studies may have limitations, such as not fully accounting for other factors, such as participation in cancer screening and other health behaviors that may be more common in individuals with higher fitness levels.

- **Response:** We have rephrased the bullets according to all these suggestions.

* Please change ‘The researchers’ and ‘They’ to ‘We’.

- **Response:** Done.

* Please specify ‘behaviors’.

- **Response:** We have specified these behaviors:

  “This link remained even after accounting for factors shared between siblings, such as genetics, shared environment, **and health-seeking behaviors such as participation in cancer screening.”**

* Please clarify that you are referring to cardiorespiratory fitness on line 103.

- **Response:** Done.

* In the final bullet point of 'What Do These Findings Mean?', please include the main limitations of the study in non-technical language.

- **Response:** We have revised the final bullet to highlight limitations:

  **”Our study did not include female participants, was not able to account for certain relevant risk factors for cancer such as smoking, alcohol, and physical activity, and only accounted for the unobserved confounders that are shared between full siblings. These limitations should be addressed in future studies to expand upon our results.”**

INTRODUCTION

* l.122: Similar to the Author Summary, we feel that the statement that cardiorespiratory fitness is a robust marker reflecting genetic predisposition, might be misleading. We suggest re-phrasing to make clear that genetic factors may influence cardiorespiratory fitness.

- **Response:** We have rephrased to:

  “Recently, cardiorespiratory fitness, a **marker influenced by long-term physical activity and genetic factors** [3,4], has also been implicated in cancer risk [5-9].”

* l.151: We suggest removing "all" because it is unlikely that you can guarantee that you have in fact covered all previous studies.

- **Response:** Done.

* l.152: Are you 100% sure there is only one? We suggest adding a qualifier, such as "to the best of our knowledge".

- **Response:** We added the suggested phrase.

METHODS AND RESULTS

* If you have not already, please provide an explanation why liver, bile ducts, and gallbladder cancer were grouped as one.

- **Response:** Liver, bile ducts, and gallbladder were collapsed to one group to align and enhance comparability with a recent (ref 6), but also to increase statistical power. We have added a justifying statement in the paper:

  “To this, we also perform an analysis of 14 site-specific cancers (either diagnosis or death), which were selected based on their previously identified associations with physical activity or cardiorespiratory fitness:[2, 5, 6, 21] head and neck, oesophagus, lung, stomach, pancreas, colon, rectum, kidney, prostate, bladder, myeloma, skin (divided into melanoma and non-melanoma), and liver, bile ducts, and gallbladder **(enhancing comparability with a previous study [6]).”**

* l.342ff: For the full sibling analysis, please ensure that you discuss all site-specific cancers for which you observed attenuation. We suggest removing the word ‘tendencies’ as you clearly observed attenuation. We also noted that you did not discuss the results for myeloma at all.

- **Response:** We have now ensured that all site-specific cancers that attenuated are mentioned, and we removed the word tendencies. The revised paragraph reads:

“In full-sibling analysis, the degree of change in the associations, as compared to in the total cohort, varied between cancer types (Figure 2, S3 Figure, S4 Figure, S5 Figure, S5 Table, S6 Table). The excess risk of non-melanoma skin cancer was largely attenuated (HR: 1.09, 95% CI [0.99, 1.20]; *P* = 0.097, incidence difference: 0.40 percentage points, 95% CI [-0.07, 0.87]), as was the risk of prostate cancer (HR: 1.01, 95% CI [0.90, 1.13]; *P* = 0.867, incidence difference: 0.05 percentage points, 95% CI [-0.50, 0.60]). **Attenuation was observed also for melanoma skin cancer (from HR: 1.50, 95% CI [1.41, 1.61] to HR: 1.30, 95% CI [1.10, 1.52]), liver, bile ducts, and gallbladder cancer (from HR: 0.59, 95% CI [0.50, 0.69] to HR: 0.78, 95% CI [0.54, 1.13]), kidney cancer (from HR: 0.65, 95% CI [0.57, 0.74] to HR: 0.86, 95% CI [0.63, 1.18]), stomach cancer (from HR: 0.70, 95% CI [0.58, 0.86] to HR: 0.91, 95% CI [0.60, 1.37]), bladder cancer (from HR: 0.74, 95% CI [0.65, 0.85] to HR: 0.88, 95% CI [0.66, 1.19]), and pancreas cancer (from HR: 0.73, 95% CI [0.62, 0.85] to HR: 0.83, 95% CI [0.59, 1.18]),** translating into reduced PAFs (S7 Table). **For lung cancer, there was no attenuation (cohort HR: 0.49, 95% CI [0.42, 0.57], sibling HR: 0.50, 95% CI [0.36, 0.70], and for colon, head and neck, and oesophagus cancer, the HRs were also similar, albeit associated with larger uncertainty and not necessarily statistically significant (S5 Table). The estimates for myeloma were not statistically significant either in cohort or sibling analysis (S5 Table).”**

* l.360: “In contrast, associations with colon and head and neck cancer seemed more robust.” – please explain in more detail. It seems that for head and neck cancer the statement is not true. Please revise.

- **Response:** We assume this comment relates to the P values not being statistically significant in the sibling-analysis. We agree these associations are somewhat challenging to interpret considering the larger uncertainty, and have revised the text to read:

**”For colon, head and neck, and oesophagus cancer, the HRs were similar, albeit associated with larger uncertainty and not necessarily statistically significant.”**

* Table 1: Is it correct that the cardiorespiratory fitness and Wmax values by quartile are exactly the same between the total cohort and the full sibling cohort?

- **Response:** Yes, it is correct.

* Figure 1 and 2: Please define ‘CI’.

- **Response:** Check.

* Figure S2: Figures cannot be reproduced from other sources that are not CC-BY. Please provide a different figure.

- **Response:** Figure S1 & S2 were generated using Biorender and has an approved CC-BY 4.0 license. We have now added this to the relevant figure legend and acknowledgement and separately uploaded the licenses for archival.

DISCUSSION

* As a general reminder, please be sure to acknowledge any limitations the reviewers have pointed out, such as lack of insight into biological mechanisms. Also, please ensure that the language throughout the Discussion is measured, i.e. that you do not overstate any of the findings and results, and that you interpret the results with caution based on the limitations.

- **Response:** We have carefully reviewed the manuscript and we believe we acknowledge all limitations pointed out by reviewers, including lack of insight into biological mechanisms (e.g., in the discussion: “Additionally, a limitation is the lack of data on physical activity, which would have been valuable to explore its potential as a confounder in the association between fitness and cancer. While there have been many suggested mechanisms thought to explain the link between fitness and cancer risk [5, 10, 11], it is possible that physical activity also has independent links to cancer risk. As outlined in a recent review, however, there is a need for more mechanistic studies on this topic [11]. We welcome future studies to attempt to distinguish between the role of activity versus fitness in cancer risk”).

* Please remove any subheadings in the Discussion, including the Conclusion subheading.

- **Response:** Check.

* l.437: Please explain what you mean by heterogeneous pattern. We believe that sibling analysis, with a few exceptions, mostly leads to attenuation of the results, i.e. we are not sure that heterogeneous pattern is an appropriate description here.

- **Response:** We agree that using “heterogeneous” was an awkward choice, as our intention was to highlight that certain associations attenuated more than others. We have rephrased this accordingly:

  **“This study also revealed varying confounding by specific cancer sites.”**

* ll.438-441: We do not fully agree that the results for colorectal cancer and head and neck cancer appear to be more robust. Please see our earlier comment and revise accordingly.

- **Response:** We have rephrased this part accordingly, and also refer to our earlier response regarding these findings:

  **“In contrast, associations with colon, head and neck, and oesophagus cancer seemed to attenuate less, although these associations were characterized by larger uncertainty.”**

**General Editorial Requests**

1) We ask every co-author listed on the manuscript to fill in a contributing author statement. If any of the co-authors have not filled in the statement, we will remind them to do so when the paper is revised. If all statements are not completed in a timely fashion this could hold up the re-review process. Should there be a problem getting one of your co-authors to fill in a statement we will be in contact. YOU MUST NOT ADD OR REMOVE AUTHORS UNLESS YOU HAVE ALERTED THE EDITOR HANDLING THE MANUSCRIPT TO THE CHANGE AND THEY SPECIFICALLY HAVE AGREED TO IT.

- **Response:** All authors have filled in this statement.

2) Please ensure that the paper adheres to the PLOS Data Availability Policy (see<http://journals.plos.org/plosmedicine/s/data-availability>), which requires that all data underlying the study's findings be provided in a repository or as Supporting Information. For data residing with a third party, authors are required to provide instructions with contact information for obtaining the data. PLOS journals do not allow statements supported by "data not shown" or "unpublished results." For such statements, authors must provide supporting data or cite public sources that include it.

- **Response:** We confirm the paper adheres to PLOS Data Availability Policy.

3) Please review your reference list to ensure that it is complete and correct. If you have cited papers that have been retracted, please include the rationale for doing so in the manuscript text, or remove these references and replace them with relevant current references. Any changes to the reference list should be mentioned in the rebuttal letter that accompanies your revised manuscript.

- **Response:** Check.
